# Association between the English National Health Service Diabetes Prevention Programme and incident multiple long-term conditions

**DOI:** 10.1038/s41591-025-03922-1

**Published:** 2025-09-10

**Authors:** Emma Barron, Paul Chappell, Izzy Hatfield, David C. D. Hope, Xiaochen Ge, Chirag Bakhai, Dominique Bradley, Ellie Bragan Turner, Rupert Dunbar-Rees, Nasrin Hafezparast, Desmond G. Johnston, Gary Wainman, Adrian Pratt, Eirion T. Slade, Edward W. Gregg, Kamlesh Khunti, Jonathan Valabhji

**Affiliations:** 1 Department of Metabolism, Digestion and Reproduction, Faculty of Medicine, Imperial College London, London, UK; 2 Chelsea and Westminster Hospital NHS Foundation Trust, London, UK; 3 NHS England, London, UK; 4 Bedfordshire, Luton and Milton Keynes Integrated Care Board, Luton, UK; 5 Outcomes Based Healthcare, Ltd., London, UK; 6 Department of Diabetes & Endocrinology, St. Mary’s Hospital, Imperial College Healthcare NHS Trust, London, UK; 7 NHS Arden & GEM Commissioning Support Unit, Derby, UK; 8 RCSI University of Medicine and Health Sciences, Dublin, Ireland; 9 School of Public Health, Imperial College London, London, UK; 10 Diabetes Research Centre, University of Leicester, Leicester, UK

**Keywords:** Lifestyle modification, Type 2 diabetes

## Abstract

Existing evaluations of the National Health Service Diabetes Prevention Programme (NHS DPP) in England have demonstrated associated reductions in body weight, hemoglobin A1c and incident type 2 diabetes (T2D). In this study, we examined associations between completion of the NHS DPP and incidence of T2D and 30 other long-term conditions (LTCs), including LTCs considered linked to the programʼs interventional goals of body weight reduction, increased physical activity and improved diet quality (LTC-L) and LTCs considered to be possibly linked to those goals (LTC-PL). We found that completers of the NHS DPP had lower incidences of T2D, LTC-L and LTC-PL compared to non-attenders. Although these associations attenuated over time, they remained significant for all outcomes at 24 months with an odds ratio of 0.53 (95% confidence interval: 0.48–0.59) for T2D and rate ratios of 0.79 (0.74–0.84) and 0.80 (0.74–0.88) for LTC-L and LTC-PL, respectively. However, we were not able to directly conclude whether lower incidence rates were a direct result of completing the NHS DPP or due to residual bias stemming from unmeasured confounding and imprecision in the estimation of diagnosis.

## Abstract (web summary)

A retrospective, observational study shows that completion of a diabetes prevention program in England (the NHS Diabetes Prevention Programme) was associated with decreased incidence of type 2 diabetes and long-term conditions such as dementia and chronic obstructive pulmonary disease at 24 months.

## Main

Reduced cardiovascular mortality in people with diabetes over the last 30 years has resulted in increased longevity, whereas higher obesity rates have driven younger age of T2D onset, both factors resulting in longer exposure to the metabolic environment, with associated diversification of the causes of morbidity and mortality and increasing occurrence of multiple LTCs in individuals living with diabetes^1^. There is growing concern about the increased prevalence of the entity ‘multiple long-term conditions’ (MLTCs), also known as ‘multimorbidity’, which is defined as the coexistence of two or more chronic physical or mental health conditions, in the general population^2^. MLTCs are associated with poorer health outcomes and greater use of healthcare services^3–5^. Although this has led to the development, independent of diabetes status, of clinical guidelines for the management of MLTCs^6^ and of frameworks to support MLTC research^7^, prevention of MLTCs has received relatively little attention^8,9^.

In 2016, the NHS in England established the Healthier You NHS DPP to prevent or delay the onset of T2D in adults identified with non-diabetic hyperglycemia (NDH; hemoglobin A1c (HbA1c) of 42–47 mmol mol^−1^ (6.0–6.4%) or fasting plasma glucose of 5.5–6.9 mmol l^−1^), also known as prediabetes. Individuals were eligible for the NHS DPP if their latest HbA1c reading was 42–47 mmol mol^−1^, recorded within the last 12 months, and if they were aged 18 years or older and not pregnant. The intervention involves 9 months of behavioral support to lose weight, increase physical activity and improve diet quality, through at least 13 group-based face-to-face sessions constituting a minimum of 16 hours of contact time^10^; the choice of digital delivery of the same intervention is also now offered^11^. The NHS DPP achieved universal population coverage in England in just over 2 years, and, by March 2022, over one million people with NDH had been referred into the program. Program access was associated with a 7% reduction in incidence of T2D at population level over the 18-month period up to March 2020 compared to what would have been expected by extrapolating the trend prior to the establishment of the NHS DPP^12^. During the same period, a 31% reduced T2D incidence was found in those who completed the program versus those who did not complete the program^13^ as well as a 20% reduced T2D incidence in those with NDH who were referred to the program versus a matched control group of those with NDH who were not referred to the program^14^.

The NHS DPP may have benefits beyond the prevention of T2D, providing opportunities to address common antecedent risk factors for other LTCs earlier in the life course, at the stage of pre-disease (NDH rather than T2D), with the potential for additional economic gains, including reduced involuntary exclusion from employment, in those of working age^2,7^. We aimed to assess associations between NHS DPP completion and the subsequent incidence of 31 LTCs recorded in the National Bridges to Health Segmentation Dataset in England^15,16^, to which the Minimum Dataset of the NHS DPP was linked. LTCs were grouped based on whether evidence derived from a literature search suggested that they are etiologically linked (LTC-L) or possibly linked (LTC-PL), within any populations, to the programʼs interventional goals of body weight reduction, increased physical activity or improved diet quality. Conditions were categorized as LTC-L if supported by evidence from randomized controlled trials (RCTs) or meta-analysis of RCTs. Conditions were categorized as LTC-PL if supported by observational studies only or if there was an absence of evidence. Four LTCs were excluded where the investigators agreed that they were not plausibly linked to body weight reduction, increased physical activity or improved diet quality. The full search strategy and search results are presented in the Supplementary Text and in Supplementary Table 1. The list of conditions by group can be found in Table 1.

**Table 1:** List of the 35 conditions from the Segmentation Dataset split into the following groups: LTCs considered aetiologically linked (LTC-L) or possibly linked (LTC-PL) to body weight reduction, increased physical activity or improved diet quality

Etiologically linked	Possibly linked	Excluded
Atrial fibrillation	Alcohol dependence	Autism
Cancer	Asthma	Cystic fibrosis
Cerebrovascular disease	Bronchiectasis	Learning disability
Chronic kidney disease	Chronic pain	Sickle cell disease
Chronic liver disease	COPD	
Coronary heart disease	Epilepsy	
Dementia	Inflammatory bowel disease	
Depression	Multiple sclerosis	
Diabetes*	Neurological organ failure	
Frailty	Osteoarthritis	
Heart failure	Parkinson’s disease	
Hypertension	Pulmonary heart disease	
Osteoporosis	Rheumatoid arthritis	
Peripheral vascular disease	Sarcoidosis	
Physical disability	Serious mental illness	
	Severe interstitial lung disease	

LTCs were excluded where the investigators agreed that they were not plausibly linked to behavior change.

*The NHS DPP and similar programs would be expected to have a preventative effect on T2D (which represents the vast majority of adult diabetes diagnoses), but diabetes is not included in the LTC-L grouping because the effect on T2D is being assessed separately.

We present the associations between completion of the NHS DPP (the intervention group) and the incidence of T2D, of two or more LTCs (MLTCs) and of both LTC-L and LTC-PL at 6 months, 12 months, 18 months and 24 months, using separate cohorts for each time period, compared to matched control groups of individuals referred to the NHS DPP who did not attend any sessions (the control group).

## Results

The number of individuals in the intervention group compared to the corresponding number of individuals in the control group was 56,940 versus 56,315 in the 6-month cohort, 38,140 versus 39,773 in the 12-month cohort, 22,497 versus 26,600 in the 18-month cohort and 11,821 versus 14,594 in the 24-month cohort. Differences between the group sizes were due to the fact that matching was undertaken at the point of NDH diagnosis rather than at the point of program initiation. The full selection process and the list of exclusion criteria are outlined in Supplementary Fig. 1. Analysis of standardized mean difference (SMD) scores indicated that broadly good balance was achieved across all matching variables at all follow-up periods (Supplementary Fig. 2).

Baseline characteristics for the 6-month cohort are shown in Supplementary Table 2. Subsequent follow-up period cohorts showed similar patterns and are omitted for brevity. In the 6-month cohort, for those in the intervention group, the mean age was 66 years, 45% were men, 6.9% were of Asian ethnicity, 5.0% were of Black ethnicity and 78% were of White ethnicity. There were higher proportions of individuals from the least-deprived quintile compared to the most-deprived quintile (26% versus 13%, respectively). Nearly 60% (57.6%) had at least one LTC at the start of follow-up; 35.5% had hypertension; 15.7% had osteoarthritis; and 13.6% had coronary heart disease. Men had higher proportions of coronary heart disease (19.7% for men versus 8.5% for women), whereas women had higher proportions of osteoarthritis (17.3% versus 13.9%) and depression (9.4% versus 5.6%) (Supplementary Table 3). Nearly 70% (69.6%) of older individuals (≥70 years) had at least one LTC: 49% with a diagnosis of hypertension. Younger individuals (<70 years) had higher proportions of depression (9.6% versus 5.0%) (Supplementary Table 4). Characteristics of individuals in the matched control group followed similar patterns.

### Incidence of MLTCs

**Fig. 1:**
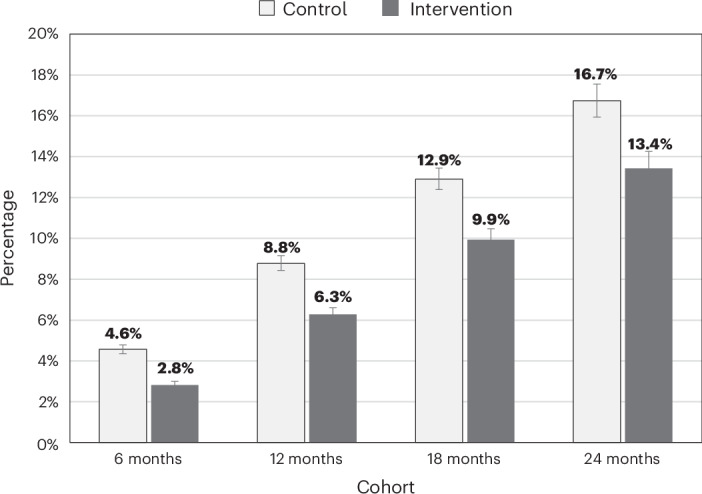
MLTC incidence at 6-month, 12-month, 18-month and 24-month follow-ups with 95% confidence intervals. Data are presented as proportions with 95% confidence intervals. *n* (intervention and control) = 36,836 and 36,625 at 6 months, 24,793 and 26,092 at 12 months, 14,730 and 17,655 at 18 months and 7,766 and 9,799 at 24 months.

At start of the follow-up period, in both the intervention and control groups, a third of individuals had at least two LTCs (Supplementary Table 5), constituting MLTCs. Individuals with MLTCs were older and had high proportions of hypertension (75.4% for those in the intervention group with at least two LTCs versus 13.8% for zero or one), osteoarthritis (36.8% versus 4.2%) and coronary heart disease (34.2% versus 2.3%) (Supplementary Table 6). Incidence of MLTCs (that is, moving from zero or one LTC to two or more LTCs during the follow-up period) was significantly lower in the intervention group compared to the control group at all four follow-up points with unadjusted odds ratios of 0.61 (0.56–0.66), *P* < 0.001; 0.70 (0.65–0.74), *P* < 0.001; 0.74 (0.69–0.80), *P* < 0.001; and 0.77 (0.71–0.84), *P* < 0.001 (Fig. 1 and Supplementary Table 5), respectively.

### New-onset T2D

**Fig. 2:**
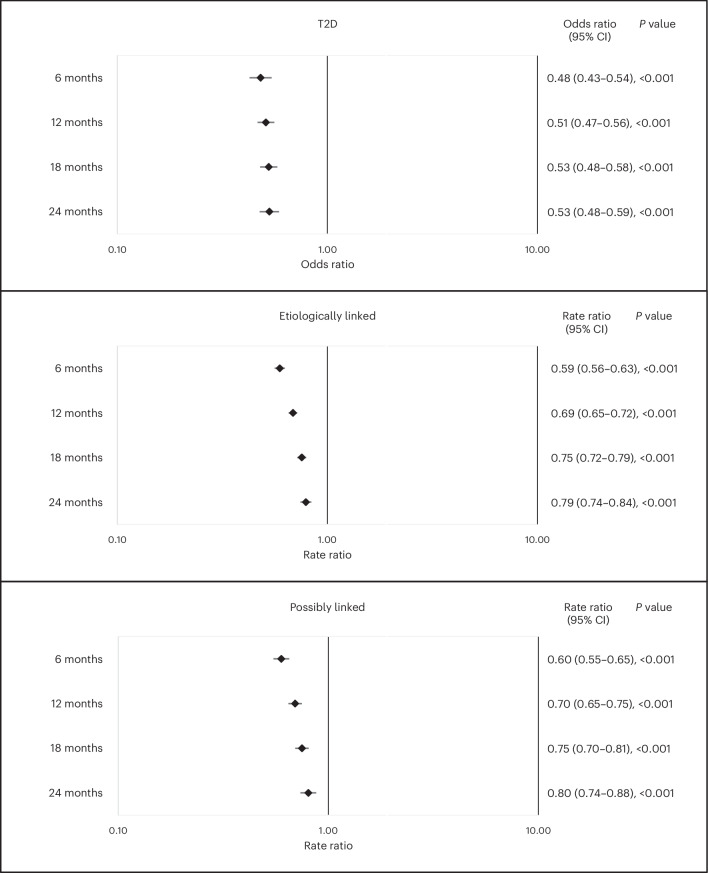
Primary analysis: adjusted estimates of incidence of T2D and LTC-L and LTC-PL at 6-month, 12-month, 18-month and 24-month follow-up periods with 95% CIs. Data are presented as odds ratios (diabetes) and rate ratios (LTC-L and LTC-PL) with 95% confidence intervals (CIs). *P* values are two-sided and were determined by logistic regression (diabetes) and negative binomial regression (LTC-L and LTC-PL), with no adjustments for multiple tests. *n* (intervention and control) = 56,940 and 56,315 at 6 months, 38,140 and 39,773 at 12 months, 22,497 and 26,600 at 18 months and 11,821 and 14,594 at 24 months.

At 6 months, 12 months, 18 months and 24 months, 0.7% (394), 1.8% (703), 3.0% (686) and 4.8% (562) of individuals, respectively, in the intervention group were recorded with a new diagnosis of T2D compared to 1.4% (816), 3.6% (1,419), 5.5% (1,473) and 8.7% (1,265) of individuals, respectively, in the control group (Supplementary Table 7). Compared to those in the control group, the adjusted odds ratios for incident T2D in the intervention group were 0.48 (95% confidence interval: 0.43–0.54, *P* < 0.001) at 6 months, 0.51 (0.47–0.56, *P* < 0.001) at 12 months, 0.53 (0.48–0.58, *P* < 0.001) at 18 months and 0.53 (0.48–0.59, *P* < 0.001) at 24 months (Fig. 2 and Supplementary Table 8). Adjusted odds ratios were similar between sex and age group (Supplementary Figs. 3 and 4).

### LTC-L

At least one new diagnosis of an LTC-L was recorded in the intervention group for 4.3% (2,443), 8.8% (3,366), 13.0% (2,935) and 16.9% (2,001) of individuals at 6 months, 12 months, 18 months and 24 months, respectively, compared to 6.5% (3,651), 11.9% (4,719), 16.4% (4,370) and 20.5% (2,995) of individuals, respectively, in the control group (Supplementary Table 7). Compared to those in the control group, the adjusted rate ratios for new diagnoses of LTC-L for those in the intervention group were 0.59 (95% confidence interval: 0.56–0.63, *P* < 0.001) at 6 months, 0.69 (0.65–0.72, *P* < 0.001) at 12 months, 0.75 (0.72–0.79, *P* < 0.001) at 18 months and 0.79 (0.74–0.84, *P* < 0.001) at 24 months (Fig. 2 and Supplementary Table 8). Adjusted rate ratios were similar by sex (Supplementary Fig. 3) but differed by age group, with greater differences for those aged 70 years or older compared to those younger than 70 years (Supplementary Fig. 4).

**Fig. 3:**
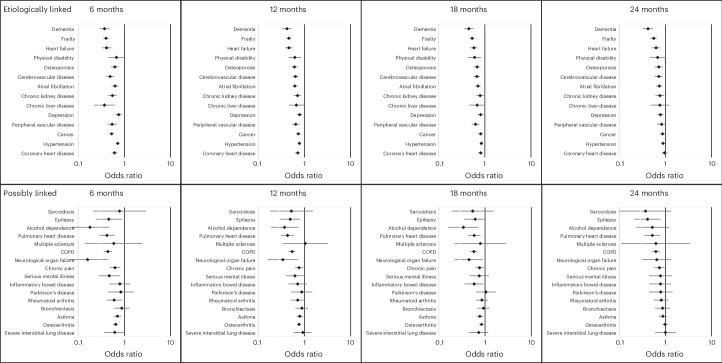
Odds of participants acquiring new LTCs, by condition, at 6-month, 12-month, 18-month and 24-month follow-up periods with 95% confidence intervals. Data are presented as odds ratios with 95% confidence intervals. *n* (intervention and control) = 56,940 and 56,315 at 6 months, 38,140 and 39,773 at 12 months, 22,497 and 26,600 at 18 months and 11,821 and 14,594 at 24 months.

New diagnoses most frequently occurred for hypertension, cancer and frailty (Supplementary Table 9), with significantly higher incidence in the control group, although there was some variation by sex and by age, with higher incidence of coronary heart disease for men and higher incidence of frailty for older individuals (Supplementary Tables 10 and 11). However, hypertension had one of the smallest differences relative to controls, with the magnitude of the difference decreasing over time. At 24 months, there was a significantly higher incidence in the control group for the majority (10/14) of conditions, with the exceptions being coronary heart disease, peripheral vascular disease, physical disability and chronic liver disease (Fig. 3 and Supplementary Table 9). For men, however, only dementia and frailty remained significant at 24 months, whereas the majority (10/14) remained significant for women, with heart failure and dementia having the greatest differences relative to controls (Supplementary Table 10 and Supplementary Fig. 5). For younger individuals, only depression remained significant at 24 months, whereas, for older individuals, eight out of 14 conditions remained significant, with dementia and frailty having the greatest differences relative to controls (Supplementary Table 11 and Supplementary Fig. 6).

### LTC-PL

At least one new diagnosis of an LTC-PL was recorded in the intervention group for 1.6% (921), 3.6% (1,372), 5.7% (1,288) and 7.9% (928) of individuals at 6 months, 12 months, 18 months and 24 months, respectively, compared to 2.7% (1,507), 5.1% (2,027), 7.6% (2,016) and 9.6% (1,405) of individuals, respectively, in the control group (Supplementary Table 7). Compared to those in the control group, the adjusted rate ratios for new diagnoses of LTC-PL for those in the intervention group were 0.60 (95% confidence interval: 0.55–0.65, *P* < 0.001) at 6 months, 0.70 (0.65–0.75, *P* < 0.001) at 12 months, 0.75 (0.70–0.81, *P* < 0.001) at 18 months and 0.80 (0.74–0.88, *P* < 0.001) at 24 months (Fig. 2 and Supplementary Table 8). Adjusted rate ratios were similar by sex and by age group (Supplementary Figs. 3 and 4).

New diagnoses most frequently occurred for osteoarthritis, asthma, chronic obstructive pulmonary disease (COPD) and chronic pain in both the intervention and control groups, with higher incidence in the control group (Supplementary Table 9). The incidence of other conditions was small (*n* < 100 at all follow-up points). Osteoarthritis and asthma, in particular, had among the smallest differences relative to controls, with the magnitude of the difference decreasing over time. At 24 months, only COPD, pulmonary heart disease, epilepsy and chronic pain had a significantly lower incidence in the intervention group compared to the control group (Fig. 3). Results were similar by age and sex (Supplementary Tables 10 and 11 and Supplementary Figs. 5 and 6).

### Secondary analyses that assessed individuals not referred to the NHS DPP as the control group

In secondary analyses with a different matched control group (individuals diagnosed with NDH but not referred to the NHS DPP) (Supplementary Tables 12–14), completion of the NHS DPP was found to be significantly associated with lower incidence of new diagnoses of T2D, new diagnoses of LTC-L and new diagnoses of LTC-PL at all follow-up points (Supplementary Table 15). MLTC incidence was also significantly lower in the intervention group compared to the control group at all four follow-up points (all *P* < 0.001) (Supplementary Table 16).

## Discussion

This retrospective observational study shows that completion of the NHS DPP, a diabetes prevention program that targets body weight reduction, increased physical activity and improved diet quality, is associated with lower incidence of T2D and LTCs etiologically linked and possibly linked to these program interventional goals. The association decreased in magnitude over the longer follow-up periods but remained significant for each of the three outcome measures at 24 months. Incidence of MLTCs was significantly lower in the intervention group compared to the control group at all four follow-up points. However, the presence of biases arising from unmeasured differences between the intervention and control groups cannot be ruled out.

Weight loss and HbA1c reduction associated with completion of the NHS DPP has been demonstrated (mean weight loss of 3.3 kg and mean HbA1c reduction of 2.0 mmol mol^−1^ in those who complete the intervention)^10^. Although weight change and HbA1c change were not recorded for individuals in the control groups, greater weight loss and HbA1c reduction in the intervention compared to the control groups in this study is likely. Obesity has been recognized as a potentially important interventional target to prevent subsequent MLTCs: in an observational study, obesity was associated with 21 non-overlapping cardiometabolic, digestive, respiratory, neurological, musculoskeletal and infectious diseases^17^. Another observational study found weight loss to be associated with risk reductions for T2D, hypertension, dyslipidemia, chronic kidney disease, sleep apnea and asthma^18^. The Look-AHEAD trial found a 7% reduction in weight loss and improved cardiovascular fitness in middle-aged individuals with T2D undergoing an intensive lifestyle intervention. Although no differences were found in the primary endpoint of cardiovascular outcomes, a post hoc analysis found that the rate of increase in MLTCs over time was slowed^19^. In the 30-year follow-up of the Da Qing Diabetes Prevention Outcome Study, although MLTCs were not quantified, those with impaired glucose tolerance who had taken part in a lifestyle program still had a lower incidence of T2D in addition to a reduced incidence of cardiovascular events, microvascular complications and cardiovascular and all-cause mortality^20^.

To our knowledge, our study is the first to examine the association of the NHS DPP with incidence of T2D alongside incidence of other LTCs and with incidence of MLTCs. Our results are consistent with previous research showing an association between completion of the NHS DPP and reduced T2D incidence^12–14^. There is a lack of RCTs of interventions designed to prevent MLTCs, although there is existing evidence on the development of LTCs other than T2D. A Finnish RCT suggested that a multidomain intervention over 2 years, including nutritional guidance, exercise, cognitive training and management of metabolic and vascular risk factors, reduced the risk of developing new chronic diseases in people aged 60–77 years^21^.

The 50% reduction in T2D incidence compares those who completed the program (defined as attending at least 60% of intervention sessions) to those who were referred to the program but did not attend any intervention sessions, whereas the 31% reduction in T2D incidence compares those who completed the program to those who did not complete it. The not-completed group includes individuals who attended 1–7 intervention sessions, whereas the not-attended group includes only those who attended zero intervention sessions.

A dose–response relationship was observed between the number of sessions attended and the incidence of T2D^13^. We would, therefore, expect a greater reduction in T2D incidence between those who completed the program and those who did not attend intervention sessions than would be observed when comparing those who completed the program to those who did not complete it. However, there are also likely unmeasured confounders that we are unable to control for. For example, it is possible that participants who were less well failed to complete by dropping out early.

Our study has several limitations. The study uses observational data collected during routine care delivery, and there is no randomization to participation in the NHS DPP. We used matching to create a comparison control group, which was as close to the intervention population as possible in key observed characteristics, except for the absence of exposure to the DPP. In principle, this would allow us to attribute any difference in outcomes to the impact of the DPP; in practice, however, there are likely to be a variety of unmeasured confounders that we are unable to control for. For example, we were unable to adjust for body mass index, HbA1c, smoking status and family history of diabetes due to absence, or poor ascertainment, of these parameters in the datasets that we used.

It is possible that the similarity of results between the LTC-L and LTC-PL groups indicates the presence of unobserved bias, and it is also possible that the LTC-PL group is acting as a negative control more generally. Although it is plausible that observed reductions in some conditions such as depression, chronic pain and hypertension may be attributable to the lifestyle intervention, the timing of the observation period (up to 2 years) makes such a direct relationship for two of the conditions with the largest effect sizes, frailty and dementia, less likely. For example, one study found differences in levels of cardiometabolic factors up to two decades prior to dementia diagnosis^22^. A possible interpretation in these cases is reverse causation, with individuals who develop these conditions being unlikely to, or unable to, complete the program.

The National Bridges to Health Segmentation Dataset is derived through linkage of several datasets, including the Community Services Dataset and the Secondary Uses Services (SUS) dataset, but these source datasets are mostly secondary care sources (Supplementary Table 17). Conditions that are usually diagnosed in primary care, such as depression and hypertension, are known to have lower ascertainment in the Segmentation Dataset than in primary care datasets (Supplementary Text). This measurement imprecision may lead to bias and unobserved confounding if the effect is greater for control or intervention groups. Another potential confounding factor is the possibility of a greater latent prevalence of LTCs in the control group compared to the intervention group. We have matched on observed prevalence at NDH diagnosis, but some individuals may have latent LTCs that are either undiagnosed or not yet coded within the data. These latent cases may appear as false incident cases during follow-up if they are subsequently diagnosed or coded. If latent cases of a given condition are more common in the control group at baseline, this may lead to a greater number of falsely incident cases between the groups, particularly early in the follow-up period. T2D diagnoses are identified using the National Diabetes Audit (NDA) dataset, so this effect will be minimal for T2D.

It is also possible that those who choose to attend and complete the program (that is, the intervention group) are particularly motivated and exhibit behaviors meaning that they are less likely to develop any new incident conditions a priori. On the other hand, new diagnoses may be more likely to be coded and recorded in the intervention group, as they may be more engaged with healthcare.

Additionally, the NDA dataset has a degree of under-coding of NDH. Approximately 26% of those who were referred to the NHS DPP did not have an NDH code and, therefore, an NDH date of diagnosis, requiring us to estimate the date of NDH diagnosis for these individuals, potentially leading to further bias. The intervention and control groups also differ in size, particularly in the longer follow-up periods, due to greater volumes of missing data in the control group in the outcome variables derived after matching. Although the matching process was selected to minimize bias, the possibility that data exclusions in the follow-up period were not missing at random must be acknowledged.

In conclusion, we found a significant association between completion of the NHS DPP and lower incidence of T2D, MLTCs as well as conditions considered etiologically linked to the programʼs interventional goals and conditions possibly linked to the programʼs interventional goals. It is not possible to draw conclusions on the extent to which lower incidence rates were a direct consequence of the NHS DPP as opposed to limitations of the data and potential bias stemming from unmeasured differences between the intervention and control groups, and, therefore, the causal pathways through which reduced incidence rates were achieved are unclear. Recently, published UK policy documents have highlighted the important impacts of both MLTCs and associated preventative approaches in supporting the sustainability of future healthcare delivery^23,24^. Evidence has already demonstrated the NHS DPP to be cost-effective by 12 months^25^ and, taking incidence of T2D but not incidence of other LTC incidence into account, cost-saving by year 9 (ref. ^26^). Further high-quality studies, including RCTs, are warranted to determine if such real-world lifestyle interventions can reduce the risk of development of other LTCs and MLTCs.

## Methods

### Study design and data sources

The data used in this study are collected and used in line with NHS England’s purposes, as required under the statutory duties outlined in the NHS Act 2006 and the Health and Social Care Act 2012. The Segmentation Dataset is processed under a data processing agreement between NHS England and Outcomes Based Healthcare, Ltd. (OBH), which produces the Segmentation Dataset on behalf of NHS England. This ensures controlled access by appropriate approved individuals to anonymized/pseudonymized data held on secure data environments entirely within the NHS England infrastructure. Data are processed for specific preregistered purposes only, including operational functions, service evaluation and service improvement. The present study supported these purposes, so ethics committee approval was not required. Where OBH had processed data, this was agreed and detailed in a data processing agreement. The data used to produce this analysis were disseminated to NHS England under directions issued under Section 254 of the Health and Social Care Act 2012.

This was a retrospective observational cohort study that aimed to assess the association between completion of the NHS DPP (as measured through attendance at more than 60% of sessions) and subsequent incidence of LTCs recorded, including T2D, between April 2016 and February 2020. After exclusion of four LTCs usually diagnosed at birth or in early childhood, 31 LTCs were divided into two groups: LTCs considered etiologically linked to program interventional goals of body weight reduction, increased physical activity or improved diet quality (LTC-L) and LTCs considered to be possibly linked to body weight reduction, increased physical activity or improved diet quality (LTC-PL). This categorization was based on a search strategy detailed in Supplementary Table 1 and Supplementary Information (‘Search strategy for linked and possibly linked conditions’). The full list of conditions can be found in Table 1.

The NDA was used to identify all individuals diagnosed with NDH and was also used to measure incidence of T2D. The NDA has collated data on people with diabetes registered with a general practice in England since 2003 and with NDH since 2017, with almost complete practice participation in recent years (99% in 2019/2020). However, there is a degree of under-coding of NDH in general practice^27^. The NHS DPP Minimum Dataset was used to identify all individuals with NDH who had been referred to the NHS DPP.

The Bridges to Health Segmentation Dataset was used to measure the incidence of the 30 other LTCs. The dataset covers individuals registered with a general practice in England since 2014 and includes sociodemographic, geographic as well as clinical diagnostic data. The Segmentation Dataset has been derived from numerous national, predominately secondary care, patient-level datasets held in the National Commissioning Data Repository, each of which is linked by pseudonymized NHS numbers. The dataset now includes more than 15 years of data from the SUS dataset, which collects data from all hospitals in England, including admitted patient care, outpatient and emergency care data, in addition to mental health data, community data and the NDA. The full list of source datasets used to derive the Segmentation Dataset, including the time over which data have been longitudinally accrued, can be found in Supplementary Table 17. The Segmentation Dataset includes 35 LTCs, including diabetes, which align reasonably well with the conditions suggested for inclusion in the entity ‘multiple long-term conditions’ through a recent Delphi consensus study^28^. The 35 conditions included in the Segmentation Dataset were derived using data definitions based on logic and clinical codes (for example, International Classification of Diseases, 10th revision, diagnostic codes; OPCS Classification of Interventions and Procedures codes; and Systematized Nomenclature of Medicine–Clinical Terms codes) and were developed for each condition after extensive clinical review and evaluation^29^. Further details on the process of selection of LTCs and the prevalence of MLTCs were published previously^30^.

The NDA dataset was used to measure incidence of T2D because the Segmentation Dataset is derived through linkage of numerous datasets, not all of which identify type of diabetes. Further information on the accuracy of the coding in the Segmentation Dataset is outlined in Supplementary Information (‘Further infomation on the National Segmentation Dataset’).

SUS data were used to estimate the frequency with which individuals attended accident & emergency (A&E) departments, were admitted to hospital as inpatients or had an appointment as outpatients. Publicly available data sources were used to provide information on the Index of Multiple Deprivation (IMD)^31^; workforce characteristics^32^; pay for performance, also known as Quality and Outcomes Framework (QOF) scores^33^; and the Rural–Urban Classification^34^ of each individual’s general practice.

### Outcomes

The outcome assessed was the incidence of T2D and 30 other LTCs between 1 April 2016 and 28 February 2020 at timepoints of 6 months, 12 months, 18 months and 24 months after each participant’s start of the NHS DPP, using separate cohorts for each time period. In primary analyses, individuals who had been referred to the NHS DPP and completed the program, defined as attending more than 60% of intervention sessions (the intervention group), were compared to a matched control group of individuals who were referred to the NHS DPP but did not attend any program sessions. In secondary analyses, analyses were repeated, with the matched control group consisting of individuals with NDH who were not referred to the NHS DPP. Individuals who were referred to the program and attended less than 60% of the program were not included. We also assessed MLTC incidence by comparing the proportions of individuals who transitioned from a non-MLTC state (that is, fewer than two LTCs at baseline) to an MLTC state (that is, two or more LTCs at follow-up), in both the primary and secondary analyses.

### Covariates

Each individual in the study was assigned a date of NDH diagnosis and a start date for the follow-up period. The start date of the follow-up period refers to the date of the first intervention session attended or is estimated for those who did not attend the NHS DPP. NDH diagnosis dates that were missing, either by not being recorded in the NDA or recorded incorrectly as occurring after their start of the follow-up period, were estimated by randomly sampling from the distribution of the lags (in days) between valid NDH diagnosis dates and valid start dates and subtracting this number of days from the start date. Of those in the primary analyses in the 6-month cohort (*n* = 113,255), 37.0% (*n* = 41,932) of individuals had estimated NDH diagnosis dates, of which 26.2% (*n* = 29,745) were missing from the NDA and 10.8% (n = 12,187) had NDH diagnosis dates that occurred after NHS DPP start dates. In the 6-month cohort, the median (interquartile range) number of days between the NDH diagnosis date and the start date of the NHS DPP was 169 days (84–361) for those in the intervention group and 162 days (81–347) for those in the control group. Results were similar in the 12-month, 18-month and 24-month cohorts.

Demographic factors age, sex, ethnicity and socioeconomic status were identified as potential confounding factors. Sex was recorded as male or female. Age in years was calculated as at the date of NDH diagnosis. Socioeconomic status was measured using IMD quintiles associated with the Lower Layer Super Output Area of residence, which was derived from individual postcode. For ethnicity, each individual was assigned their most frequently recorded ethnicity across source datasets and then allocated to one of the conventional high-level groupings: Asian, Black, Mixed, Other, White and unknown.

The sex variable in this study is derived from the ‘administrative gender’ field of the Master Patient Index/Personal Demographics Service source dataset. Among a small proportion of people, estimated by the UK census to be 0.5%, for whom gender identity and biological sex at birth are not the same, a further unquantifiable proportion may have an ‘administrative gender’ code that differs from their biological sex. This proportion depends on how this field is used, which is likely to be inconsistent between providers, and is not currently possible to establish based on the available data. We understand that this is symptomatic of wider challenges with confusion around sex and gender data collection in NHS datasets, as recently highlighted in the March 2025 independent review of data, statistics and research on sex and gender^35^. This review also made recommendations for improved clarity in future data collection.

We also included clinical factors: the presence of preexisting LTCs and the frequency of emergency admissions to hospital, outpatient appointments and A&E attendances in the year before NDH diagnosis. The following general practice–level factors were also included to capture the sociodemographic context and quality of primary care: IMD quintile of general practice, rural–urban classification of general practice, overall clinical QOF score, clinical commissioning group of practice, size of practice list and full-time equivalent (FTE) general practice ratio. To account for the selection bias likely stemming from clinical judgment subjectively informing referral decisions across different general practices, we also derived a variable that estimated the probability of program completion for individuals referred to the program.

### Matching

We used the nearest neighbor matching algorithm without replacement^36^ on the demographic, clinical, general practice and probability of completion factors listed above, determined at the date of NDH diagnosis, with a 1:1 ratio of control to intervention units. To account for varying lags between date of NDH diagnosis and start of the program, matching also incorporated the quarter of date of NDH diagnosis.

Matching was conducted in four separate waves, corresponding to the four cohorts, starting with 24 months and moving on to 18 months, 12 months and 6 months, at each stage retaining only the individuals who were eligible for the program and had enough follow-up time in the study period (that is, had started the NHS DPP intervention before March 2018 for the 24-month follow-up period, before September 2018 for the 18-month follow-up period, before March 2019 for the 12-month follow-up period and before September 2019 for the 6-month follow-up period). Balance was assessed using SMD scores^37^. The full selection process and the list of exclusion criteria are outlined in Supplementary Fig. 1.

### Statistical analysis

Logistic regression models were used to estimate the association between completion of the NHS DPP and incidence of T2D in all four cohorts. For example, in the 6-month cohort, the incidence of T2D up to 6 months after starting the program for individuals in the intervention group was compared to the incidence of T2D for individuals in the control group over the same time period. The first model included only the group identifier (intervention or control) as an independent variable to calculate unadjusted odds ratios for the incidence of T2D. The second model additionally included the demographic, clinical, general practice and probability of completion factors as independent variables to calculate adjusted odds ratios. Preexisting LTCs were determined at the start of the follow-up period, whereas other factors were determined at the NDH diagnosis date. Together, these are referred to as the baseline characteristics. Corresponding analyses at 12 months, 18 months and 24 months were similarly carried out. We also ran separate logistic regression models for each LTC, with the incidence of each condition (zero or one) as the outcome.

Separate negative binomial regression models were used to estimate the association between completion of the NHS DPP and the number of new LTC-L and LTC-PL in all four cohorts. For example, in the 6-month cohorts, negative binomial regressions were fitted with the number of new conditions acquired up to 6 months after starting the program as the outcome. The first set of models included only the group identifier (intervention or control) as an independent variable to calculate unadjusted rate ratios for the incidence of LTC-L conditions and LTC-PL conditions. The second model additionally included the demographic, clinical, general practice and probability of completion factors as independent variables to calculate adjusted rate ratios. Corresponding analyses at 12 months, 18 months and 24 months were similarly carried out. As above, preexisting LTCs were determined at the start of the follow-up period, whereas other factors were determined at the NDH diagnosis date.

Separate models were run by sex and age group (<70 years and ≥70 years). Overlapping confidence intervals were used to assess any differences between the models. Model fit was verified using standard diagnostic statistics (Akaike information criterion, root mean square deviation, overdispersion and percentage observed versus predicted zeros). Chi-square tests for categorical data and *t*-tests for continuous data were used to assess any differences between the intervention and control groups.

Statistical significance was defined as *P* < 0.05, and confidence intervals were set at 95%. All data were analyzed with RStudio version 4.4.1.

### Reporting summary

Further information on research design is available in the Nature Portfolio Reporting Summary linked to this article.

## Online content

Any methods, additional references, Nature Portfolio reporting summaries, source data, extended data, supplementary information, acknowledgements, peer review information; details of author contributions and competing interests; and statements of data and code availability are available at 10.1038/s41591-025-03922-1.

## Supplementary information

Supplementary Information Supplementary text, Supplementary Tables 1–17 and Supplementary Figs. 1–6

Reporting Summary

## Data Availability

The source data used in this evaluation and to derive the National Bridges to Health Segmentation Dataset are from the NHS National Commissioning Data Repository (NCDR). The NCDR is a pseudonymized patient-level data repository managed by the NHS England Data Services team. It is used by NHS England for operational service improvement and service evaluation purposes. It is designed to deliver consistent data processing, linkage and reporting services, mainly for the use of NHS England analytical teams to aid in the delivery of a wide range of projects. The data enable analysts to provide evidence in the form of reports and dashboards to support the NHS drive to improve health and well-being across England. Data Services was established by NHS England to ensure that information about the performance and impact of NHS services is available when decisions are made about the commissioning of health services. Data Services also ensures that information has been accessed legally in accordance with all applicable laws and mandatory guidance contained within the Health and Social Care Act 2012, the Care Act 2014, the Data Protection Act 2018 and the UK General Data Protection Regulation. The authors cannot provide direct access to the data, as this would circumvent NHS England’s research data access procedures. More information about the NCDR and how to contact NHS England Data Services is available at https://webarchive.nationalarchives.gov.uk/ukgwa/20231101051610/https://data.england.nhs.uk/ncdr/database/.

## Code availability

The code will be available in a GitHub repository at https://github.com/nhsengland/NHS-DPP-multiple-long-term-conditions-evaluation-27-06-2025.
