# Supplementary material for: Association between the English National Health Service Diabetes Prevention Programme and incident multiple long-term conditions
Source: Nat Med. 2025 Sep 10;31(11):3825–31. doi: 10.1038/s41591-025-03922-1 (PMC12618231; doi:10.1038/s41591-025-03922-1)
Supplement: Supplementary file 1 — Supplementary text, Supplementary Tables 1–17 and Supplementary Figs. 1–6 [file 41591_2025_3922_MOESM1_ESM.pdf]

nature medicine

Supplementary information https://doi.org/10.1038/s41591-025-03922-1

Association between the English National Health Service Diabetes Prevention Programme and incident multiple long-term conditions

In the format provided by the authors and unedited

Supplementary

Supplementary Text

Search strategy for ‘linked’ and ‘possibly linked’ conditions

A search of the PubMed MEDLINE database (https://pubmed.ncbi.nlm.nih.gov) was carried out to identify articles published in the last 15 years (From January 2010). The search strategy included phrases and key words related to diet, physical activity or body weight loss, in any population, with the development of each long-term condition. The first group of long-term conditions included those where development of the LTC was considered aetiologically linked (LTC-L) to diet, increased physical activity or body weight reduction if supported by evidence from randomised control trials (RCT’s) or meta-analysis of RCT’s. A second group of long-term conditions included those where development of the LTC was considered possibly linked (LTC-PL) to diet, increased physical activity or body weight reduction if supported by evidence from observational studies or if there was an absence of evidence. Long-term conditions were excluded where the investigators agreed that they were not plausibly linked to these behavioural changes; Excluded LTC’s included learning disabilities, cystic fibrosis, sickle cell anaemia and autism. Case-reports and conference abstracts were not included. Animal studies were excluded. Article type filters were applied to the broad search strategy to identify relevant articles listed in table 1 below.

A search for articles using the PubMed MEDLINE database April 2025. The following search strategy and string were used:

1. The Intervention (1a DIET OR 1b PHYSICAL ACTIVITY OR 1c BODY WEIGHT LOSS)
2. Outcome (2a INCIDENCE of 2b CONDITION ‘X’)

1.Search for the intervention

1a Diet: "healthy diet*"[tiab] OR "healthy food"[tiab] OR "healthy eating" [tiab] OR "healthier food"[tiab] OR "healthier diet"[tiab] OR "lifestyle changes"[tiab] OR "mediterranean diet"[tiab] OR "low calorie diet"[tiab] OR "calorie restriction"[tiab] OR diet[MeSH]

OR

1b Physical activity: exercise*[tiab] OR "physical activity"[tiab] OR exercise[MeSH]

OR

1c Body weight loss: "weight loss"[tiab] OR "weight reduction"[tiab] OR "intensive lifestyle"[tiab] OR "weight loss"[MeSH]

AND

2.Search strategy for incidence of condition

2a Incidence: incidence[tiab] OR "incident risk"[tiab] OR onset[tiab] OR develop*[tiab] OR incidence[MeSH]

AND

2b ‘X’ condition: *Condition 1*: ("atrial fibrillation"[tiab] OR "atrial fibrillation"[MeSH]) *Condition 2:* (cancer[tiab]) *Condition 3:* ("cerebrovascular disease"[tiab] OR stroke[tiab] OR stroke[MeSH]) *Condition 4* ("chronic kidney disease"[tiab] OR "kidney disease"[tiab] OR "kidney failure, chronic"[MeSH])

Condition 5: ("chronic liver disease"[tiab] OR "liver failure"[tiab] OR "fatty liver"[tiab] OR "liver failure"[MeSH]) *Condition 6:* ("chronic pain"[tiab] OR "chronic pain"[MeSH]) *Condition 7:* ("coronary heart disease"[tiab] OR "myocardial infarction"[tiab] OR "coronary artery disease"[MeSH]) *Condition 8:* (dementia[tiab] OR dementia[MeSH]) *Condition 9:* (depression[tiab] OR depression[MeSH]) *Condition 10:* (diabetes[tiab] OR "type 2 diabetes"[tiab]) *Condition 11:* ("heart failure"[tiab] OR "heart failure"[MeSH]) *Condition 12:* (hypertension[tiab] OR hypertension[MeSH]) *Condition 13:* (frailty[tiab] OR frailty[MeSH]) *Condition 14:* (osteoporosis[tiab] OR "bone mineral density"[tiab] OR osteoporosis[MeSH]) *Condition 15:* ("peripheral vascular disease"[tiab] OR "peripheral arterial disease"[tiab] OR "peripheral arterial disease"[MeSH]) *Condition 16:* ("physical disability"[tiab]) *Condition 17:* ("alcohol dependence"[tiab] OR alcoholism[MeSH]) *Condition 18:* (asthma[tiab] OR asthma[MeSH]) *Condition 19:* (bronchiectasis[tiab] OR bronchiectasis[MeSH]) *Condition 20:* (COPD[tiab] OR "chronic obstructive pulmonary disease"[tiab] OR "pulmonary disease, chronic obstructive"[MeSH]) *Condition 21:* (epilepsy[tiab] OR epilepsy[MeSH]) *Condition 22:* ("inflammatory bowel disease"[tiab] OR "inflammatory bowel diseases"[MeSH]) *Condition 23:* ("multiple sclerosis"[tiab] OR "multiple sclerosis"[MeSH]) *Condition 24:* ("neurological organ failure"[tiab]) *Condition 25:* (osteoarthritis[tiab] OR osteoarthritis[MeSH]) *Condition 26:* ("parkinson's disease"[tiab] OR "parkinson disease"[MeSH]) *Condition 27:* ("pulmonary heart disease"[tiab] OR "pulmonary heart disease"[MeSH]) *Condition 28:* ("rheumatoid arthritis"[tiab] OR "arthritis, rheumatoid"[MeSH]) *Condition 29:* (sarcoidosis[tiab] OR sarcoidosis[MeSH]) *Condition 30:* ("serious mental illness"[tiab] OR "mental illness"[tiab]) *Condition 31:* ("severe interstitial lung disease"[tiab] OR "pulmonary fibrosis"[tiab] OR "lung diseases, interstitial"[MeSH]) *Condition 32:* ("chronic morbidity"[tiab] OR multimorbidity[tiab])

NOT (Animal[tiab] OR "animal study"[tiab])

Further information on the National Segmentation Dataset

The National Segmentation Dataset provides patient level longitudinal data with monthly granularity.

The accuracy of the timing of coding of clinical diagnosis, is condition-dependent, and depends on a number of factors. Because the National Segmentation Dataset does mainly not include primary care data, the timing of clinical coding is generally more accurate for conditions that commonly present first in secondary care. The effect of this on condition prevalence in the National Segmentation Dataset, compared to a local linked dataset based on linked primary care and Secondary Uses Service (SUS) data is presented on the Outcomes Based Healthcare website: https://outcomesbasedhealthcare.com/nhse-segmentation-dataset-reference-guide/#v-pills-comparison-to-primary-care-data.

The figure below (also shown on the Outcomes Based Healthcare website) compares the prevalence from the segmentation dataset with the prevalence from primary care and shows that for conditions which are commonly diagnosed in secondary care, such as cancer, cerebrovascular disease, chronic liver disease, coronary heart disease, and peripheral vascular disease, the prevalence is similar between the two datasets. Correspondingly, for conditions such as chronic kidney disease, depression, and hypertension, which are often managed in primary care alone in their early stages, the impact on recorded prevalence is higher. This effect is limited for certain conditions on account of the fact that the National Segmentation Dataset includes data from multiple datasets aside from SUS (https://outcomesbasedhealthcare.com/nhse-segmentation-dataset-reference-guide/#v-pills-data-sources). For example, the inclusion of data from the National Diabetes Audit, which takes data from primary care, means that diabetes prevalence is very similar between the two datasets. Overall, out of the 35 long term conditions included in this analysis, only 5 conditions have an **absolute** difference in prevalence of more than 2% between the two datasets: chronic kidney disease, depression,

hypertension, osteoarthritis, and physical disability. There is also a chart which displays the relative difference for each condition between each dataset.

Further analysis has been performed which compares prevalence figures from the National Segmentation Dataset against equivalent quality and outcome framework (QOF) condition registers, for the 16 conditions where such registers exist, with good concordance: https://outcomesbasedhealthcare.com/nhse-segmentation-dataset-reference-guide/#v-pills-comparison-to-QOF.

The absolute difference between condition prevalence figures from the National Segmentation Dataset and a local linked Segmentation Dataset that includes primary care data for a single geographical area

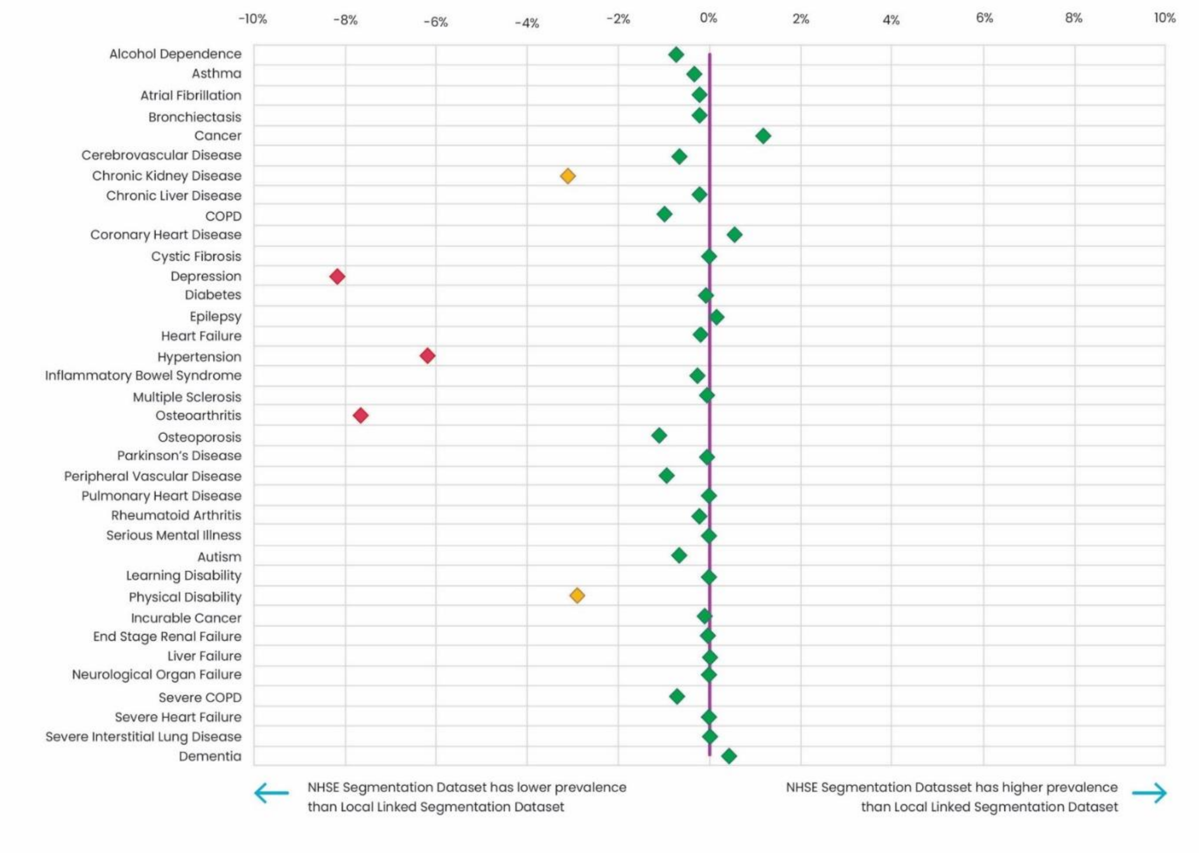

Supplementary Tables

Supplementary table S1: List of long-term conditions (LTCs) grouped based on PubMed MEDLINE database search accessed April 2025.
RCT, Randomised controlled trial; MA, Meta-analysis

Long-term conditions 'linked' (LTC-L)	Strength of evidence	Long-term conditions 'possibly linked' (LTL-PL)	Strength of evidence	Long-term conditions excluded
Cardiovascular		***Cardiovascular***		Autism
Cerebrovascular Disease	MA-RCT/RCT	Pulmonary Heart Disease	-	Cystic Fibrosis
Heart Failure	RCT	***Respiratory***		Learning Disability
Coronary Heart Disease*	MA-RCT/RCT	Asthma	MA-cohort/cohort	Sickle Cell Disease
Hypertension	MA-RCT/RCT	Bronchiectasis	-	
Atrial Fibrillation	RCT	Chronic obstructive pulmonary disease	Cohort	
Peripheral vascular disease	RCT	***Neurological***		
Metabolic		Epilepsy	Cohort	
Diabetes	MA-RCT/RCT	Multiple sclerosis	MA-cohort/case control	
Renal		**Musculoskeletal**		
Chronic Kidney Disease	RCT	Osteoarthritis	Cohort	
Gastrointestinal		***Other***		
Chronic Liver Disease	RCT	Sarcoidosis	-	
**Multisystem/other**		Alcohol Dependence	Cohort	
Frailty	MA-RCT/RCT	Serious Mental Illness	Cohort	
Physical Disability	RCT	Severe Interstitial Lung Disease	-	
Cancer	RCT	Neurological Organ Failure	-	
Depression	MA-RCT	Inflammatory Bowel Disease	MA-Cohort/cohort	
Musculoskeletal		Rheumatoid Arthritis	MA-Cohort/cohort	
Osteoporosis	MA-RCT/RCT	Parkinson's Disease	MA-Cohort	
Neurological		Chronic Pain	Cohort	
Dementia	RCT			

Supplementary table S2: Baseline characteristics of the 6-months cohort, for those referred to the NHS DPP and completed the programme (intervention group) and those who were referred to the NHS DPP but did not start the NHS DPP (control group)

	N		%		
	Referred to the NHS DPP but did not attend any intervention sessions	Referred to the NHS DPP and completed	Referred to the NHS DPP but did not attend any intervention sessions	Referred to the NHS DPP and completed	P value
Total	56,315	56,940	100%	100%	
Number of LTC_Ls					
0	28,818	28,379	51.2%	49.8%	
At least 1	27,497	28,561	48.8%	50.2%	<0.001
At least 2	13,602	13,697	24.2%	24.1%	0.699
At least 3	5,890	5,634	10.5%	9.9%	0.002
At least 4	2,409	2,134	4.3%	3.7%	<0.001
Number of LTC_PLs					
0	40,267	40,601	71.5%	71.3%	
At least 1	16,048	16,339	28.5%	28.7%	0.46
At least 2	4,340	4,368	7.7%	7.7%	0.823
At least 3	1,257	1,179	2.2%	2.1%	0.061
At least 4	328	275	0.6%	0.5%	0.021
Number of LTCs					
0	24,775	24,151	44.0%	42.4%	
At least 1	31,540	32,789	56.0%	57.6%	<0.001
At least 2	19,690	20,104	35.0%	35.3%	0.226
At least 3	10,717	10,665	19.0%	18.7%	0.197
At least 4	5,621	5,322	10.0%	9.3%	<0.001
**LTC_L**					
Atrial Fibrillation	3,846	3,894	6.8%	6.8%	0.95
Cancer	4,392	4,554	7.8%	8.0%	0.215
Cerebrovascular Disease	2,095	1,961	3.7%	3.4%	0.012
Chronic Kidney Disease	1,340	1,233	2.4%	2.2%	0.016
Chronic Liver Disease	306	282	0.5%	0.5%	0.26
Coronary Heart Disease	7,524	7,730	13.4%	13.6%	0.289
Dementia	514	432	0.9%	0.8%	0.004
Depression	4,566	4,393	8.1%	7.7%	0.014
Frailty	913	630	1.6%	1.1%	<0.001
Heart Failure	1,673	1,583	3.0%	2.8%	0.055
Hypertension	19,476	20,224	34.6%	35.5%	0.001
Osteoporosis	2,017	2,023	3.6%	3.6%	0.794
Peripheral Vascular Disease	1,769	1,770	3.1%	3.1%	0.752
Physical Disability	384	409	0.7%	0.7%	0.462
**LTC_PL**					
Alcohol Dependence	201	193	0.4%	0.3%	0.608
Asthma	5,288	5,303	9.4%	9.3%	0.657
Bronchiectasis	431	468	0.8%	0.8%	0.283
Chronic Pain	1,594	1,512	2.8%	2.7%	0.071
COPD	2,220	2,125	3.9%	3.7%	0.066
Epilepsy	484	425	0.9%	0.7%	0.033
Inflammatory Bowel Disease	654	682	1.2%	1.2%	0.57
Multiple Sclerosis	101	127	0.2%	0.2%	0.101
Neurological Organ Failure	41	40	0.1%	0.1%	0.872
Osteoarthritis	8,639	8,948	15.3%	15.7%	0.082
Parkinson's Disease	104	120	0.2%	0.2%	0.323
Pulmonary Heart Disease	677	676	1.2%	1.2%	0.817
Rheumatoid Arthritis	761	745	1.4%	1.3%	0.528
Sarcoidosis	103	109	0.2%	0.2%	0.74

	N<br>Referred to the NHS DPP but did not attend any intervention sessions	Referred to the NHS DPP and completed	%<br>Referred to the NHS DPP but did not attend any intervention sessions	Referred to the NHS DPP and completed	P value
Serious Mental Illness	635	642	1.1%	1.1%	0.999
Severe Interstitial Lung Disease	135	134	0.2%	0.2%	0.879
Age~	65.4	66.0	13.1	10.6	<0.001
Sex					
Female	30,944	31,143	54.9%	54.7%	0.391
Male	25,371	25,797	45.1%	45.3%	
Ethnicity					
Asian	4,039	3,941	7.2%	6.9%	<0.001
Black	2,822	2,859	5.0%	5.0%	
Mixed	703	718	1.2%	1.3%	
Other	1,378	1,313	2.4%	2.3%	
Unknown	3,972	3,630	7.1%	6.4%	
White	43,401	44,479	77.1%	78.1%	
IMD quintile					
1 (most deprived)	7,800	7,527	13.9%	13.2%	0.002
2	9,901	9,879	17.6%	17.3%	
3	11,784	11,815	20.9%	20.7%	
4	12,885	13,188	22.9%	23.2%	
5 (least deprived)	13,945	14,531	24.8%	25.5%	
Rural GP					
No	49,862	50,513	88.5%	88.7%	0.364
Yes	6,453	6,427	11.5%	11.3%	
IMD quintile GP					
1 (most deprived)	14,661	15,031	26.0%	26.4%	0.098
2	12,564	12,535	22.3%	22.0%	
3	11,051	11,397	19.6%	20.0%	
4	10,581	10,443	18.8%	18.3%	
5 (least deprived)	7,458	7,534	13.2%	13.2%	
QOF overall quintile GP					
1 (Highest achievement)	9,164	8,952	16.3%	15.7%	0.06
2	10,476	10,468	18.6%	18.4%	
3	11,798	12,115	21.0%	21.3%	
4	12,148	12,346	21.6%	21.7%	
5 (Lowest achievement)	12,729	13,059	22.6%	22.9%	
size quintile GP					
1 (Smallest)	10,108	10,410	17.9%	18.3%	0.465
2	11,181	11,267	19.9%	19.8%	
3	11,462	11,600	20.4%	20.4%	
4	11,825	11,754	21.0%	20.6%	
5 (Largest)	11,739	11,909	20.8%	20.9%	
FTE quintile GP					
1 (Least GPs per patient)	9,519	9,586	16.9%	16.8%	0.718
2	11,588	11,914	20.6%	20.9%	
3	12,094	12,162	21.5%	21.4%	
4	11,973	12,072	21.3%	21.2%	
5 (Most GPs per patient)	11,141	11,206	19.8%	19.7%	
Arrivals~	0.3	0.3	0.7	0.8	0.279
Admissions~	0.4	0.4	1.1	1.0	0.175
Appointments~	3.2	3.3	5.6	5.5	0.0023

~ Mean & SD reported rather than N & %. P values for categorical variables were calculated using two-sided chi-square tests and p values for continuous variables were calculated using two-sided t-tests

Supplementary table S3: Baseline characteristics of the 6-months cohort, for those referred to the NHS DPP and completed the programme (intervention group) and those who were referred to the NHS DPP but did not start the NHS DPP (control group), by sex

	Women					Men				
	N Referred to the NHS DPP but did not attend any intervention sessions	Referred to the NHS DPP and completed	% Referred to the NHS DPP but did not attend any intervention sessions	Referred to the NHS DPP and completed	P value	N Referred to the NHS DPP but did not attend any intervention sessions	Referred to the NHS DPP and completed	% Referred to the NHS DPP but did not attend any intervention sessions	Referred to the NHS DPP and completed	P value
Total	30,944	31,143	100%	100%		25,371	25,797	100%	100%	
Number of LTC_Ls										
0	15,840	16,373	51.2%	52.6%		12,978	12,006	51.2%	46.5%	
At least 1	15,104	14,770	48.8%	47.4%	0.001	12,393	13,791	48.8%	53.5%	<0.001
At least 2	6,898	6,307	22.3%	20.3%	<0.001	6,704	7,390	26.4%	28.6%	<0.001
At least 3	2,842	2,322	9.2%	7.5%	<0.001	3,048	3,312	12.0%	12.8%	0.005
At least 4	1,109	791	3.6%	2.5%	<0.001	1,300	1,343	5.1%	5.2%	0.675
Number of LTC_PLs										
0	21,266	21,699	68.7%	69.7%		19,001	18,902	74.9%	73.3%	
At least 1	9,678	9,444	31.3%	30.3%	0.01	6,370	6,895	25.1%	26.7%	<0.001
At least 2	2,776	2,684	9.0%	8.6%	0.121	1,564	1,684	6.2%	6.5%	0.092
At least 3	863	744	2.8%	2.4%	0.002	394	435	1.6%	1.7%	0.232
At least 4	236	172	0.8%	0.6%	0.002	92	103	0.4%	0.4%	0.501
Number of LTCs										
0	13,330	13,712	43.1%	44.0%		11,445	10,439	45.1%	40.5%	
At least 1	17,614	17,431	56.9%	56.0%	0.017	13,926	15,358	54.9%	59.5%	<0.001
At least 2	10,792	10,281	34.9%	33.0%	<0.001	8,898	9,823	35.1%	38.1%	<0.001
At least 3	5,867	5,288	19.0%	17.0%	<0.001	4,850	5,377	19.1%	20.8%	<0.001
At least 4	3,038	2,569	9.8%	8.2%	<0.001	2,583	2,753	10.2%	10.7%	0.069
**LTC_L**										
Atrial Fibrillation	1,761	1,423	5.7%	4.6%	<0.001	2,085	2,471	8.2%	9.6%	<0.001
Cancer	2,154	2,206	7.0%	7.1%	0.55	2,238	2,348	8.8%	9.1%	0.266
Cerebrovascular Disease	1,030	900	3.3%	2.9%	0.002	1,065	1,061	4.2%	4.1%	0.631
Chronic Kidney Disease	747	634	2.4%	2.0%	0.001	593	599	2.3%	2.3%	0.908

	Women					Men				
	N Referred to the NHS DPP but did not attend any intervention sessions	N Referred to the NHS DPP and completed	% Referred to the NHS DPP but did not attend any intervention sessions	% Referred to the NHS DPP and completed	P value	N Referred to the NHS DPP but did not attend any intervention sessions	N Referred to the NHS DPP and completed	% Referred to the NHS DPP but did not attend any intervention sessions	% Referred to the NHS DPP and completed	P value
Chronic Liver Disease	147	119	0.5%	0.4%	0.076	159	163	0.6%	0.6%	0.941
Coronary Heart Disease	2,944	2,643	9.5%	8.5%	<0.001	4,580	5,087	18.1%	19.7%	<0.001
Dementia	271	202	0.9%	0.6%	0.001	243	230	1.0%	0.9%	0.434
Depression	3,090	2,940	10.0%	9.4%	0.022	1,476	1,453	5.8%	5.6%	0.367
Frailty	532	307	1.7%	1.0%	<0.001	381	323	1.5%	1.3%	0.015
Heart Failure	717	544	2.3%	1.7%	<0.001	956	1,039	3.8%	4.0%	0.129
Hypertension	10,639	10,206	34.4%	32.8%	<0.001	8,837	10,018	34.8%	38.8%	<0.001
Osteoporosis	1,658	1,614	5.4%	5.2%	0.328	359	409	1.4%	1.6%	0.113
Peripheral Vascular Disease	719	651	2.3%	2.1%	0.048	1,050	1,119	4.1%	4.3%	0.264
Physical Disability	185	206	0.6%	0.7%	0.316	199	203	0.8%	0.8%	0.974
**LTC_PL**										
Alcohol Dependence	58	45	0.2%	0.1%	0.189	143	148	0.6%	0.6%	0.88
Asthma	3,424	3,311	11.1%	10.6%	0.082	1,864	1,992	7.3%	7.7%	0.108
Bronchiectasis	251	265	0.8%	0.9%	0.585	180	203	0.7%	0.8%	0.31
Chronic Pain	1,134	1,092	3.7%	3.5%	0.289	460	420	1.8%	1.6%	0.108
COPD	1,211	960	3.9%	3.1%	<0.001	1,009	1,165	4.0%	4.5%	0.003
Epilepsy	244	225	0.8%	0.7%	0.342	240	200	0.9%	0.8%	0.037
Inflammatory Bowel Disease	340	333	1.1%	1.1%	0.723	314	349	1.2%	1.4%	0.249
Multiple Sclerosis	74	106	0.2%	0.3%	0.019	27	21	0.1%	0.1%	0.355
Neurological Organ Failure	23	22	0.1%	0.1%	0.865	18	18	0.1%	0.1%	0.96
Osteoarthritis	5,421	5,374	17.5%	17.3%	0.388	3,218	3,574	12.7%	13.9%	<0.001
Parkinson's Disease	48	43	0.2%	0.1%	0.578	56	77	0.2%	0.3%	0.084
Pulmonary Heart Disease	362	308	1.2%	1.0%	0.029	315	368	1.2%	1.4%	0.068
Rheumatoid Arthritis	544	522	1.8%	1.7%	0.432	217	223	0.9%	0.9%	0.911
Sarcoidosis	58	58	0.2%	0.2%	0.972	45	51	0.2%	0.2%	0.595
Serious Mental Illness	361	375	1.2%	1.2%	0.666	274	267	1.1%	1.0%	0.619
Severe Interstitial Lung Disease	69	56	0.2%	0.2%	0.23	66	78	0.3%	0.3%	0.367

	Women					Men				
	N		%			N		%		
	Referred to the NHS DPP but did not attend any intervention sessions	Referred to the NHS DPP and completed	Referred to the NHS DPP but did not attend any intervention sessions	Referred to the NHS DPP and completed	P value	Referred to the NHS DPP but did not attend any intervention sessions	Referred to the NHS DPP and completed	Referred to the NHS DPP but did not attend any intervention sessions	Referred to the NHS DPP and completed	P value
Age~	66.1	65.6	13.1	10.6	<0.001	64.5	66.5	13.1	10.6	<0.001
Ethnicity										
Asian	2,195	2,353	7.1%	7.6%	<0.001	1,844	1,588	7.3%	6.2%	<0.001
Black	1,622	1,939	5.2%	6.2%		1,200	920	4.7%	3.6%	
Mixed	369	422	1.2%	1.4%		334	296	1.3%	1.1%	
Other	773	793	2.5%	2.5%		605	520	2.4%	2.0%	
Unknown	1,862	1,791	6.0%	5.8%		2,110	1,839	8.3%	7.1%	
White	24,123	23,845	78.0%	76.6%		19,278	20,634	76.0%	80.0%	
IMD quintile										
1 (most deprived)	4,346	4,209	14.0%	13.5%	0.356	3,454	3,318	13.6%	12.9%	<0.001
2	5,507	5,550	17.8%	17.8%		4,394	4,329	17.3%	16.8%	
3	6,507	6,532	21.0%	21.0%		5,277	5,283	20.8%	20.5%	
4	6,986	7,151	22.6%	23.0%		5,899	6,037	23.3%	23.4%	
5 (least deprived)	7,598	7,701	24.6%	24.7%		6,347	6,830	25.0%	26.5%	
Rural GP										
No	27,492	27,778	88.8%	89.2%	0.162	22,370	22,735	88.2%	88.1%	0.886
Yes	3,452	3,365	11.2%	10.8%		3,001	3,062	11.8%	11.9%	
IMD quintile GP										
1 (most deprived)	7,989	7,971	25.8%	25.6%	0.703	6,672	7,060	26.3%	27.4%	0.008
2	6,924	6,938	22.4%	22.3%		5,640	5,597	22.2%	21.7%	
3	6,118	6,243	19.8%	20.0%		4,933	5,154	19.4%	20.0%	
4	5,831	5,800	18.8%	18.6%		4,750	4,643	18.7%	18.0%	
5 (least deprived)	4,082	4,191	13.2%	13.5%		3,376	3,343	13.3%	13.0%	
QOF overall quintile GP										
1 (Highest achievement)	5,085	4,852	16.4%	15.6%	0.031	4,079	4,100	16.1%	15.9%	0.526
2	5,675	5,707	18.3%	18.3%		4,801	4,761	18.9%	18.5%	
3	6,489	6,630	21.0%	21.3%		5,309	5,485	20.9%	21.3%	

	Women					Men				
	N<br>Referred to the NHS DPP but did not attend any intervention sessions	Referred to the NHS DPP and completed	%<br>Referred to the NHS DPP but did not attend any intervention sessions	Referred to the NHS DPP and completed	P value	N<br>Referred to the NHS DPP but did not attend any intervention sessions	Referred to the NHS DPP and completed	%<br>Referred to the NHS DPP but did not attend any intervention sessions	Referred to the NHS DPP and completed	P value
4	6,702	6,718	21.7%	21.6%		5,446	5,628	21.5%	21.8%	
5 (Lowest achievement)	6,993	7,236	22.6%	23.2%		5,736	5,823	22.6%	22.6%	
size quintile GP										
1 (Smallest)	5,510	5,766	17.8%	18.5%	0.156	4,598	4,644	18.1%	18.0%	0.492
2	6,156	6,198	19.9%	19.9%		5,025	5,069	19.8%	19.6%	
3	6,323	6,386	20.4%	20.5%		5,139	5,214	20.3%	20.2%	
4	6,440	6,361	20.8%	20.4%		5,385	5,393	21.2%	20.9%	
5 (Largest)	6,515	6,432	21.1%	20.7%		5,224	5,477	20.6%	21.2%	
FTE quintile GP										
1 (Least GPs per patient)	5,179	5,291	16.7%	17.0%	0.452	4,340	4,295	17.1%	16.6%	0.535
2	6,416	6,592	20.7%	21.2%		5,172	5,322	20.4%	20.6%	
3	6,621	6,630	21.4%	21.3%		5,473	5,532	21.6%	21.4%	
4	6,643	6,545	21.5%	21.0%		5,330	5,527	21.0%	21.4%	
5 (Most GPs per patient)	6,085	6,085	19.7%	19.5%		5,056	5,121	19.9%	19.9%	
Arrivals~	0.3	0.3	0.7	0.7	0.07	0.3	0.3	0.7	0.8	0.7935
Admissions~	0.4	0.4	1.1	1.0	0.87	0.4	0.4	1.1	1.1	0.033
Appointments~	3.3	3.4	5.8	5.7	0.54	3.0	3.2	5.3	5.3	<0.001

~ Mean & SD reported rather than N & %. P values for categorical variables were calculated using two-sided chi-square tests and p values for continuous variables were calculated using two-sided t-tests

Supplementary table S4: Baseline characteristics of the 6-months cohort, for those referred to the NHS DPP and completed the programme (intervention group) and those who were referred to the NHS DPP but did not start the NHS DPP (control group), by age

	<70 years					70 years and over				
	N<br>Referred to the NHS DPP but did not attend any intervention sessions	Referred to the NHS DPP and completed	%<br>Referred to the NHS DPP but did not attend any intervention sessions	Referred to the NHS DPP and completed	P value	N<br>Referred to the NHS DPP but did not attend any intervention sessions	Referred to the NHS DPP and completed	%<br>Referred to the NHS DPP but did not attend any intervention sessions	Referred to the NHS DPP and completed	P value
Total	32,679	33,453	100%	100%		23,636	23,487	100%	100%	
Number of LTC_Ls										
0	20,378	19,766	62.4%	59.1%		8,440	8,613	35.7%	36.7%	
At least 1	12,301	13,687	37.6%	40.9%	<0.001	15,196	14,874	64.3%	63.3%	0.03
At least 2	4,794	5,531	14.7%	16.5%	<0.001	8,808	8,166	37.3%	34.8%	<0.001
At least 3	1,590	1,898	4.9%	5.7%	<0.001	4,300	3,736	18.2%	15.9%	<0.001
At least 4	521	581	1.6%	1.7%	0.152	1,888	1,553	8.0%	6.6%	<0.001
Number of LTC_PLs										
0	25,187	25,258	77.1%	75.5%		15,080	15,343	63.8%	65.3%	
At least 1	7,492	8,195	22.9%	24.5%	<0.001	8,556	8,144	36.2%	34.7%	0.001
At least 2	1,872	2,084	5.7%	6.2%	0.007	2,468	2,284	10.4%	9.7%	0.01
At least 3	503	548	1.5%	1.6%	0.309	754	631	3.2%	2.7%	0.001
At least 4	119	137	0.4%	0.4%	0.347	209	138	0.9%	0.6%	<0.001
Number of LTCs										
0	17,694	17,014	54.1%	50.9%		7,081	7,137	30.0%	30.4%	
At least 1	14,985	16,439	45.9%	49.1%	<0.001	16,555	16,350	70.0%	69.6%	0.311
At least 2	7,905	8,912	24.2%	26.6%	<0.001	11,785	11,192	49.9%	47.7%	<0.001
At least 3	3,668	4,298	11.2%	12.8%	<0.001	7,049	6,367	29.8%	27.1%	<0.001
At least 4	1,656	1,883	5.1%	5.6%	0.001	3,965	3,439	16.8%	14.6%	<0.001
**LTC_L**										
Atrial Fibrillation	876	1,157	2.7%	3.5%	<0.001	2,970	2,737	12.6%	11.7%	0.002
Cancer	1,603	1,947	4.9%	5.8%	<0.001	2,789	2,607	11.8%	11.1%	0.017
Cerebrovascular Disease	706	802	2.2%	2.4%	0.041	1,389	1,159	5.9%	4.9%	<0.001
Chronic Kidney Disease	254	318	0.8%	1.0%	0.016	1,086	915	4.6%	3.9%	<0.001
Chronic Liver Disease	192	180	0.6%	0.5%	0.395	114	102	0.5%	0.4%	0.44

	<70 years					70 years and over				
	N Referred to the NHS DPP but did not attend any intervention sessions	Referred to the NHS DPP and completed	% Referred to the NHS DPP but did not attend any intervention sessions	Referred to the NHS DPP and completed	P value	N Referred to the NHS DPP but did not attend any intervention sessions	Referred to the NHS DPP and completed	% Referred to the NHS DPP but did not attend any intervention sessions	Referred to the NHS DPP and completed	P value
Coronary Heart Disease	2,844	3,111	8.7%	9.3%	0.007	4,680	4,619	19.8%	19.7%	0.715
Dementia	85	133	0.3%	0.4%	0.002	429	299	1.8%	1.3%	<0.001
Depression	3,284	3,223	10.0%	9.6%	0.073	1,282	1,170	5.4%	5.0%	0.031
Frailty	128	142	0.4%	0.4%	0.509	785	488	3.3%	2.1%	<0.001
Heart Failure	523	570	1.6%	1.7%	0.297	1,150	1,013	4.9%	4.3%	0.004
Hypertension	7,621	8,715	23.3%	26.1%	<0.001	11,855	11,509	50.2%	49.0%	0.012
Osteoporosis	509	699	1.6%	2.1%	<0.001	1,508	1,324	6.4%	5.6%	0.001
Peripheral Vascular Disease	623	738	1.9%	2.2%	0.007	1,146	1,032	4.8%	4.4%	0.019
Physical Disability	194	231	0.6%	0.7%	0.119	190	178	0.8%	0.8%	0.571
**LTC_PL**										
Alcohol Dependence	159	137	0.5%	0.4%	0.138	42	56	0.2%	0.2%	0.148
Asthma	2,993	3,038	9.2%	9.1%	0.73	2,295	2,265	9.7%	9.6%	0.808
Bronchiectasis	137	163	0.4%	0.5%	0.193	294	305	1.2%	1.3%	0.596
Chronic Pain	844	894	2.6%	2.7%	0.471	750	618	3.2%	2.6%	<0.001
COPD	825	862	2.5%	2.6%	0.67	1,395	1,263	5.9%	5.4%	0.014
Epilepsy	288	276	0.9%	0.8%	0.431	196	149	0.8%	0.6%	0.013
Inflammatory Bowel Disease	369	399	1.1%	1.2%	0.446	285	283	1.2%	1.2%	0.993
Multiple Sclerosis	63	102	0.2%	0.3%	0.004	38	25	0.2%	0.1%	0.107
Neurological Organ Failure	12	14	0.0%	0.0%	0.739	29	26	0.1%	0.1%	0.703
Osteoarthritis	3,160	3,780	9.7%	11.3%	<0.001	5,479	5,168	23.2%	22.0%	0.002
Parkinson's Disease	23	48	0.1%	0.1%	0.004	81	72	0.3%	0.3%	0.49
Pulmonary Heart Disease	245	292	0.7%	0.9%	0.078	432	384	1.8%	1.6%	0.109
Rheumatoid Arthritis	304	370	0.9%	1.1%	0.024	457	375	1.9%	1.6%	0.005
Sarcoidosis	63	77	0.2%	0.2%	0.296	40	32	0.2%	0.1%	0.359
Serious Mental Illness	495	515	1.5%	1.5%	0.795	140	127	0.6%	0.5%	0.456
Severe Interstitial Lung Disease	38	42	0.1%	0.1%	0.732	97	92	0.4%	0.4%	0.748
Age~	56.6	59.3	9.4	8.3	<0.001	77.5	75.5	5.6	4.5	<0.001
Sex										

	<70 years					70 years and over				
	N Referred to the NHS DPP but did not attend any interventio n sessions	Referred to the NHS DPP and completed	% Referred to the NHS DPP but did not attend any interventio n sessions	Referred to the NHS DPP and completed	P value	N Referred to the NHS DPP but did not attend any interventio n sessions	Referred to the NHS DPP and completed	% Referred to the NHS DPP but did not attend any interventio n sessions	Referred to the NHS DPP and completed	P value
Female	17,179	18,805	52.6%	56.2%	<0.001	13,765	12,338	58.2%	52.5%	<0.001
Male	15,500	14,648	47.4%	43.8%		9,871	11,149	41.8%	47.5%	
Ethnicity										
Asian	3,384	3,372	10.4%	10.1%	<0.001	655	569	2.8%	2.4%	0.042
Black	2,377	2,404	7.3%	7.2%		445	455	1.9%	1.9%	
Mixed	552	576	1.7%	1.7%		151	142	0.6%	0.6%	
Other	1,061	1,050	3.2%	3.1%		317	263	1.3%	1.1%	
Unknown	2,869	2,560	8.8%	7.7%		1,103	1,070	4.7%	4.6%	
White	22,436	23,491	68.7%	70.2%		20,965	20,988	88.7%	89.4%	
IMD quintile										
1 (most deprived)	5,599	5,360	17.1%	16.0%	<0.001	2,201	2,167	9.3%	9.2%	0.95
2	6,397	6,396	19.6%	19.1%		3,504	3,483	14.8%	14.8%	
3	6,856	6,971	21.0%	20.8%		4,928	4,844	20.8%	20.6%	
4	6,901	7,237	21.1%	21.6%		5,984	5,951	25.3%	25.3%	
5 (least deprived)	6,926	7,489	21.2%	22.4%		7,019	7,042	29.7%	30.0%	
Rural GP										
No	29,529	30,138	90.4%	90.1%	0.242	20,333	20,375	86.0%	86.8%	0.022
Yes	3,150	3,315	9.6%	9.9%		3,303	3,112	14.0%	13.2%	
IMD quintile GP										
1 (most deprived)	7,254	7,769	22.2%	23.2%	0.011	7,407	7,262	31.3%	30.9%	0.054
2	6,815	6,982	20.9%	20.9%		5,749	5,553	24.3%	23.6%	
3	6,633	6,787	20.3%	20.3%		4,418	4,610	18.7%	19.6%	
4	6,680	6,591	20.4%	19.7%		3,901	3,852	16.5%	16.4%	
5 (least deprived)	5,297	5,324	16.2%	15.9%		2,161	2,210	9.1%	9.4%	
QOF overall quintile GP										
1 (Highest achievement)	5,605	5,549	17.2%	16.6%	0.092	3,559	3,403	15.1%	14.5%	0.26
2	6,369	6,367	19.5%	19.0%		4,107	4,101	17.4%	17.5%	
3	6,970	7,259	21.3%	21.7%		4,828	4,856	20.4%	20.7%	

	<70 years					70 years and over				
	N Referred to the NHS DPP but did not attend any interventio n sessions	Referred to the NHS DPP and completed	% Referred to the NHS DPP but did not attend any interventio n sessions	Referred to the NHS DPP and completed	P value	N Referred to the NHS DPP but did not attend any interventio n sessions	Referred to the NHS DPP and completed	% Referred to the NHS DPP but did not attend any interventio n sessions	Referred to the NHS DPP and completed	P value
4	6,655	6,970	20.4%	20.8%		5,493	5,376	23.2%	22.9%	
5 (Lowest achievement)	7,080	7,308	21.7%	21.8%		5,649	5,751	23.9%	24.5%	
size quintile GP										
1 (Smallest)	6,219	6,543	19.0%	19.6%	0.38	3,889	3,867	16.5%	16.5%	0.496
2	6,563	6,719	20.1%	20.1%		4,618	4,548	19.5%	19.4%	
3	6,566	6,760	20.1%	20.2%		4,896	4,840	20.7%	20.6%	
4	6,615	6,667	20.2%	19.9%		5,210	5,087	22.0%	21.7%	
5 (Largest)	6,716	6,764	20.6%	20.2%		5,023	5,145	21.3%	21.9%	
FTE quintile GP										
1 (Least GPs per patient)	6,042	6,196	18.5%	18.5%	0.073	3,477	3,390	14.7%	14.4%	0.321
2	6,769	7,172	20.7%	21.4%		4,819	4,742	20.4%	20.2%	
3	6,849	7,014	21.0%	21.0%		5,245	5,148	22.2%	21.9%	
4	6,739	6,879	20.6%	20.6%		5,234	5,193	22.1%	22.1%	
5 (Most GPs per patient)	6,280	6,192	19.2%	18.5%		4,861	5,014	20.6%	21.3%	
Arrivals~	0.3	0.3	0.7	0.8	0.085	0.3	0.3	0.7	0.8	0.7089
Admissions~	0.3	0.3	0.9	1.0	0.019	0.5	0.5	1.2	1.1	0.9275
Appointments~	2.6	2.9	5.3	5.4	<0.001	4.0	3.8	5.9	5.6	0.0199

~ Mean & SD reported rather than N & %. P values for categorical variables were calculated using two-sided chi-square tests and p values for continuous variables were calculated using two-sided t-tests

Supplementary table S5: Number individuals living with MLTC at the start of the follow-up period and number who developed MLTC during follow-up

	6 months			12 months			18 months			24 months		
	Control	Intervention	P value*	Control	Intervention	P value*	Control	Intervention	P value*	Control	Intervention	P value*
N	56,315	56,940		39,773	38,140		26,600	22,497		14,594	11,821	
Living with MLTC	19,690	20,104		13,681	13,347		8,945	7,767		4,795	4,055	
Not living with MLTC	36,625	36,836		26,092	24,793		17,655	14,730		9,799	7,766	
% Living with MLTC	35.0%	35.3%	0.226	34.4%	35.0%	0.080	33.6%	34.5%	0.037	32.9%	34.3%	0.013
MLTC at follow-up	1,671	1,039		2,291	1,557		2,278	1,464		1,640	1,042	
No MLTC at follow-up	34,954	35,797		23,801	23,236		15,377	13,266		8,159	6,724	
% MLTC at follow-up	4.6%	2.8%	<0.001	8.8%	6.3%	<0.001	12.9%	9.9%	<0.001	16.7%	13.4%	<0.001

*P value for the percentage of individuals living with MLTC at baseline calculated using a two-sided chi-square test and p value for individuals who developed MLTC at follow-up calculated using logistic regression

Supplementary table S6: Baseline characteristics of the 6-months cohort for those referred to the NHS DPP and completed the programme (intervention group) and those who were referred to the NHS DPP but did not start the NHS DPP (control group), by MLTC status

	MLTC					Not MLTC				
	N Referred to the NHS DPP but did not attend any intervention sessions	Referred to the NHS DPP and completed	% Referred to the NHS DPP but did not attend any intervention sessions	Referred to the NHS DPP and completed	P value	N Referred to the NHS DPP but did not attend any intervention sessions	Referred to the NHS DPP and completed	% Referred to the NHS DPP but did not attend any intervention sessions	Referred to the NHS DPP and completed	P value
Total	19,690	20,104	100%	100%		36,625	36,836	100%	100%	
**LTC_L**										
Atrial Fibrillation	3,581	3,623	18.2%	18.0%	0.668	265	271	0.7%	0.7%	0.847
Cancer	3,523	3,547	17.9%	17.6%	0.516	869	1,007	2.4%	2.7%	0.002
Cerebrovascular Disease	1,920	1,754	9.8%	8.7%	<0.001	175	207	0.5%	0.6%	0.113
Chronic Kidney Disease	1,292	1,186	6.6%	5.9%	0.006	48	47	0.1%	0.1%	0.896
Chronic Liver Disease	262	236	1.3%	1.2%	0.16	44	46	0.1%	0.1%	0.854
Coronary Heart Disease	6,700	6,878	34.0%	34.2%	0.698	824	852	2.2%	2.3%	0.567
Dementia	421	370	2.1%	1.8%	0.033	93	62	0.3%	0.2%	0.011
Depression	3,383	3,207	17.2%	16.0%	<0.001	1,183	1,186	3.2%	3.2%	0.937
Frailty	895	605	4.5%	3.0%	<0.001	18	25	0.0%	0.1%	0.294
Heart Failure	1,647	1,547	8.4%	7.7%	0.014	26	36	0.1%	0.1%	0.212
Hypertension	14,838	15,154	75.4%	75.4%	0.963	4,638	5,070	12.7%	13.8%	<0.001
Osteoporosis	1,776	1,694	9.0%	8.4%	0.036	241	329	0.7%	0.9%	<0.001
Peripheral Vascular Disease	1,645	1,653	8.4%	8.2%	0.632	124	117	0.3%	0.3%	0.62
Physical Disability	356	374	1.8%	1.9%	0.697	28	35	0.1%	0.1%	0.390
**LTC_PL**										
Alcohol Dependence	180	184	0.9%	0.9%	0.991	21	9	0.1%	0.0%	0.027
Asthma	4,312	4,364	21.9%	21.7%	0.642	976	939	2.7%	2.5%	0.325
Bronchiectasis	406	435	2.1%	2.2%	0.48	25	33	0.1%	0.1%	0.303
Chronic Pain	1,484	1,420	7.5%	7.1%	0.069	110	92	0.3%	0.2%	0.19
COPD	2,045	1,943	10.4%	9.7%	0.017	175	182	0.5%	0.5%	0.751
Epilepsy	402	358	2.0%	1.8%	0.057	82	67	0.2%	0.2%	0.206
Inflammatory Bowel Disease	508	528	2.6%	2.6%	0.772	146	154	0.4%	0.4%	0.68
Multiple Sclerosis	83	90	0.4%	0.4%	0.692	18	37	0.0%	0.1%	0.011
Neurological Organ Failure	41	40	0.2%	0.2%	0.838	0	0	0.0%	0.0%	n/a

	MLTC					Not MLTC				
	N<br>Referred to the NHS DPP but did not attend any intervention sessions	Referred to the NHS DPP and completed	%<br>Referred to the NHS DPP but did not attend any intervention sessions	Referred to the NHS DPP and completed	P value	N<br>Referred to the NHS DPP but did not attend any intervention sessions	Referred to the NHS DPP and completed	%<br>Referred to the NHS DPP but did not attend any intervention sessions	Referred to the NHS DPP and completed	P value
Osteoarthritis	7,229	7,405	36.7%	36.8%	0.805	1,410	1,543	3.8%	4.2%	0.019
Parkinson's Disease	88	96	0.4%	0.5%	0.653	16	24	0.0%	0.1%	0.212
Pulmonary Heart Disease	609	612	3.1%	3.0%	0.778	68	64	0.2%	0.2%	0.703
Rheumatoid Arthritis	761	745	3.9%	3.7%	0.405	0	0	0.0%	0.0%	n/a
Sarcoidosis	90	93	0.5%	0.5%	0.935	13	16	0.0%	0.0%	0.588
Serious Mental Illness	428	417	2.2%	2.1%	0.491	207	225	0.6%	0.6%	0.419
Severe Interstitial Lung Disease	128	124	0.7%	0.6%	0.676	7	10	0.0%	0.0%	0.474
Age~	70.8	69.7	11.4	9.2	<0.001	62.5	64.0	13.0	10.8	<0.001
Sex										
Female	10,792	10,281	54.8%	51.1%	<0.001	20,152	20,862	55.0%	56.6%	<0.001
Male	8,898	9,823	45.2%	48.9%		16,473	15,974	45.0%	43.4%	
Ethnicity										
Asian	860	947	4.4%	4.7%	0.002	3,179	2,994	8.7%	8.1%	<0.001
Black	566	690	2.9%	3.4%		2,256	2,169	6.2%	5.9%	
Mixed	127	160	0.6%	0.8%		576	558	1.6%	1.5%	
Other	263	287	1.3%	1.4%		1,115	1,026	3.0%	2.8%	
Unknown	194	174	1.0%	0.9%		3,778	3,456	10.3%	9.4%	
White	17,680	17,846	89.8%	88.8%		25,721	26,633	70.2%	72.3%	
IMD quintile										
1 (most deprived)	2,742	2,600	13.9%	12.9%	0.051	5,058	4,927	13.8%	13.4%	0.011
2	3,397	3,490	17.3%	17.4%		6,504	6,389	17.8%	17.3%	
3	4,081	4,166	20.7%	20.7%		7,703	7,649	21.0%	20.8%	
4	4,529	4,758	23.0%	23.7%		8,356	8,430	22.8%	22.9%	
5 (least deprived)	4,941	5,090	25.1%	25.3%		9,004	9,441	24.6%	25.6%	
Rural GP										
No	17,296	17,726	87.8%	88.2%	0.311	32,566	32,787	88.9%	89.0%	0.695
Yes	2,394	2,378	12.2%	11.8%		4,059	4,049	11.1%	11.0%	
IMD quintile GP										
1 (most deprived)	5,317	5,367	27.0%	26.7%	0.012	9,344	9,664	25.5%	26.2%	0.177

	MLTC					Not MLTC				
	N<br>Referred to the NHS DPP but did not attend any intervention sessions	Referred to the NHS DPP and completed	%<br>Referred to the NHS DPP but did not attend any intervention sessions	Referred to the NHS DPP and completed	P value	N<br>Referred to the NHS DPP but did not attend any intervention sessions	Referred to the NHS DPP and completed	%<br>Referred to the NHS DPP but did not attend any intervention sessions	Referred to the NHS DPP and completed	P value
2	4,417	4,427	22.4%	22.0%		8,147	8,108	22.2%	22.0%	
3	3,774	4,049	19.2%	20.1%		7,277	7,348	19.9%	19.9%	
4	3,797	3,702	19.3%	18.4%		6,784	6,741	18.5%	18.3%	
5 (least deprived)	2,385	2,559	12.1%	12.7%		5,073	4,975	13.9%	13.5%	
QOF overall quintile GP										
1 (Highest achievement)	3,080	3,057	15.6%	15.2%	0.64	6,084	5,895	16.6%	16.0%	0.141
2	3,573	3,598	18.1%	17.9%		6,903	6,870	18.8%	18.7%	
3	4,065	4,174	20.6%	20.8%		7,733	7,941	21.1%	21.6%	
4	4,368	4,486	22.2%	22.3%		7,780	7,860	21.2%	21.3%	
5 (Lowest achievement)	4,604	4,789	23.4%	23.8%		8,125	8,270	22.2%	22.5%	
size quintile GP										
1 (Smallest)	3,334	3,508	16.9%	17.4%	0.591	6,774	6,902	18.5%	18.7%	0.685
2	3,812	3,892	19.4%	19.4%		7,369	7,375	20.1%	20.0%	
3	4,129	4,146	21.0%	20.6%		7,333	7,454	20.0%	20.2%	
4	4,213	4,233	21.4%	21.1%		7,612	7,521	20.8%	20.4%	
5 (Largest)	4,202	4,325	21.3%	21.5%		7,537	7,584	20.6%	20.6%	
FTE quintile GP										
1 (Least GPs per patient)	3,054	3,115	15.5%	15.5%	0.417	6,465	6,471	17.7%	17.6%	0.618
2	3,960	4,142	20.1%	20.6%		7,628	7,772	20.8%	21.1%	
3	4,272	4,421	21.7%	22.0%		7,822	7,741	21.4%	21.0%	
4	4,354	4,302	22.1%	21.4%		7,619	7,770	20.8%	21.1%	
5 (Most GPs per patient)	4,050	4,124	20.6%	20.5%		7,091	7,082	19.4%	19.2%	
Arrivals~	0.4	0.4	0.9	1.0	0.96	0.2	0.2	0.6	0.6	0.046
Admissions~	0.7	0.7	1.5	1.4	0.5955	0.2	0.2	0.7	0.6	0.2089
Appointments~	5.5	5.6	7.3	7.2	0.2607	1.9	2.0	3.9	3.8	0.001

~ Mean & SD reported rather than N & %. P values for categorical variables were calculated using two-sided chi-square tests and p values for continuous variables were calculated using two-sided t-tests

Supplementary table S7: Number of new conditions of type 2 diabetes, LTCs considered aetiologically linked to diet, physical activity and body weight (LTC-L) and LTCs possibly linked to diet, physical activity and body weight (LTC-PL) at 6,12,18 and 24 months follow-up

	6 months		12 months		18 months		24 months	
	Control	Intervention	Control	Intervention	Control	Intervention	Control	Intervention
Total	56,315	56,940	39,773	38,140	26,600	22,497	14,594	11,821
Type 2 diabetes	816	394	1,419	703	1,473	686	1,265	562
%	1.4%	0.7%	3.6%	1.8%	5.5%	3.0%	8.7%	4.8%
0 LTC-L	52,664	54,497	35,054	34,774	22,230	19,562	11,599	9,820
%	93.5%	95.7%	88.1%	91.2%	83.6%	87.0%	79.5%	83.1%
1 LTC-L	2,596	2011	3,144	2,544	2,859	2,114	1,901	1,366
%	4.6%	3.5%	7.9%	6.7%	10.7%	9.4%	13.0%	11.6%
2 LTC-L	729	332	1,022	605	976	579	689	435
%	1.3%	0.6%	2.6%	1.6%	3.7%	2.6%	4.7%	3.7%
3 LTC-L	234	78	388	163	333	163	259	121
%	0.4%	0.1%	1.0%	0.4%	1.3%	0.7%	1.8%	1.0%
4 (or more) LTC-L	92	22	165	54	202	79	146	79
%	0.2%	0.0%	0.4%	0.1%	0.8%	0.4%	1.0%	0.7%
0 LTC-PL	54,808	56,019	37,746	36,768	24,584	21,209	13,189	10,893
%	97.3%	98.4%	94.9%	96.4%	92.4%	94.3%	90.4%	92.1%
1 LTC-PL	1,301	806	1,699	1,178	1,661	1,082	1,149	762
%	2.3%	1.4%	4.3%	3.1%	6.2%	4.8%	7.9%	6.4%
2 LTC-PL	176	94	284	157	295	167	204	130
%	0.3%	0.2%	0.7%	0.4%	1.1%	0.7%	1.4%	1.1%
3 or more LTC-PL	30	21	44	37	60	39	52	36
%	0.1%	0.0%	0.1%	0.1%	0.2%	0.2%	0.4%	0.3%

Supplementary table S8: Unadjusted and adjusted estimates of incidence of type 2 diabetes, LTCs considered aetiologically linked to diet, physical activity and body weight (LTC-L) and LTCs possibly linked to diet, physical activity and body weight (LTC-PL) at 6,12,18 and 24 months follow-up

			Control	Intervention	Odds ratio / rate ratio Unadjusted	Odds ratio / rate ratio Adjusted
Type 2 diabetes	6 months	Total	56,315	56,940		
		New diagnoses	816	394		
		%	1.4%	0.7%	0.47 (0.42-0.53) <0.001	0.48 (0.43-0.54) <0.001
	12 months	Total	39,773	38,140		
		New diagnoses	1,419	703		
		%	3.6%	1.8%	0.51 (0.46-0.56) <0.001	0.51 (0.47-0.56) <0.001
	18 months	Total	26,600	22,497		
		New diagnoses	1,473	686		
		%	5.5%	3.0%	0.54 (0.49-0.59) <0.001	0.53 (0.48-0.58) <0.001
	24 months	Total	14,594	11,821		
		New diagnoses	1,265	562		
		%	8.7%	4.8%	0.53 (0.47-0.58) <0.001	0.53 (0.48-0.59) <0.001
Aetiologically linked	6 months	Total	56,315	56,940		
		New diagnoses	5,151	2,999		
		%	9.1%	5.3%	0.58 (0.54-0.61) <0.001	0.59 (0.56-0.63) <0.001
	12 months	Total	39,773	38,140		
		New diagnoses	7,085	4,474		
		%	17.8%	11.7%	0.66 (0.63-0.69) <0.001	0.69 (0.65-0.72) <0.001
	18 months	Total	26,600	22,497		
		New diagnoses	6,686	4,106		
		%	25.1%	18.3%	0.73 (0.69-0.76) <0.001	0.75 (0.72-0.79) <0.001
	24 months	Total	14,594	11,821		
		New diagnoses	4,705	2,952		
		%	32.2%	25.0%	0.77 (0.73-0.82) <0.001	0.79 (0.74-0.84) <0.001
Possibly linked	6 months	Total	56,315	56,940		
		New diagnoses	1,745	1,059		
		%	3.1%	1.9%	0.6 (0.55-0.65) <0.001	0.6 (0.55-0.65) <0.001
	12 months	Total	39,773	38,140		
		New diagnoses	2,407	1,611		
		%	6.1%	4.2%	0.7 (0.65-0.75) <0.001	0.7 (0.65-0.75) <0.001
	18 months	Total	26,600	22,497		
		New diagnoses	2,440	1,540		
		%	9.2%	6.8%	0.75 (0.69-0.8) <0.001	0.75 (0.7-0.81) <0.001
	24 months	Total	14,594	11,821		
		New diagnoses	1,722	1,136		
		%	11.8%	9.6%	0.81 (0.75-0.89) <0.001	0.8 (0.74-0.88) <0.001

Data are presented as odds ratios (diabetes) and rate ratios (LTC-L and LTC-PL) with 95% confidence intervals. P values are two-sided and are determined by logistic regression (diabetes) and negative binomial regression (LTC-L and LTC-PL), with no adjustments for multiple tests.

Supplementary table S9: Percentage of participants acquiring new conditions at 6, 12, 18 and 24 follow-up periods

	Percentage								Unadjusted OR (95% CI)			
	6 months		12 months		18 months		24 months		6 months	12 months	18 months	24 months
	Control	Inter-vention	Control	Inter-vention	Control	Inter-vention	Control	Inter-vention				
**LTC_L**												
Dementia	0.3%	≤0.1%	0.6%	0.3%	1.0%	0.4%	1.4%	0.6%	0.37 [0.28 - 0.48]	0.42 [0.33 - 0.53]	0.45 [0.36 - 0.57]	0.42 [0.32 - 0.55]
Frailty	1.3%	0.5%	2.5%	1.2%	3.0%	1.6%	3.3%	1.8%	0.40 [0.35 - 0.46]	0.46 [0.41 - 0.52]	0.53 [0.47 - 0.60]	0.56 [0.47 - 0.65]
Heart Failure	0.5%	0.2%	0.9%	0.4%	1.3%	0.8%	1.7%	1.1%	0.41 [0.33 - 0.52]	0.46 [0.38 - 0.56]	0.58 [0.48 - 0.69]	0.63 [0.51 - 0.77]
Physical Disability	≤0.1%	≤0.1%	0.3%	0.2%	0.4%	0.2%	0.5%	0.3%	0.67 [0.45 - 1.00]	0.62 [0.45 - 0.85]	0.60 [0.43 - 0.83]	0.68 [0.46 - 1.01]
Cerebrovascular Disease	0.5%	0.3%	1.0%	0.7%	1.5%	1.0%	1.9%	1.4%	0.49 [0.40 - 0.60]	0.64 [0.54 - 0.75]	0.67 [0.57 - 0.79]	0.72 [0.59 - 0.87]
Osteoporosis	0.5%	0.3%	1.0%	0.6%	1.4%	1.0%	1.9%	1.3%	0.62 [0.51 - 0.75]	0.61 [0.52 - 0.71]	0.68 [0.57 - 0.80]	0.72 [0.59 - 0.88]
Atrial Fibrillation	0.6%	0.4%	1.3%	0.8%	1.9%	1.4%	2.7%	2.0%	0.63 [0.53 - 0.74]	0.62 [0.54 - 0.71]	0.71 [0.61 - 0.82]	0.74 [0.63 - 0.86]
Chronic Liver Disease	≤0.1%	≤0.1%	0.2%	≤0.1%	0.2%	0.2%	0.3%	0.2%	0.37 [0.22 - 0.62]	0.67 [0.46 - 0.99]	0.68 [0.46 - 1.01]	0.76 [0.48 - 1.21]
Chronic Kidney Disease	0.4%	0.2%	0.8%	0.5%	1.0%	0.8%	1.5%	1.1%	0.55 [0.44 - 0.68]	0.71 [0.59 - 0.84]	0.79 [0.65 - 0.95]	0.76 [0.61 - 0.94]
Depression	0.6%	0.5%	1.3%	1.0%	1.9%	1.6%	2.5%	1.9%	0.75 [0.64 - 0.88]	0.79 [0.69 - 0.90]	0.81 [0.70 - 0.92]	0.78 [0.66 - 0.92]
Peripheral Vascular Disease	0.3%	0.2%	0.7%	0.5%	1.1%	0.7%	1.3%	1.1%	0.54 [0.43 - 0.69]	0.65 [0.54 - 0.78]	0.63 [0.52 - 0.77]	0.83 [0.66 - 1.04]
Cancer	1.0%	0.6%	1.9%	1.4%	2.7%	2.2%	3.5%	3.1%	0.53 [0.46 - 0.60]	0.74 [0.66 - 0.83]	0.81 [0.72 - 0.91]	0.87 [0.76 - 1.00]
Hypertension	2.0%	1.4%	3.8%	2.9%	5.5%	4.7%	7.1%	6.4%	0.72 [0.65 - 0.78]	0.78 [0.72 - 0.84]	0.84 [0.78 - 0.92]	0.89 [0.81 - 0.98]
Coronary Heart Disease	0.8%	0.5%	1.5%	1.1%	2.2%	1.8%	2.6%	2.5%	0.61 [0.53 - 0.71]	0.72 [0.63 - 0.81]	0.81 [0.71 - 0.92]	0.96 [0.82 - 1.12]
**LTC_PL**												
Sarcoidosis	≤0.1%	≤0.1%	≤0.1%	≤0.1%	≤0.1%	≤0.1%	≤0.1%	≤0.1%	0.79 [0.21 - 2.95]	0.52 [0.18 - 1.53]	0.54 [0.19 - 1.55]	0.37 [0.10 - 1.35]
Epilepsy	≤0.1%	≤0.1%	≤0.1%	≤0.1%	≤0.1%	≤0.1%	0.2%	≤0.1%	0.46 [0.24 - 0.89]	0.49 [0.29 - 0.82]	0.61 [0.35 - 1.04]	0.41 [0.21 - 0.79]
Alcohol Dependence	≤0.1%	≤0.1%	≤0.1%	≤0.1%	≤0.1%	≤0.1%	≤0.1%	≤0.1%	0.18 [0.07 - 0.48]	0.37 [0.19 - 0.74]	0.34 [0.16 - 0.72]	0.52 [0.23 - 1.19]
Pulmonary Heart Disease	0.2%	≤0.1%	0.3%	≤0.1%	0.5%	0.3%	0.6%	0.3%	0.42 [0.29 - 0.62]	0.43 [0.31 - 0.59]	0.58 [0.43 - 0.79]	0.52 [0.35 - 0.77]
Multiple Sclerosis	≤0.1%	≤0.1%	≤0.1%	≤0.1%	≤0.1%	≤0.1%	≤0.1%	≤0.1%	0.59 [0.14 - 2.48]	1.04 [0.34 - 3.23]	0.79 [0.22 - 2.79]	0.62 [0.11 - 3.37]
COPD	0.5%	0.2%	1.0%	0.6%	1.6%	0.9%	2.0%	1.3%	0.44 [0.36 - 0.54]	0.54 [0.45 - 0.63]	0.57 [0.48 - 0.67]	0.62 [0.51 - 0.75]
Neurological Organ Failure	≤0.1%	≤0.1%	≤0.1%	≤0.1%	≤0.1%	≤0.1%	≤0.1%	≤0.1%	0.16 [0.06 - 0.45]	0.34 [0.16 - 0.71]	0.45 [0.22 - 0.90]	0.65 [0.31 - 1.34]

	Percentage								Unadjusted OR (95% CI)			
	6 months		12 months		18 months		24 months		6 months	12 months	18 months	24 months
	Control	Inter-vention	Control	Inter-vention	Control	Inter-vention	Control	Inter-vention				
Chronic Pain	0.3%	0.2%	0.6%	0.4%	0.8%	0.6%	1.1%	0.8%	0.63 [0.49 - 0.82]	0.76 [0.62 - 0.93]	0.76 [0.62 - 0.94]	0.74 [0.58 - 0.96]
Serious Mental Illness	≤0.1%	≤0.1%	≤0.1%	≤0.1%	0.2%	≤0.1%	0.2%	0.2%	0.47 [0.27 - 0.82]	0.62 [0.40 - 0.97]	0.75 [0.47 - 1.21]	0.78 [0.44 - 1.39]
Inflammatory Bowel Disease	≤0.1%	≤0.1%	≤0.1%	≤0.1%	0.2%	≤0.1%	0.2%	0.2%	0.80 [0.48 - 1.35]	0.71 [0.45 - 1.13]	0.58 [0.36 - 0.93]	0.78 [0.45 - 1.32]
Parkinsons Disease	≤0.1%	≤0.1%	≤0.1%	≤0.1%	≤0.1%	≤0.1%	0.2%	0.2%	0.84 [0.44 - 1.60]	0.87 [0.51 - 1.48]	1.04 [0.64 - 1.71]	0.79 [0.44 - 1.43]
Rheumatoid Arthritis	≤0.1%	≤0.1%	0.2%	0.2%	0.4%	0.3%	0.5%	0.4%	0.60 [0.41 - 0.89]	0.71 [0.51 - 0.97]	0.85 [0.63 - 1.16]	0.81 [0.56 - 1.16]
Bronchiectasis	≤0.1%	≤0.1%	0.2%	0.2%	0.4%	0.3%	0.4%	0.4%	0.88 [0.60 - 1.29]	0.88 [0.64 - 1.21]	0.92 [0.68 - 1.25]	0.87 [0.60 - 1.27]
Asthma	0.4%	0.3%	0.9%	0.7%	1.3%	1.0%	1.7%	1.5%	0.71 [0.59 - 0.86]	0.79 [0.67 - 0.93]	0.77 [0.66 - 0.91]	0.88 [0.72 - 1.07]
Osteoarthritis	1.1%	0.7%	2.1%	1.6%	3.1%	2.6%	4.0%	3.9%	0.65 [0.57 - 0.74]	0.76 [0.69 - 0.85]	0.84 [0.75 - 0.93]	0.98 [0.87 - 1.12]
Severe Interstitial Lung Disease	≤0.1%	≤0.1%	≤0.1%	≤0.1%	0.2%	≤0.1%	0.2%	0.2%	0.62 [0.37 - 1.04]	0.92 [0.59 - 1.43]	0.73 [0.45 - 1.17]	1.00 [0.60 - 1.68]

Data are presented as odds ratios with 95% confidence intervals. N (intervention and control) = 56,940 and 56,315 at 6 months, 38,140 and 39,773 at 12 months, 22,497 and 26,600 at 18 months and 11,821 and 14,594 at 24 months

Supplementary table S10: Percentage of participants acquiring new conditions at 6, 12, 18 and 24 follow-up periods split by sex

Women

	**Percentage**								**Unadjusted OR (95% CI)**			
	6 months		12 months		18 months		24 months		6 months	12 months	18 months	24 months
	Control	Inter-vention	Control	Inter-vention	Control	Inter-vention	Control	Inter-vention				
**LTC_L**												
Heart Failure	0.3%	≤0.1%	0.8%	0.3%	1.2%	0.5%	1.6%	0.6%	0.34 [0.23-0.49]	0.37 [0.27-0.49]	0.43 [0.32-0.57]	0.38 [0.27-0.55]
Dementia	0.4%	≤0.1%	0.7%	0.3%	1.1%	0.4%	1.5%	0.6%	0.25 [0.17-0.37]	0.35 [0.26-0.48]	0.39 [0.29-0.54]	0.39 [0.27-0.56]
Atrial Fibrillation	0.5%	0.3%	1.2%	0.6%	1.7%	0.9%	2.5%	1.3%	0.51 [0.39-0.66]	0.49 [0.39-0.61]	0.53 [0.42-0.66]	0.52 [0.41-0.68]
Frailty	1.3%	0.4%	2.7%	1.0%	3.1%	1.4%	3.4%	1.8%	0.32 [0.26-0.39]	0.38 [0.32-0.44]	0.43 [0.36-0.52]	0.52 [0.42-0.65]
Physical Disability	≤0.1%	≤0.1%	0.3%	0.2%	0.5%	0.3%	0.6%	0.4%	0.75 [0.45-1.27]	0.66 [0.43-0.99]	0.62 [0.41-0.93]	0.66 [0.40-1.09]
Chronic Kidney Disease	0.4%	0.2%	0.7%	0.6%	1.0%	0.8%	1.5%	1.0%	0.46 [0.34-0.62]	0.78 [0.61-0.98]	0.75 [0.58-0.97]	0.66 [0.49-0.88]
Cerebrovascular Disease	0.5%	0.2%	1.0%	0.6%	1.5%	0.9%	2.0%	1.3%	0.42 [0.32-0.56]	0.62 [0.50-0.77]	0.62 [0.49-0.77]	0.67 [0.52-0.88]
Depression	0.7%	0.6%	1.5%	1.2%	2.3%	1.8%	2.9%	2.1%	0.81 [0.66-0.98]	0.80 [0.68-0.94]	0.75 [0.64-0.90]	0.72 [0.58-0.89]
Osteoporosis	0.7%	0.4%	1.5%	0.9%	2.0%	1.4%	2.7%	2.0%	0.65 [0.52-0.80]	0.63 [0.53-0.76]	0.68 [0.56-0.82]	0.74 [0.59-0.92]
Cancer	0.9%	0.5%	1.8%	1.2%	2.5%	1.7%	3.2%	2.5%	0.50 [0.41-0.62]	0.66 [0.56-0.77]	0.68 [0.58-0.81]	0.77 [0.63-0.94]
Peripheral Vascular Disease	0.3%	≤0.1%	0.5%	0.3%	0.8%	0.4%	0.9%	0.8%	0.38 [0.25-0.58]	0.55 [0.41-0.75]	0.47 [0.34-0.67]	0.80 [0.56-1.15]
Chronic Liver Disease	≤0.1%	≤0.1%	0.2%	≤0.1%	0.2%	0.2%	0.3%	0.3%	0.30 [0.13-0.70]	0.73 [0.44-1.21]	0.64 [0.37-1.13]	0.82 [0.44-1.50]
Hypertension	1.9%	1.4%	3.7%	2.8%	5.5%	4.3%	6.8%	5.7%	0.70 [0.62-0.80]	0.75 [0.67-0.84]	0.76 [0.68-0.86]	0.83 [0.73-0.95]
Coronary Heart Disease	0.7%	0.4%	1.2%	0.8%	1.8%	1.4%	2.3%	2.0%	0.53 [0.42-0.67]	0.68 [0.56-0.82]	0.75 [0.61-0.91]	0.86 [0.68-1.08]
**LTC_PL**												
Epilepsy	≤0.1%	≤0.1%	≤0.1%	≤0.1%	≤0.1%	≤0.1%	0.2%	≤0.1%	0.99 [0.37-2.65]	0.55 [0.26-1.14]	0.73 [0.34-1.54]	0.44 [0.17-1.12]
Pulmonary Heart Disease	0.2%	≤0.1%	0.3%	0.2%	0.5%	0.3%	0.6%	0.3%	0.51 [0.31-0.82]	0.49 [0.33-0.74]	0.66 [0.45-0.99]	0.52 [0.31-0.87]
COPD	0.5%	0.2%	1.0%	0.5%	1.4%	0.8%	1.8%	1.0%	0.44 [0.33-0.59]	0.49 [0.38-0.62]	0.54 [0.42-0.69]	0.52 [0.39-0.70]
Neurological Organ Failure	≤0.1%	≤0.1%	≤0.1%	≤0.1%	≤0.1%	≤0.1%	≤0.1%	≤0.1%	0.13 [0.03-0.58]	0.28 [0.09-0.84]	0.20 [0.04-0.89]	0.53 [0.14-2.07]
Multiple Sclerosis	≤0.1%	≤0.1%	≤0.1%	≤0.1%	≤0.1%	≤0.1%	≤0.1%	≤0.1%	0.25 [0.03-2.22]	0.87 [0.27-2.86]	0.95 [0.26-3.54]	0.62 [0.11-3.41]
Sarcoidosis	≤0.1%	≤0.1%	≤0.1%	≤0.1%	≤0.1%	≤0.1%	≤0.1%	≤0.1%	0.50 [0.09-2.71]	0.35 [0.07-1.73]	0.79 [0.22-2.81]	0.62 [0.16-2.50]
Bronchiectasis	≤0.1%	≤0.1%	0.2%	0.2%	0.3%	0.3%	0.5%	0.3%	0.86 [0.51-1.46]	0.94 [0.60-1.48]	0.93 [0.61-1.41]	0.69 [0.41-1.15]

	Percentage								Unadjusted OR (95% CI)			
	6 months		12 months		18 months		24 months		6 months	12 months	18 months	24 months
	Control	Inter-vention	Control	Inter-vention	Control	Inter-vention	Control	Inter-vention				
Serious Mental Illness	≤0.1%	≤0.1%	≤0.1%	≤0.1%	≤0.1%	≤0.1%	0.2%	0.2%	0.52 [0.25-1.08]	0.72 [0.41-1.28]	1.01 [0.53-1.93]	0.73 [0.34-1.60]
Severe Interstitial Lung Disease	≤0.1%	≤0.1%	≤0.1%	≤0.1%	0.2%	≤0.1%	0.2%	0.2%	0.73 [0.33-1.59]	0.84 [0.43-1.62]	0.76 [0.39-1.48]	0.73 [0.34-1.60]
Inflammatory Bowel Disease	≤0.1%	≤0.1%	≤0.1%	≤0.1%	0.2%	≤0.1%	0.2%	0.2%	0.88 [0.45-1.73]	0.90 [0.49-1.67]	0.69 [0.36-1.34]	0.73 [0.34-1.60]
Chronic Pain	0.4%	0.2%	0.7%	0.6%	1.1%	0.8%	1.4%	1.1%	0.61 [0.45-0.83]	0.76 [0.60-0.96]	0.80 [0.62-1.03]	0.78 [0.58-1.05]
Asthma	0.5%	0.3%	0.9%	0.8%	1.5%	1.1%	2.0%	1.6%	0.72 [0.56-0.93]	0.88 [0.71-1.08]	0.73 [0.59-0.91]	0.80 [0.62-1.02]
Rheumatoid Arthritis	≤0.1%	≤0.1%	0.3%	0.2%	0.4%	0.4%	0.6%	0.5%	0.56 [0.35-0.92]	0.71 [0.49-1.04]	0.85 [0.58-1.25]	0.91 [0.58-1.44]
Osteoarthritis	1.2%	0.8%	2.4%	1.8%	3.7%	2.9%	4.6%	4.4%	0.68 [0.58-0.80]	0.77 [0.67-0.88]	0.78 [0.68-0.89]	0.96 [0.82-1.12]
Alcohol Dependence	≤0.1%	≤0.1%	≤0.1%	≤0.1%	≤0.1%	≤0.1%	≤0.1%	≤0.1%	0.40 [0.08-2.05]	0.35 [0.07-1.73]	0.24 [0.03-2.04]	1.25 [0.18-8.86]
Parkinson's Disease	≤0.1%	≤0.1%	≤0.1%	≤0.1%	≤0.1%	≤0.1%	≤0.1%	≤0.1%	0.88 [0.34-2.29]	1.31 [0.52-3.32]	1.59 [0.55-4.57]	1.46 [0.49-4.34]

Data are presented as odds ratios with 95% confidence intervals. N (intervention and control) = 31,143 and 30,944 at 6 months, 20,952 and 21,928 at 12 months, 12,332 and 14,663 at 18 months and 6,504 and 8,116 at 24 months

Men

	Percentage								Unadjusted OR (95% CI)			
	6 months		12 months		18 months		24 months		6 months	12 months	18 months	24 months
	Control	Inter-vention	Control	Inter-vention	Control	Inter-vention	Control	Inter-vention				
**LTC_L**												
Dementia	0.3%	0.2%	0.5%	0.3%	0.9%	0.5%	1.3%	0.6%	0.57 [0.39-0.85]	0.53 [0.38-0.74]	0.55 [0.39-0.77]	0.47 [0.31-0.70]
Frailty	1.2%	0.6%	2.3%	1.3%	2.7%	1.9%	3.1%	1.9%	0.51 [0.42-0.62]	0.59 [0.50-0.69]	0.67 [0.56-0.80]	0.60 [0.48-0.77]
Osteoporosis	0.3%	≤0.1%	0.5%	0.3%	0.6%	0.4%	0.8%	0.5%	0.54 [0.36-0.82]	0.52 [0.37-0.75]	0.68 [0.47-0.99]	0.66 [0.42-1.05]
Chronic Liver Disease	≤0.1%	≤0.1%	0.2%	≤0.1%	0.3%	0.2%	0.3%	0.2%	0.43 [0.22-0.82]	0.62 [0.35-1.09]	0.72 [0.41-1.27]	0.70 [0.34-1.41]
Physical Disability	≤0.1%	≤0.1%	0.2%	≤0.1%	0.3%	0.2%	0.4%	0.3%	0.57 [0.30-1.07]	0.57 [0.34-0.94]	0.57 [0.33-0.99]	0.72 [0.39-1.34]
Cerebrovascular Disease	0.5%	0.3%	1.0%	0.7%	1.5%	1.1%	1.9%	1.5%	0.58 [0.44-0.78]	0.66 [0.52-0.83]	0.73 [0.58-0.93]	0.78 [0.59-1.04]
Peripheral Vascular Disease	0.4%	0.3%	1.0%	0.7%	1.4%	1.0%	1.7%	1.4%	0.66 [0.49-0.89]	0.71 [0.56-0.90]	0.74 [0.58-0.94]	0.84 [0.62-1.12]
Heart Failure	0.6%	0.3%	1.1%	0.6%	1.5%	1.1%	1.9%	1.7%	0.46 [0.35-0.62]	0.54 [0.43-0.69]	0.72 [0.57-0.91]	0.88 [0.67-1.17]
Depression	0.5%	0.4%	1.0%	0.8%	1.4%	1.3%	1.9%	1.7%	0.67 [0.52-0.87]	0.78 [0.62-0.98]	0.91 [0.72-1.14]	0.89 [0.68-1.17]
Chronic Kidney Disease	0.4%	0.2%	0.8%	0.5%	1.0%	0.8%	1.4%	1.3%	0.68 [0.49-0.93]	0.63 [0.48-0.82]	0.83 [0.63-1.10]	0.90 [0.66-1.23]
Atrial Fibrillation	0.8%	0.6%	1.5%	1.1%	2.2%	1.9%	3.1%	2.9%	0.73 [0.59-0.91]	0.74 [0.61-0.89]	0.88 [0.73-1.06]	0.95 [0.76-1.17]
Hypertension	2.1%	1.5%	3.9%	3.1%	5.5%	5.2%	7.5%	7.1%	0.73 [0.64-0.83]	0.80 [0.72-0.90]	0.94 [0.84-1.06]	0.95 [0.82-1.09]
Cancer	1.2%	0.7%	2.1%	1.7%	3.0%	2.8%	3.9%	3.8%	0.55 [0.45-0.66]	0.82 [0.71-0.96]	0.93 [0.80-1.09]	0.97 [0.80-1.17]
Coronary Heart Disease	1.0%	0.7%	1.8%	1.3%	2.6%	2.3%	3.1%	3.2%	0.67 [0.55-0.82]	0.74 [0.63-0.88]	0.86 [0.73-1.03]	1.04 [0.85-1.28]
**LTC_PL**												
Epilepsy	≤0.1%	≤0.1%	≤0.1%	≤0.1%	0.2%	≤0.1%	0.3%	≤0.1%	0.25 [0.09-0.65]	0.43 [0.21-0.90]	0.50 [0.23-1.10]	0.38 [0.15-0.96]
Alcohol Dependence	≤0.1%	≤0.1%	≤0.1%	≤0.1%	0.2%	≤0.1%	0.3%	≤0.1%	0.13 [0.04-0.45]	0.37 [0.17-0.80]	0.36 [0.16-0.80]	0.43 [0.17-1.09]
Pulmonary Heart Disease	0.2%	≤0.1%	0.3%	≤0.1%	0.5%	0.2%	0.5%	0.3%	0.32 [0.17-0.60]	0.36 [0.22-0.59]	0.48 [0.29-0.78]	0.52 [0.28-0.95]
Parkinson's Disease	≤0.1%	≤0.1%	≤0.1%	≤0.1%	0.2%	0.2%	0.3%	0.2%	0.80 [0.33-1.94]	0.71 [0.37-1.36]	0.92 [0.53-1.61]	0.61 [0.29-1.26]
Rheumatoid Arthritis	≤0.1%	≤0.1%	0.2%	≤0.1%	0.3%	0.2%	0.4%	0.3%	0.68 [0.36-1.29]	0.69 [0.39-1.22]	0.85 [0.50-1.45]	0.63 [0.33-1.20]
Chronic Pain	0.2%	≤0.1%	0.3%	0.3%	0.6%	0.4%	0.8%	0.5%	0.69 [0.43-1.09]	0.78 [0.53-1.15]	0.68 [0.46-1.00]	0.67 [0.42-1.07]
Neurological Organ Failure	≤0.1%	≤0.1%	≤0.1%	≤0.1%	≤0.1%	≤0.1%	0.2%	0.2%	0.20 [0.04-0.90]	0.40 [0.14-1.12]	0.62 [0.28-1.39]	0.70 [0.29-1.66]
COPD	0.6%	0.3%	1.1%	0.7%	1.8%	1.1%	2.3%	1.6%	0.45 [0.34-0.59]	0.59 [0.47-0.74]	0.60 [0.47-0.75]	0.71 [0.54-0.93]
Inflammatory Bowel Disease	≤0.1%	≤0.1%	≤0.1%	≤0.1%	0.2%	≤0.1%	0.3%	0.2%	0.70 [0.31-1.58]	0.52 [0.25-1.07]	0.48 [0.24-0.96]	0.81 [0.39-1.69]

	Percentage								Unadjusted OR (95% CI)			
	6 months		12 months		18 months		24 months		6 months	12 months	18 months	24 months
	Control	Inter-vention	Control	Inter-vention	Control	Inter-vention	Control	Inter-vention				
Serious Mental Illness	≤0.1%	≤0.1%	≤0.1%	≤0.1%	0.2%	≤0.1%	0.2%	0.2%	0.40 [0.17-0.98]	0.50 [0.24-1.02]	0.54 [0.26-1.10]	0.84 [0.36-1.97]
Asthma	0.4%	0.3%	0.8%	0.6%	1.2%	1.0%	1.3%	1.4%	0.70 [0.52-0.94]	0.68 [0.52-0.88]	0.84 [0.65-1.09]	1.03 [0.76-1.42]
Osteoarthritis	0.9%	0.5%	1.7%	1.3%	2.4%	2.3%	3.2%	3.3%	0.61 [0.49-0.75]	0.76 [0.64-0.90]	0.95 [0.80-1.13]	1.04 [0.85-1.28]
Bronchiectasis	≤0.1%	≤0.1%	0.2%	0.2%	0.4%	0.3%	0.4%	0.5%	0.91 [0.52-1.58]	0.82 [0.52-1.29]	0.91 [0.58-1.42]	1.17 [0.67-2.05]
Severe Interstitial Lung Disease	≤0.1%	≤0.1%	≤0.1%	≤0.1%	0.2%	≤0.1%	0.2%	0.3%	0.56 [0.28-1.10]	0.99 [0.54-1.82]	0.69 [0.35-1.38]	1.30 [0.64-2.63]
Multiple Sclerosis	≤0.1%	≤0.1%	≤0.1%	≤0.1%	≤0.1%	≤0.1%	≤0.1%	≤0.1%	1.97 [0.18-21.7]	n/a	n/a	n/a
Sarcoidosis	≤0.1%	≤0.1%	≤0.1%	≤0.1%	≤0.1%	≤0.1%	≤0.1%	≤0.1%	1.97 [0.18-21.7]	0.78 [0.17-3.48]	0.23 [0.03-2.01]	n/a

Data are presented as odds ratios with 95% confidence intervals. N (intervention and control) = 25,797 and 25,371 at 6 months, 17,188 and 17,845 at 12 months, 10,165 and 11,937 at 18 months and 5,317 and 6,478 at 24 months

Supplementary table S11: Percentage of participants acquiring new conditions at 6, 12, 18 and 24 follow-up periods split by age

Less than 70 years

	**Percentage**								**Unadjusted OR (95% CI)**			
	6 months		12 months		18 months		24 months		6 months	12 months	18 months	24 months
	Control	Inter-vention	Control	Inter-vention	Control	Inter-vention	Control	Inter-vention				
**LTC_L**												
Dementia	≤0.1%	≤0.1%	≤0.1%	≤0.1%	≤0.1%	≤0.1%	0.3%	0.2%	1.04 [0.52-2.05]	0.91 [0.52-1.59]	1.09 [0.55-2.16]	0.52 [0.26-1.02]
Chronic Liver Disease	≤0.1%	≤0.1%	0.2%	≤0.1%	0.3%	0.2%	0.4%	0.3%	0.34 [0.17-0.67]	0.62 [0.38-1.01]	0.71 [0.43-1.18]	0.71 [0.39-1.29]
Depression	0.8%	0.6%	1.5%	1.3%	2.2%	1.8%	2.7%	2.1%	0.76 [0.63-0.92]	0.86 [0.74-1.01]	0.83 [0.70-0.99]	0.78 [0.63-0.96]
Heart Failure	0.2%	≤0.1%	0.4%	0.2%	0.6%	0.4%	0.7%	0.6%	0.45 [0.30-0.69]	0.54 [0.37-0.77]	0.67 [0.47-0.95]	0.81 [0.53-1.22]
Frailty	0.3%	0.2%	0.5%	0.4%	0.6%	0.7%	0.8%	0.7%	0.56 [0.40-0.78]	0.85 [0.65-1.11]	1.12 [0.84-1.50]	0.86 [0.59-1.27]
Cancer	0.7%	0.4%	1.4%	1.1%	2.0%	1.7%	2.6%	2.4%	0.59 [0.48-0.72]	0.79 [0.67-0.94]	0.86 [0.72-1.03]	0.89 [0.72-1.10]
Hypertension	1.7%	1.3%	3.1%	2.5%	4.5%	4.0%	5.9%	5.3%	0.75 [0.66-0.85]	0.81 [0.73-0.91]	0.88 [0.78-0.99]	0.89 [0.77-1.02]
Peripheral Vascular Disease	0.2%	≤0.1%	0.4%	0.3%	0.6%	0.4%	0.7%	0.7%	0.76 [0.51-1.14]	0.72 [0.52-1.01]	0.73 [0.52-1.02]	0.93 [0.63-1.36]
Chronic Kidney Disease	≤0.1%	≤0.1%	0.3%	0.3%	0.3%	0.4%	0.5%	0.5%	0.95 [0.62-1.47]	1.13 [0.79-1.62]	1.20 [0.82-1.76]	0.98 [0.62-1.54]
Coronary Heart Disease	0.6%	0.5%	1.1%	0.9%	1.5%	1.4%	2.0%	2.0%	0.71 [0.57-0.87]	0.81 [0.67-0.98]	0.95 [0.78-1.15]	1.00 [0.79-1.25]
Physical Disability	≤0.1%	≤0.1%	0.2%	≤0.1%	0.2%	0.2%	0.3%	0.3%	0.98 [0.52-1.85]	0.89 [0.56-1.42]	0.75 [0.45-1.26]	1.01 [0.56-1.83]
Osteoporosis	0.2%	0.2%	0.4%	0.4%	0.5%	0.6%	0.8%	0.8%	0.90 [0.64-1.28]	1.00 [0.75-1.35]	1.19 [0.88-1.61]	1.02 [0.71-1.45]
Atrial Fibrillation	0.2%	0.2%	0.5%	0.5%	0.7%	0.8%	1.1%	1.2%	0.95 [0.70-1.30]	1.02 [0.78-1.32]	1.06 [0.81-1.39]	1.09 [0.81-1.47]
Cerebrovascular Disease	0.2%	0.2%	0.5%	0.4%	0.6%	0.6%	0.8%	1.0%	0.63 [0.45-0.90]	0.93 [0.71-1.23]	0.97 [0.72-1.31]	1.21 [0.86-1.71]
**LTC_PL**												
Sarcoidosis	≤0.1%	≤0.1%	≤0.1%	≤0.1%	≤0.1%	≤0.1%	≤0.1%	≤0.1%	0.33 [0.03-3.13]	0.34 [0.07-1.70]	0.43 [0.12-1.64]	0.15 [0.02-1.21]
Alcohol Dependence	≤0.1%	≤0.1%	≤0.1%	≤0.1%	0.2%	≤0.1%	0.2%	≤0.1%	0.19 [0.06-0.54]	0.20 [0.08-0.51]	0.28 [0.11-0.68]	0.43 [0.17-1.08]
Epilepsy	≤0.1%	≤0.1%	≤0.1%	≤0.1%	≤0.1%	≤0.1%	0.2%	≤0.1%	0.49 [0.21-1.14]	0.61 [0.31-1.20]	0.85 [0.39-1.85]	0.53 [0.22-1.29]
COPD	0.4%	0.2%	0.7%	0.4%	1.1%	0.7%	1.5%	0.9%	0.55 [0.41-0.75]	0.56 [0.43-0.73]	0.63 [0.49-0.82]	0.60 [0.44-0.81]
Multiple Sclerosis	≤0.1%	≤0.1%	≤0.1%	≤0.1%	≤0.1%	≤0.1%	≤0.1%	≤0.1%	1.47 [0.24-8.77]	1.03 [0.26-4.11]	0.93 [0.25-3.45]	0.61 [0.11-3.31]

	Percentage								Unadjusted OR (95% CI)			
	6 months		12 months		18 months		24 months		6 months	12 months	18 months	24 months
	Control	Inter-vention	Control	Inter-vention	Control	Inter-vention	Control	Inter-vention				
Chronic Pain	0.2%	0.2%	0.5%	0.4%	0.8%	0.6%	1.1%	0.7%	0.71 [0.50-0.99]	0.86 [0.66-1.13]	0.75 [0.56-1.00]	0.63 [0.44-0.89]
Rheumatoid Arthritis	≤0.1%	≤0.1%	≤0.1%	0.2%	0.2%	0.3%	0.4%	0.3%	0.93 [0.51-1.70]	1.13 [0.69-1.83]	1.06 [0.66-1.70]	0.64 [0.37-1.11]
Pulmonary Heart Disease	≤0.1%	≤0.1%	0.2%	≤0.1%	0.3%	0.2%	0.4%	0.2%	0.40 [0.21-0.79]	0.57 [0.35-0.93]	0.75 [0.46-1.23]	0.67 [0.36-1.23]
Inflammatory Bowel Disease	≤0.1%	≤0.1%	≤0.1%	≤0.1%	0.2%	≤0.1%	0.2%	0.2%	0.79 [0.38-1.65]	0.73 [0.38-1.42]	0.54 [0.29-1.02]	0.83 [0.41-1.68]
Asthma	0.5%	0.3%	0.9%	0.6%	1.3%	1.0%	1.7%	1.4%	0.68 [0.53-0.87]	0.76 [0.61-0.94]	0.75 [0.60-0.94]	0.85 [0.65-1.10]
Serious Mental Illness	≤0.1%	≤0.1%	0.2%	≤0.1%	0.2%	0.2%	0.3%	0.2%	0.59 [0.32-1.08]	0.68 [0.41-1.12]	0.89 [0.52-1.53]	0.86 [0.45-1.68]
Severe Interstitial Lung Disease	≤0.1%	≤0.1%	≤0.1%	≤0.1%	≤0.1%	≤0.1%	≤0.1%	≤0.1%	1.12 [0.40-3.08]	1.16 [0.45-2.99]	0.63 [0.23-1.71]	0.97 [0.26-3.61]
Osteoarthritis	0.8%	0.5%	1.4%	1.2%	2.2%	2.0%	3.0%	3.0%	0.72 [0.59-0.87]	0.86 [0.73-1.01]	0.88 [0.74-1.03]	0.99 [0.82-1.20]
Neurological Organ Failure	≤0.1%	≤0.1%	≤0.1%	≤0.1%	≤0.1%	≤0.1%	≤0.1%	≤0.1%	0.59 [0.14-2.45]	0.77 [0.27-2.22]	0.29 [0.06-1.36]	1.21 [0.35-4.19]
Bronchiectasis	≤0.1%	≤0.1%	≤0.1%	≤0.1%	0.2%	0.3%	0.2%	0.3%	1.24 [0.56-2.74]	1.12 [0.63-1.97]	1.27 [0.78-2.09]	1.21 [0.65-2.25]
Parkinson's Disease	≤0.1%	≤0.1%	≤0.1%	≤0.1%	≤0.1%	≤0.1%	≤0.1%	≤0.1%	2.20 [0.68-7.14]	2.05 [0.70-6.01]	1.93 [0.70-5.31]	1.70 [0.54-5.35]

Data are presented as odds ratios with 95% confidence intervals. N (intervention and control) = 33,453 and 32,679 at 6 months, 22,070 and 22,662 at 12 months, 12,951 and 14,996 at 18 months and 6,799 and 8,235 at 24 months

70 years and over

	Percentage								Unadjusted OR (95% CI)			
	6 months		12 months		18 months		24 months		6 months	12 months	18 months	24 months
	Control	Inter-vention	Control	Inter-vention	Control	Inter-vention	Control	Inter-vention				
**LTC_L**												
Dementia	0.7%	0.2%	1.3%	0.5%	2.1%	0.9%	2.8%	1.2%	0.31 [0.23-0.42]	0.37 [0.29-0.48]	0.42 [0.32-0.53]	0.41 [0.31-0.56]
Frailty	2.6%	1.0%	5.1%	2.2%	6.0%	2.8%	6.5%	3.4%	0.38 [0.33-0.44]	0.41 [0.36-0.47]	0.46 [0.40-0.53]	0.51 [0.43-0.61]
Physical Disability	0.2%	≤0.1%	0.4%	0.2%	0.6%	0.3%	0.8%	0.4%	0.53 [0.31-0.90]	0.46 [0.29-0.72]	0.53 [0.34-0.81]	0.53 [0.31-0.89]
Heart Failure	0.8%	0.3%	1.6%	0.7%	2.4%	1.3%	3.1%	1.8%	0.40 [0.31-0.53]	0.44 [0.36-0.55]	0.56 [0.45-0.69]	0.58 [0.45-0.75]
Cerebrovascular Disease	0.9%	0.4%	1.7%	0.9%	2.7%	1.6%	3.4%	2.0%	0.44 [0.34-0.56]	0.54 [0.44-0.65]	0.58 [0.48-0.71]	0.58 [0.46-0.73]
Osteoporosis	0.9%	0.5%	1.9%	0.9%	2.5%	1.4%	3.2%	2.0%	0.53 [0.42-0.67]	0.50 [0.41-0.61]	0.54 [0.44-0.67]	0.63 [0.49-0.80]
Atrial Fibrillation	1.2%	0.6%	2.5%	1.3%	3.4%	2.1%	4.8%	3.1%	0.54 [0.45-0.66]	0.52 [0.44-0.61]	0.61 [0.52-0.73]	0.63 [0.52-0.77]
Chronic Kidney Disease	0.8%	0.4%	1.4%	0.9%	1.9%	1.3%	2.8%	2.0%	0.46 [0.36-0.60]	0.61 [0.50-0.75]	0.70 [0.56-0.87]	0.72 [0.56-0.92]
Depression	0.5%	0.4%	1.1%	0.7%	1.6%	1.2%	2.1%	1.6%	0.73 [0.55-0.97]	0.64 [0.51-0.82]	0.74 [0.59-0.94]	0.76 [0.58-1.00]
Peripheral Vascular Disease	0.6%	0.3%	1.2%	0.7%	1.7%	1.0%	2.0%	1.6%	0.46 [0.34-0.62]	0.62 [0.50-0.78]	0.59 [0.46-0.76]	0.79 [0.59-1.04]
Chronic Liver Disease	≤0.1%	≤0.1%	≤0.1%	≤0.1%	0.2%	≤0.1%	0.3%	0.2%	0.43 [0.20-0.94]	0.77 [0.42-1.41]	0.63 [0.33-1.20]	0.84 [0.41-1.75]
Cancer	1.5%	0.7%	2.7%	1.9%	3.6%	2.8%	4.6%	4.0%	0.48 [0.40-0.58]	0.71 [0.61-0.82]	0.78 [0.66-0.91]	0.87 [0.72-1.04]
Hypertension	2.4%	1.6%	4.6%	3.5%	6.8%	5.6%	8.7%	7.8%	0.68 [0.60-0.78]	0.74 [0.67-0.83]	0.82 [0.73-0.92]	0.89 [0.78-1.02]
Coronary Heart Disease	1.0%	0.6%	2.0%	1.4%	3.0%	2.2%	3.5%	3.3%	0.53 [0.43-0.65]	0.66 [0.55-0.78]	0.73 [0.62-0.87]	0.94 [0.77-1.15]
**LTC_PL**												
Epilepsy	≤0.1%	≤0.1%	≤0.1%	≤0.1%	0.2%	≤0.1%	0.3%	≤0.1%	0.42 [0.15-1.19]	0.37 [0.17-0.83]	0.46 [0.21-0.98]	0.32 [0.12-0.84]
Pulmonary Heart Disease	0.3%	≤0.1%	0.5%	0.2%	0.7%	0.4%	0.9%	0.4%	0.44 [0.27-0.69]	0.36 [0.24-0.55]	0.50 [0.34-0.75]	0.45 [0.27-0.75]
Neurological Organ Failure	≤0.1%	≤0.1%	≤0.1%	≤0.1%	0.2%	≤0.1%	0.3%	≤0.1%	0.05 [0.01-0.37]	0.16 [0.05-0.54]	0.52 [0.24-1.14]	0.47 [0.19-1.21]
Serious Mental Illness	≤0.1%	≤0.1%	≤0.1%	≤0.1%	≤0.1%	≤0.1%	≤0.1%	≤0.1%	0.10 [0.01-0.79]	0.46 [0.18-1.19]	0.43 [0.16-1.20]	0.56 [0.17-1.83]
Parkinson's Disease	≤0.1%	≤0.1%	≤0.1%	≤0.1%	0.2%	0.2%	0.4%	0.2%	0.50 [0.22-1.18]	0.64 [0.34-1.21]	0.87 [0.49-1.54]	0.60 [0.29-1.24]
COPD	0.8%	0.3%	1.5%	0.8%	2.3%	1.2%	2.8%	1.8%	0.38 [0.29-0.50]	0.53 [0.43-0.65]	0.54 [0.43-0.67]	0.64 [0.49-0.82]

	Percentage								Unadjusted OR (95% CI)			
	6 months		12 months		18 months		24 months		6 months	12 months	18 months	24 months
	Control	Inter-vention	Control	Inter-vention	Control	Inter-vention	Control	Inter-vention				
Inflammatory Bowel Disease	≤0.1%	≤0.1%	≤0.1%	≤0.1%	0.2%	≤0.1%	0.3%	0.2%	0.82 [0.39-1.70]	0.69 [0.36-1.33]	0.64 [0.31-1.32]	0.71 [0.31-1.61]
Bronchiectasis	0.2%	0.2%	0.4%	0.3%	0.6%	0.4%	0.7%	0.5%	0.80 [0.52-1.25]	0.80 [0.54-1.18]	0.76 [0.51-1.13]	0.73 [0.45-1.19]
Chronic Pain	0.3%	0.2%	0.7%	0.4%	0.9%	0.7%	1.1%	1.0%	0.55 [0.37-0.81]	0.66 [0.49-0.90]	0.78 [0.57-1.06]	0.91 [0.63-1.32]
Asthma	0.4%	0.3%	0.9%	0.7%	1.4%	1.1%	1.7%	1.6%	0.76 [0.56-1.04]	0.83 [0.65-1.06]	0.81 [0.63-1.04]	0.92 [0.69-1.24]
Rheumatoid Arthritis	0.2%	≤0.1%	0.4%	0.2%	0.5%	0.4%	0.6%	0.6%	0.45 [0.26-0.76]	0.51 [0.33-0.78]	0.74 [0.49-1.11]	0.98 [0.60-1.62]
Osteoarthritis	1.5%	0.9%	3.0%	2.2%	4.3%	3.6%	5.2%	5.2%	0.61 [0.51-0.72]	0.71 [0.62-0.82]	0.82 [0.71-0.94]	0.99 [0.84-1.17]
Severe Interstitial Lung Disease	≤0.1%	≤0.1%	0.2%	0.2%	0.3%	0.2%	0.4%	0.4%	0.52 [0.28-0.95]	0.87 [0.52-1.45]	0.77 [0.45-1.34]	1.03 [0.59-1.81]
Alcohol Dependence	≤0.1%	≤0.1%	≤0.1%	≤0.1%	≤0.1%	≤0.1%	≤0.1%	≤0.1%	0.17 [0.02-1.39]	1.28 [0.39-4.19]	0.61 [0.15-2.43]	1.27 [0.18-8.99]
Sarcoidosis	≤0.1%	≤0.1%	≤0.1%	≤0.1%	≤0.1%	≤0.1%	≤0.1%	≤0.1%	1.51 [0.25-9.04]	0.80 [0.18-3.57]	0.81 [0.14-4.85]	1.27 [0.18-8.99]
Multiple Sclerosis	≤0.1%	≤0.1%	≤0.1%	≤0.1%	≤0.1%	≤0.1%	≤0.1%	≤0.1%	n/a	1.06 [0.15-7.56]	n/a	n/a

Data are presented as odds ratios with 95% confidence intervals. N (intervention and control) = 23,487 and 23,636 at 6 months, 16,070 and 17,111 at 12 months, 9,546 and 11,604 at 18 months and 5,022 and 6,359 at 24 months

Supplementary table S12: Secondary analyses: Baseline characteristics of the 6-months cohort for those referred to the NHS DPP and completed the programme (intervention group) compared to those not referred to the NHS DPP (control group)

	N		%		
	Not referred to the NHS DPP	Referred to the NHS DPP and completed	Not referred to the NHS DPP	Referred to the NHS DPP and completed	P value
Total	56,429	56,940	100%	100%	
Number of LTC_Ls					
0	28,301	28,379	50.2%	49.8%	
At least 1	28,128	28,561	49.8%	50.2%	0.292
At least 2	14,191	13,697	25.1%	24.1%	<0.001
At least 3	6,290	5,634	11.1%	9.9%	<0.001
At least 4	2,667	2,134	4.7%	3.7%	<0.001
Number of LTC_PLs					
0	40,037	40,601	71.0%	71.3%	
At least 1	16,392	16,339	29.0%	28.7%	0.189
At least 2	4,536	4,368	8.0%	7.7%	0.022
At least 3	1,301	1,179	2.3%	2.1%	0.007
At least 4	329	275	0.6%	0.5%	0.021
Number of LTCs					
0	24,342	24,151	43.1%	42.4%	
At least 1	32,087	32,789	56.9%	57.6%	0.014
At least 2	20,227	20,104	35.8%	35.3%	0.059
At least 3	11,228	10,665	19.9%	18.7%	<0.001
At least 4	6,018	5,322	10.7%	9.3%	<0.001
**LTC_L**					
Atrial Fibrillation	3,984	3,894	7.1%	6.8%	0.143
Cancer	4,710	4,554	8.3%	8.0%	0.032
Cerebrovascular Disease	2,027	1,961	3.6%	3.4%	0.176
Chronic Kidney Disease	1,379	1,233	2.4%	2.2%	0.002
Chronic Liver Disease	307	282	0.5%	0.5%	0.253
Coronary Heart Disease	7,614	7,730	13.5%	13.6%	0.684
Dementia	722	432	1.3%	0.8%	<0.001
Depression	4,621	4,393	8.2%	7.7%	0.003
Frailty	1,193	630	2.1%	1.1%	<0.001
Heart Failure	1,819	1,583	3.2%	2.8%	<0.001
Hypertension	19,978	20,224	35.4%	35.5%	0.687
Osteoporosis	2,219	2,023	3.9%	3.6%	0.001
Peripheral Vascular Disease	1,917	1,770	3.4%	3.1%	0.006
Physical Disability	454	409	0.8%	0.7%	0.095
**LTC_PL**					
Alcohol Dependence	228	193	0.4%	0.3%	0.072
Asthma	5,293	5,303	9.4%	9.3%	0.7
Bronchiectasis	476	468	0.8%	0.8%	0.689
Chronic Pain	1,557	1,512	2.8%	2.7%	0.282
COPD	2,299	2,125	4.1%	3.7%	0.003
Epilepsy	483	425	0.9%	0.7%	0.039
Inflammatory Bowel Disease	672	682	1.2%	1.2%	0.915
Multiple Sclerosis	133	127	0.2%	0.2%	0.656
Neurological Organ Failure	79	40	0.1%	0.1%	<0.001
Osteoarthritis	8,831	8,948	15.6%	15.7%	0.763

	N		%		
	Not referred to the NHS DPP	Referred to the NHS DPP and completed	Not referred to the NHS DPP	Referred to the NHS DPP and completed	P value
Parkinson's Disease	142	120	0.3%	0.2%	0.152
Pulmonary Heart Disease	702	676	1.2%	1.2%	0.383
Rheumatoid Arthritis	803	745	1.4%	1.3%	0.096
Sarcoidosis	100	109	0.2%	0.2%	0.577
Serious Mental Illness	748	642	1.3%	1.1%	0.002
Severe Interstitial Lung Disease	136	134	0.2%	0.2%	0.845
Age~	65.6	66.0	13.8	10.6	<0.001
Sex					
Female	30,678	31,143	54.4%	54.7%	0.266
Male	25,751	25,797	45.6%	45.3%	
Ethnicity					
Asian	3,987	3,941	7.1%	6.9%	<0.001
Black	2,708	2,859	4.8%	5.0%	
Mixed	720	718	1.3%	1.3%	
Other	1,428	1,313	2.5%	2.3%	
Unknown	3,891	3,630	6.9%	6.4%	
White	43,695	44,479	77.4%	78.1%	
IMD quintile					
1 (most deprived)	8,010	7,527	14.2%	13.2%	<0.001
2	9,673	9,879	17.1%	17.3%	
3	11,740	11,815	20.8%	20.7%	
4	12,898	13,188	22.9%	23.2%	
5 (least deprived)	14,108	14,531	25.0%	25.5%	
Rural GP					
No	49,696	50,513	88.1%	88.7%	0.001
Yes	6,733	6,427	11.9%	11.3%	
IMD quintile GP					
1 (most deprived)	14,455	15,031	25.6%	26.4%	<0.001
2	13,203	12,535	23.4%	22.0%	
3	11,052	11,397	19.6%	20.0%	
4	9,758	10,443	17.3%	18.3%	
5 (least deprived)	7,961	7,534	14.1%	13.2%	
QOF overall quintile GP					
1 (Highest achievement)	9,609	8,952	17.0%	15.7%	<0.001
2	10,099	10,468	17.9%	18.4%	
3	11,290	12,115	20.0%	21.3%	
4	12,502	12,346	22.2%	21.7%	
5 (Lowest achievement)	12,929	13,059	22.9%	22.9%	
size quintile GP					
1 (Smallest)	10,045	10,410	17.8%	18.3%	0.02
2	11,100	11,267	19.7%	19.8%	
3	11,298	11,600	20.0%	20.4%	
4	11,885	11,754	21.1%	20.6%	
5 (Largest)	12,101	11,909	21.4%	20.9%	
FTE quintile GP					
1 (Least GPs per patient)	9,592	9,586	17.0%	16.8%	<0.001
2	11,000	11,914	19.5%	20.9%	
3	12,059	12,162	21.4%	21.4%	

	N		%		P value
	Not referred to the NHS DPP	Referred to the NHS DPP and completed	Not referred to the NHS DPP	Referred to the NHS DPP and completed	
4	12,173	12,072	21.6%	21.2%	
5 (Most GPs per patient)	11,605	11,206	20.6%	19.7%	
Arrivals~	0.3	0.3	0.7	0.8	0.8497
Admissions~	0.4	0.4	1.1	1.0	0.2833
Appointments~	3.1	3.3	5.9	5.5	0.002

~ Mean & SD reported rather than N & %. P values for categorical variables were calculated using two-sided chi-square tests and p values for continuous variables were calculated using two-sided t-tests

Supplementary table S13: Secondary analyses: Baseline characteristics of the 6-months cohort for those referred to the NHS DPP and completed the programme (intervention group) compared to those not referred to the NHS DPP (control group), by sex

	**Women**					**Men**				
	N		%			N		%		
	Not referred to the NHS DPP	Referred to the NHS DPP and completed	Not referred to the NHS DPP	Referred to the NHS DPP and completed	P value	Not referred to the NHS DPP	Referred to the NHS DPP and completed	Not referred to the NHS DPP	Referred to the NHS DPP and completed	P value
Total	30,678	31,143	100%	100%		25,751	25,797	100%	100%	
Number of LTC_Ls										
0	15,132	16,373	49.3%	52.6%		13,169	12,006	51.1%	46.5%	
At least 1	15,546	14,770	50.7%	47.4%	<0.001	12,582	13,791	48.9%	53.5%	<0.001
At least 2	7,504	6,307	24.5%	20.3%	<0.001	6,687	7,390	26.0%	28.6%	<0.001
At least 3	3,267	2,322	10.6%	7.5%	<0.001	3,023	3,312	11.7%	12.8%	<0.001
At least 4	1,375	791	4.5%	2.5%	<0.001	1,292	1,343	5.0%	5.2%	0.331
Number of LTC_PLs										
0	20,801	21,699	67.8%	69.7%		19,236	18,902	74.7%	73.3%	
At least 1	9,877	9,444	32.2%	30.3%	<0.001	6,515	6,895	25.3%	26.7%	<0.001
At least 2	2,931	2,684	9.6%	8.6%	<0.001	1,605	1,684	6.2%	6.5%	0.17
At least 3	915	744	3.0%	2.4%	<0.001	386	435	1.5%	1.7%	0.089
At least 4	243	172	0.8%	0.6%	<0.001	86	103	0.3%	0.4%	0.22
Number of LTCs										
0	12,817	13,712	41.8%	44.0%		11,525	10,439	44.8%	40.5%	
At least 1	17,861	17,431	58.2%	56.0%	<0.001	14,226	15,358	55.2%	59.5%	<0.001
At least 2	11,322	10,281	36.9%	33.0%	<0.001	8,905	9,823	34.6%	38.1%	<0.001
At least 3	6,348	5,288	20.7%	17.0%	<0.001	4,880	5,377	19.0%	20.8%	<0.001
At least 4	3,473	2,569	11.3%	8.2%	<0.001	2,545	2,753	9.9%	10.7%	0.003
**LTC_L**										
Atrial Fibrillation	1,875	1,423	6.1%	4.6%	<0.001	2,109	2,471	8.2%	9.6%	<0.001
Cancer	2,402	2,206	7.8%	7.1%	<0.001	2,308	2,348	9.0%	9.1%	0.582
Cerebrovascular Disease	1,081	900	3.5%	2.9%	<0.001	946	1,061	3.7%	4.1%	0.01

	Women					Men				
	N		%			N		%		
	Not referred to the NHS DPP	Referred to the NHS DPP and completed	Not referred to the NHS DPP	Referred to the NHS DPP and completed	P value	Not referred to the NHS DPP	Referred to the NHS DPP and completed	Not referred to the NHS DPP	Referred to the NHS DPP and completed	P value
Chronic Kidney Disease	809	634	2.6%	2.0%	<0.001	570	599	2.2%	2.3%	0.408
Chronic Liver Disease	154	119	0.5%	0.4%	0.025	153	163	0.6%	0.6%	0.583
Coronary Heart Disease	3,161	2,643	10.3%	8.5%	<0.001	4,453	5,087	17.3%	19.7%	<0.001
Dementia	470	202	1.5%	0.6%	<0.001	252	230	1.0%	0.9%	0.305
Depression	3,105	2,940	10.1%	9.4%	0.004	1,516	1,453	5.9%	5.6%	0.215
Frailty	767	307	2.5%	1.0%	<0.001	426	323	1.7%	1.3%	<0.001
Heart Failure	835	544	2.7%	1.7%	<0.001	984	1,039	3.8%	4.0%	0.228
Hypertension	11,044	10,206	36.0%	32.8%	<0.001	8,934	10,018	34.7%	38.8%	<0.001
Osteoporosis	1,867	1,614	6.1%	5.2%	<0.001	352	409	1.4%	1.6%	0.04
Peripheral Vascular Disease	819	651	2.7%	2.1%	<0.001	1,098	1,119	4.3%	4.3%	0.68
Physical Disability	234	206	0.8%	0.7%	0.134	220	203	0.9%	0.8%	0.396
**LTC_PL**										
Alcohol Dependence	61	45	0.2%	0.1%	0.102	167	148	0.6%	0.6%	0.276
Asthma	3,344	3,311	10.9%	10.6%	0.281	1,949	1,992	7.6%	7.7%	0.513
Bronchiectasis	281	265	0.9%	0.9%	0.387	195	203	0.8%	0.8%	0.7
Chronic Pain	1,161	1,092	3.8%	3.5%	0.065	396	420	1.5%	1.6%	0.412
COPD	1,219	960	4.0%	3.1%	<0.001	1,080	1,165	4.2%	4.5%	0.073
Epilepsy	268	225	0.9%	0.7%	0.035	215	200	0.8%	0.8%	0.449
Inflammatory Bowel Disease	347	333	1.1%	1.1%	0.461	325	349	1.3%	1.4%	0.364
Multiple Sclerosis	113	106	0.4%	0.3%	0.558	20	21	0.1%	0.1%	0.88
Neurological Organ Failure	49	22	0.2%	0.1%	<0.001	30	18	0.1%	0.1%	0.082
Osteoarthritis	5,599	5,374	18.3%	17.3%	0.001	3,232	3,574	12.6%	13.9%	<0.001
Parkinson's Disease	69	43	0.2%	0.1%	0.011	73	77	0.3%	0.3%	0.752
Pulmonary Heart Disease	399	308	1.3%	1.0%	<0.001	303	368	1.2%	1.4%	0.012
Rheumatoid Arthritis	597	522	1.9%	1.7%	0.012	206	223	0.8%	0.9%	0.42
Sarcoidosis	59	58	0.2%	0.2%	0.862	41	51	0.2%	0.2%	0.301

	Women					Men				
	N		%			N		%		
	Not referred to the NHS DPP	Referred to the NHS DPP and completed	Not referred to the NHS DPP	Referred to the NHS DPP and completed	P value	Not referred to the NHS DPP	Referred to the NHS DPP and completed	Not referred to the NHS DPP	Referred to the NHS DPP and completed	P value
Serious Mental Illness	416	375	1.4%	1.2%	0.093	332	267	1.3%	1.0%	0.007
Severe Interstitial Lung Disease	68	56	0.2%	0.2%	0.245	68	78	0.3%	0.3%	0.413
Age~	66.6	65.6	14.0	10.6	<0.001	64.3	66.5	13.5	10.6	<0.001
Ethnicity										
Asian	2,166	2,353	7.1%	7.6%	<0.001	1,821	1,588	7.1%	6.2%	<0.001
Black	1,550	1,939	5.1%	6.2%		1,158	920	4.5%	3.6%	
Mixed	397	422	1.3%	1.4%		323	296	1.3%	1.1%	
Other	818	793	2.7%	2.5%		610	520	2.4%	2.0%	
Unknown	1,781	1,791	5.8%	5.8%		2,110	1,839	8.2%	7.1%	
White	23,966	23,845	78.1%	76.6%		19,729	20,634	76.6%	80.0%	
IMD quintile										
1 (most deprived)	4,487	4,209	14.6%	13.5%	0.001	3,523	3,318	13.7%	12.9%	0.009
2	5,222	5,550	17.0%	17.8%		4,451	4,329	17.3%	16.8%	
3	6,463	6,532	21.1%	21.0%		5,277	5,283	20.5%	20.5%	
4	6,980	7,151	22.8%	23.0%		5,918	6,037	23.0%	23.4%	
5 (least deprived)	7,526	7,701	24.5%	24.7%		6,582	6,830	25.6%	26.5%	
Rural GP										
No	27,167	27,778	88.6%	89.2%	0.011	22,529	22,735	87.5%	88.1%	0.026
Yes	3,511	3,365	11.4%	10.8%		3,222	3,062	12.5%	11.9%	
IMD quintile GP										
1 (most deprived)	7,734	7,971	25.2%	25.6%	<0.001	6,721	7,060	26.1%	27.4%	<0.001
2	7,153	6,938	23.3%	22.3%		6,050	5,597	23.5%	21.7%	
3	6,032	6,243	19.7%	20.0%		5,020	5,154	19.5%	20.0%	
4	5,402	5,800	17.6%	18.6%		4,356	4,643	16.9%	18.0%	
5 (least deprived)	4,357	4,191	14.2%	13.5%		3,604	3,343	14.0%	13.0%	

	Women					Men				
	N		%			N		%		
	Not referred to the NHS DPP	Referred to the NHS DPP and completed	Not referred to the NHS DPP	Referred to the NHS DPP and completed	P value	Not referred to the NHS DPP	Referred to the NHS DPP and completed	Not referred to the NHS DPP	Referred to the NHS DPP and completed	P value
QOF overall quintile GP										
1 (Highest achievement)	5,154	4,852	16.8%	15.6%	<0.001	4,455	4,100	17.3%	15.9%	<0.001
2	5,419	5,707	17.7%	18.3%		4,680	4,761	18.2%	18.5%	
3	6,134	6,630	20.0%	21.3%		5,156	5,485	20.0%	21.3%	
4	6,949	6,718	22.7%	21.6%		5,553	5,628	21.6%	21.8%	
5 (Lowest achievement)	7,022	7,236	22.9%	23.2%		5,907	5,823	22.9%	22.6%	
size quintile GP										
1 (Smallest)	5,399	5,766	17.6%	18.5%	<0.001	4,646	4,644	18.0%	18.0%	0.999
2	6,025	6,198	19.6%	19.9%		5,075	5,069	19.7%	19.6%	
3	6,109	6,386	19.9%	20.5%		5,189	5,214	20.2%	20.2%	
4	6,513	6,361	21.2%	20.4%		5,372	5,393	20.9%	20.9%	
5 (Largest)	6,632	6,432	21.6%	20.7%		5,469	5,477	21.2%	21.2%	
FTE quintile GP										
1 (Least GPs per patient)	5,190	5,291	16.9%	17.0%	<0.001	4,402	4,295	17.1%	16.6%	0.032
2	5,942	6,592	19.4%	21.2%		5,058	5,322	19.6%	20.6%	
3	6,551	6,630	21.4%	21.3%		5,508	5,532	21.4%	21.4%	
4	6,670	6,545	21.7%	21.0%		5,503	5,527	21.4%	21.4%	
5 (Most GPs per patient)	6,325	6,085	20.6%	19.5%		5,280	5,121	20.5%	19.9%	
Arrivals~	0.3	0.3	0.8	0.7	0.334	0.3	0.3	0.7	0.8	0.4687
Admissions~	0.4	0.4	1.1	1.0	0.1652	0.4	0.4	1.1	1.1	0.0304
Appointments~	3.4	3.4	6.1	5.7	0.7048	2.9	3.2	5.6	5.3	0.048

~ Mean & SD reported rather than N & %. P values for categorical variables were calculated using two-sided chi-square tests and p values for continuous variables were calculated using two-sided t-tests

Supplementary table S14: Secondary analyses: Baseline characteristics of the 6-months cohort for those referred to the NHS DPP and completed the programme (intervention group) compared to those not referred to the NHS DPP (control group), by age

	**<70 years**					**70 years and over**				
	N		%			N		%		
	Referred to the NHS DPP and completed	Referred to the NHS DPP and completed	Referred to the NHS DPP and completed	Referred to the NHS DPP and completed	P value	Referred to the NHS DPP and completed	Referred to the NHS DPP and completed	Referred to the NHS DPP and completed	Referred to the NHS DPP and completed	P value
Total	32,198	33,453	100%	100%		24,231	23,487	100%	100%	
Number of LTC_Ls										
0	20,119	19,766	62.5%	59.1%		8,182	8,613	33.8%	36.7%	
At least 1	12,079	13,687	37.5%	40.9%	<0.001	16,049	14,874	66.2%	63.3%	<0.001
At least 2	4,722	5,531	14.7%	16.5%	<0.001	9,469	8,166	39.1%	34.8%	<0.001
At least 3	1,603	1,898	5.0%	5.7%	<0.001	4,687	3,736	19.3%	15.9%	<0.001
At least 4	520	581	1.6%	1.7%	0.225	2,147	1,553	8.9%	6.6%	<0.001
Number of LTC_PLs										
0	24,702	25,258	76.7%	75.5%		15,335	15,343	63.3%	65.3%	
At least 1	7,496	8,195	23.3%	24.5%	<0.001	8,896	8,144	36.7%	34.7%	<0.001
At least 2	1,930	2,084	6.0%	6.2%	<0.001	2,606	2,284	10.8%	9.7%	<0.001
At least 3	498	548	1.5%	1.6%	<0.001	803	631	3.3%	2.7%	<0.001
At least 4	134	137	0.4%	0.4%	0.225	195	138	0.8%	0.6%	<0.001
Number of LTCs										
0	17,475	17,014	54.3%	50.9%		6,867	7,137	28.3%	30.4%	
At least 1	14,723	16,439	45.7%	49.1%	<0.001	17,364	16,350	71.7%	69.6%	<0.001
At least 2	7,816	8,912	24.3%	26.6%	<0.001	12,411	11,192	51.2%	47.7%	<0.001
At least 3	3,661	4,298	11.4%	12.8%	<0.001	7,567	6,367	31.2%	27.1%	<0.001
At least 4	1,676	1,883	5.2%	5.6%	0.017	4,342	3,439	17.9%	14.6%	<0.001
**LTC_L**										

	<70 years					70 years and over				
	N		%			N		%		
	Referred to the NHS DPP and completed	Referred to the NHS DPP and completed	Referred to the NHS DPP and completed	Referred to the NHS DPP and completed	P value	Referred to the NHS DPP and completed	Referred to the NHS DPP and completed	Referred to the NHS DPP and completed	Referred to the NHS DPP and completed	P value
Atrial Fibrillation	813	1,157	2.5%	3.5%	<0.001	3,171	2,737	13.1%	11.7%	<0.001
Cancer	1,691	1,947	5.3%	5.8%	0.001	3,019	2,607	12.5%	11.1%	<0.001
Cerebrovascular Disease	604	802	1.9%	2.4%	<0.001	1,423	1,159	5.9%	4.9%	<0.001
Chronic Kidney Disease	273	318	0.8%	1.0%	0.164	1,106	915	4.6%	3.9%	<0.001
Chronic Liver Disease	190	180	0.6%	0.5%	0.373	117	102	0.5%	0.4%	0.433
Coronary Heart Disease	2,702	3,111	8.4%	9.3%	<0.001	4,912	4,619	20.3%	19.7%	0.098
Dementia	70	133	0.2%	0.4%	<0.001	652	299	2.7%	1.3%	<0.001
Depression	3,347	3,223	10.4%	9.6%	<0.001	1,274	1,170	5.3%	5.0%	0.171
Frailty	136	142	0.4%	0.4%	0.967	1,057	488	4.4%	2.1%	<0.001
Heart Failure	529	570	1.6%	1.7%	0.543	1,290	1,013	5.3%	4.3%	<0.001
Hypertension	7,476	8,715	23.2%	26.1%	<0.001	12,502	11,509	51.6%	49.0%	<0.001
Osteoporosis	456	699	1.4%	2.1%	<0.001	1,763	1,324	7.3%	5.6%	<0.001
Peripheral Vascular Disease	657	738	2.0%	2.2%	0.141	1,260	1,032	5.2%	4.4%	<0.001
Physical Disability	222	231	0.7%	0.7%	0.987	232	178	1.0%	0.8%	0.018
**LTC_PL**										
Alcohol Dependence	187	137	0.6%	0.4%	0.002	41	56	0.2%	0.2%	0.093
Asthma	2,937	3,038	9.1%	9.1%	0.858	2,356	2,265	9.7%	9.6%	0.769
Bronchiectasis	136	163	0.4%	0.5%	0.217	340	305	1.4%	1.3%	0.323
Chronic Pain	800	894	2.5%	2.7%	0.129	757	618	3.1%	2.6%	0.001
COPD	844	862	2.6%	2.6%	0.72	1,455	1,263	6.0%	5.4%	0.003
Epilepsy	265	276	0.8%	0.8%	0.977	218	149	0.9%	0.6%	0.001
Inflammatory Bowel Disease	386	399	1.2%	1.2%	0.943	286	283	1.2%	1.2%	0.804
Multiple Sclerosis	83	102	0.3%	0.3%	0.255	50	25	0.2%	0.1%	0.006

	<70 years					70 years and over				
	N		%			N		%		
	Referred to the NHS DPP and completed	Referred to the NHS DPP and completed	Referred to the NHS DPP and completed	Referred to the NHS DPP and completed	P value	Referred to the NHS DPP and completed	Referred to the NHS DPP and completed	Referred to the NHS DPP and completed	Referred to the NHS DPP and completed	P value
Neurological Organ Failure	21	14	0.1%	0.0%	0.195	58	26	0.2%	0.1%	0.001
Osteoarthritis	3,167	3,780	9.8%	11.3%	<0.001	5,664	5,168	23.4%	22.0%	<0.001
Parkinson's Disease	35	48	0.1%	0.1%	0.21	107	72	0.4%	0.3%	0.016
Pulmonary Heart Disease	252	292	0.8%	0.9%	0.202	450	384	1.9%	1.6%	0.064
Rheumatoid Arthritis	332	370	1.0%	1.1%	0.351	471	375	1.9%	1.6%	0.004
Sarcoidosis	70	77	0.2%	0.2%	0.729	30	32	0.1%	0.1%	0.706
Serious Mental Illness	557	515	1.7%	1.5%	0.054	191	127	0.8%	0.5%	0.001
Severe Interstitial Lung Disease	34	42	0.1%	0.1%	0.452	102	92	0.4%	0.4%	0.616
Age~	56.0	59.3	9.8	8.3	<0.001	78.3	75.5	6.1	4.5	<0.001
Sex										
Female	16,378	18,805	50.9%	56.2%	<0.001	14,300	12,338	59.0%	52.5%	<0.001
Male	15,820	14,648	49.1%	43.8%		9,931	11,149	41.0%	47.5%	
Ethnicity										
Asian	3,289	3,372	10.2%	10.1%	<0.001	698	569	2.9%	2.4%	0.005
Black	2,272	2,404	7.1%	7.2%		436	455	1.8%	1.9%	
Mixed	551	576	1.7%	1.7%		169	142	0.7%	0.6%	
Other	1,113	1,050	3.5%	3.1%		315	263	1.3%	1.1%	
Unknown	2,755	2,560	8.6%	7.7%		1,136	1,070	4.7%	4.6%	
White	22,218	23,491	69.0%	70.2%		21,477	20,988	88.6%	89.4%	
IMD quintile										
1 (most deprived)	5,580	5,360	17.3%	16.0%	<0.001	2,430	2,167	10.0%	9.2%	0.038
2	6,118	6,396	19.0%	19.1%		3,555	3,483	14.7%	14.8%	
3	6,721	6,971	20.9%	20.8%		5,019	4,844	20.7%	20.6%	

	<70 years					70 years and over				
	N		%			N		%		
	Referred to the NHS DPP and completed	Referred to the NHS DPP and completed	Referred to the NHS DPP and completed	Referred to the NHS DPP and completed	P value	Referred to the NHS DPP and completed	Referred to the NHS DPP and completed	Referred to the NHS DPP and completed	Referred to the NHS DPP and completed	P value
4	6,910	7,237	21.5%	21.6%		5,988	5,951	24.7%	25.3%	
5 (least deprived)	6,869	7,489	21.3%	22.4%		7,239	7,042	29.9%	30.0%	
Rural GP										
No	28,961	30,138	89.9%	90.1%	0.538	20,735	20,375	85.6%	86.8%	<0.001
Yes	3,237	3,315	10.1%	9.9%		3,496	3,112	14.4%	13.2%	
IMD quintile GP										
1 (most deprived)	7,213	7,769	22.4%	23.2%	<0.001	7,242	7,262	29.9%	30.9%	<0.001
2	7,036	6,982	21.9%	20.9%		6,167	5,553	25.5%	23.6%	
3	6,324	6,787	19.6%	20.3%		4,728	4,610	19.5%	19.6%	
4	6,107	6,591	19.0%	19.7%		3,651	3,852	15.1%	16.4%	
5 (least deprived)	5,518	5,324	17.1%	15.9%		2,443	2,210	10.1%	9.4%	
QOF overall quintile GP										
1 (Highest achievement)	5,884	5,549	18.3%	16.6%	<0.001	3,725	3,403	15.4%	14.5%	<0.001
2	6,183	6,367	19.2%	19.0%		3,916	4,101	16.2%	17.5%	
3	6,525	7,259	20.3%	21.7%		4,765	4,856	19.7%	20.7%	
4	6,723	6,970	20.9%	20.8%		5,779	5,376	23.8%	22.9%	
5 (Lowest achievement)	6,883	7,308	21.4%	21.8%		6,046	5,751	25.0%	24.5%	
size quintile GP										
1 (Smallest)	6,096	6,543	18.9%	19.6%	0.047	3,949	3,867	16.3%	16.5%	0.232
2	6,350	6,719	19.7%	20.1%		4,750	4,548	19.6%	19.4%	
3	6,489	6,760	20.2%	20.2%		4,809	4,840	19.8%	20.6%	
4	6,511	6,667	20.2%	19.9%		5,374	5,087	22.2%	21.7%	
5 (Largest)	6,752	6,764	21.0%	20.2%		5,349	5,145	22.1%	21.9%	

	<70 years					70 years and over				
	N		%			N		%		
	Referred to the NHS DPP and completed	Referred to the NHS DPP and completed	Referred to the NHS DPP and completed	Referred to the NHS DPP and completed	P value	Referred to the NHS DPP and completed	Referred to the NHS DPP and completed	Referred to the NHS DPP and completed	Referred to the NHS DPP and completed	P value
FTE quintile GP										
1 (Least GPs per patient)	6,049	6,196	18.8%	18.5%	<0.001	3,543	3,390	14.6%	14.4%	0.01
2	6,422	7,172	19.9%	21.4%		4,578	4,742	18.9%	20.2%	
3	6,719	7,014	20.9%	21.0%		5,340	5,148	22.0%	21.9%	
4	6,711	6,879	20.8%	20.6%		5,462	5,193	22.5%	22.1%	
5 (Most GPs per patient)	6,297	6,192	19.6%	18.5%		5,308	5,014	21.9%	21.3%	
Arrivals~	0.3	0.3	0.7	0.8	0.5503	0.3	0.3	0.7	0.8	0.6724
Admissions~	0.3	0.3	1.1	1.0	0.2702	0.5	0.5	1.1	1.1	0.337
Appointments~	2.6	2.9	5.6	5.4	<0.001	3.9	3.8	6.1	5.6	0.7496

~ Mean & SD reported rather than N & %. P values for categorical variables were calculated using two-sided chi-square tests and p values for continuous variables were calculated using two-sided t-tests

Supplementary table S15: Secondary analyses : Unadjusted and adjusted estimates of incidence of type 2 diabetes, LTCs considered aetiologically linked to diet, physical activity and body weight (LTC-L) and LTCs possibly linked to diet, physical activity and body weight (LTC-PL) at 6,12,18 and 24 months follow-up for those referred to the NHS DPP and completed the programme (intervention group) compared to those not referred to the NHS DPP (control group)

					Odds ratio / rate ratio	
			Control	Intervention	Unadjusted	Adjusted
Type 2 diabetes	6 months	Total	56,429	56,940		
		New diagnoses	537	394		
		%	1.0%	0.7%	0.73 (0.64-0.83) <0.001	0.75 (0.66-0.86) <0.001
	12 months	Total	37,342	38,140		
		New diagnoses	946	703		
		%	2.5%	1.8%	0.72 (0.66-0.8) <0.001	0.74 (0.67-0.82) <0.001
	18 months	Total	22,380	22,497		
		New diagnoses	942	686		
		%	4.2%	3.0%	0.72 (0.65-0.79) <0.001	0.75 (0.67-0.83) <0.001
	24 months	Total	11,509	11,821		
		New diagnoses	668	562		
		%	5.8%	4.8%	0.81 (0.72-0.91) <0.001	0.83 (0.74-0.94) 0.003
Aetiologically linked	6 months	Total	56,429	56,940		
		New diagnoses	5,034	2,999		
		%	8.9%	5.3%	0.59 (0.56-0.62) <0.001	0.64 (0.6-0.67) <0.001
	12 months	Total	37,342	38,140		
		New diagnoses	6,519	4,474		
		%	17.5%	11.7%	0.67 (0.64-0.71) <0.001	0.72 (0.69-0.76) <0.001
	18 months	Total	22,380	22,497		
		New diagnoses	5,527	4,106		
		%	24.7%	18.3%	0.74 (0.7-0.78) <0.001	0.78 (0.74-0.82) <0.001
	24 months	Total	11,509	11,821		

			Control	Intervention	Odds ratio / rate ratio<br>Unadjusted	Adjusted
		New diagnoses	3,720	2,952		
		%	32.3%	25.0%	0.77 (0.72-0.82) <0.001	0.79 (0.75-0.85) <0.001
Possibly linked	6 months	Total	56,429	56,940		
		New diagnoses	1,657	1,059		
		%	2.9%	1.9%	0.63 (0.58-0.69) <0.001	0.64 (0.59-0.7) <0.001
	12 months	Total	37,342	38,140		
		New diagnoses	2,214	1,611		
		%	5.9%	4.2%	0.71 (0.66-0.77) <0.001	0.72 (0.67-0.77) <0.001
	18 months	Total	22,380	22,497		
		New diagnoses	1,978	1,540		
		%	8.8%	6.8%	0.77 (0.72-0.84) <0.001	0.78 (0.72-0.84) <0.001
	24 months	Total	11,509	11,821		
		New diagnoses	1,410	1,136		
		%	12.3%	9.6%	0.78 (0.72-0.86) <0.001	0.79 (0.72-0.87) <0.001

Data are presented as odds ratios (diabetes) and rate ratios (LTC-L and LTC-PL) with 95% confidence intervals. P values are two-sided and are determined by logistic regression (diabetes) and negative binomial regression (LTC-L and LTC-PL), with no adjustments for multiple tests.

Supplementary table S16: Secondary analyses: Number of individuals who were living with MLTC at the start of the follow-up period and number who developed MLTC at follow-up for those referred to the NHS DPP and completed the programme (intervention group) compared to those not referred to the NHS DPP (control group)

	6 months			12 months			18 months			24 months		
	Control	Intervention	P value*	Control	Intervention	P value*	Control	Intervention	P value*	Control	Intervention	P value*
N	56,429	56,940		37,342	38,140		22,380	22,497		11,509	11,821	
Living with MLTC	20,227	20,104		13,250	13,347		7,715	7,767		3,866	4,055	
No MLTC	36,202	36,836		24,092	24,793		14,665	14,730		7,643	7,766	
% Living with MLTC	35.8%	35.3%	0.059	35.5%	35.0%	0.160	34.5%	34.5%	0.908	33.6%	34.3%	0.251
MLTC at follow-up	1,501	1,039		1,988	1,557		1,719	1,464		1,224	1,042	
No MLTC at follow-up	34,701	35,797		22,104	23,236		12,946	13,266		6,419	6,724	
% developed MLTC at follow-up	4.1%	2.8%	<0.001	8.3%	6.3%	<0.001	11.7%	9.9%	<0.001	16.0%	13.4%	<0.001

*P value for the percentage of individuals living with MLTC at baseline calculated using a two-sided chi-square test and p value for individuals who developed MLTC at follow-up calculated using logistic regression

Supplementary table S17: Datasets used to derive LTC variables in the Brides to Health Segmentation Dataset

Dataset	Datasets used to derive the Segmentation Dataset	Time periods for data
Master Patient Index (MPI)	Master Patient Index data for all people registered to a GP practice in England.	Aug 14 - Jun 21
Secondary Use Services (SUS)	Admitted patient care (APC), outpatient attendances (OPA) and A&E data from secondary care. Hospital Frailty Risk Score (HFRS) data derived from APC data.	APC/OPA: Apr 08 - Jun 21<br>A&E: Apr 08 – Mar 20<br>HFRS: Apr 14 – Jun 21
Emergency Care Dataset (ECDS)	Urgent and emergency care (A&E) attendances and admissions (a part of SUS). A new dataset that has replaced the existing A&E SUS dataset.	Oct 17 – Jun 21
Community Services Dataset (CSDS)	Data for community services provided by community trusts, acute trusts, mental health trusts, care trusts, integrated care organisations and other qualified providers.	Oct 17 – Jun 21
Assuring Transformation Dataset (AT)	Inpatient data on people who have a learning disability and/or autism from NHS and independent sector mental or behavioural healthcare providers.	Feb 15 – Jun 21
Service-level Agreement Monitoring (SLAM)	Financial activity data attached to specialised or tertiary services.	Apr 16 – Jun 21
Mental Health Services Datasets (MHSDS)	Activity related to specialist mental health or learning disability services, including historical versions of this dataset (Mental Health Minimum Data Set (MHMDS); Mental Health and Learning Disabilities Data Set (MHLDDS); and Mental Health Services Datasets (MHSDS) v1-4).	Apr 13 – Jun 21
Improving Access to Psychological Therapies Data Set (IAPT)	Data from the Adult Improving Access to Psychological Therapies (IAPT) programme for the treatment of anxiety disorders and depression in adults in England.	Apr 13 – Jun 21
Maternity Services Data Set (MSDS)	Maternity Services Data Set (MSDS) v2.0 capturing data on maternity services from the point of the first booking appointment until discharge from services.	Apr 19 – Jun 21
National Diabetes Audit (NDA)	Data related to care received by people with diabetes from all GP practices registered as part of the audit.	Jan 14 – Mar 21

Supplementary Figures

Supplementary Figure S1: Exclusion criteria and frequency of exclusions at different stages in the study

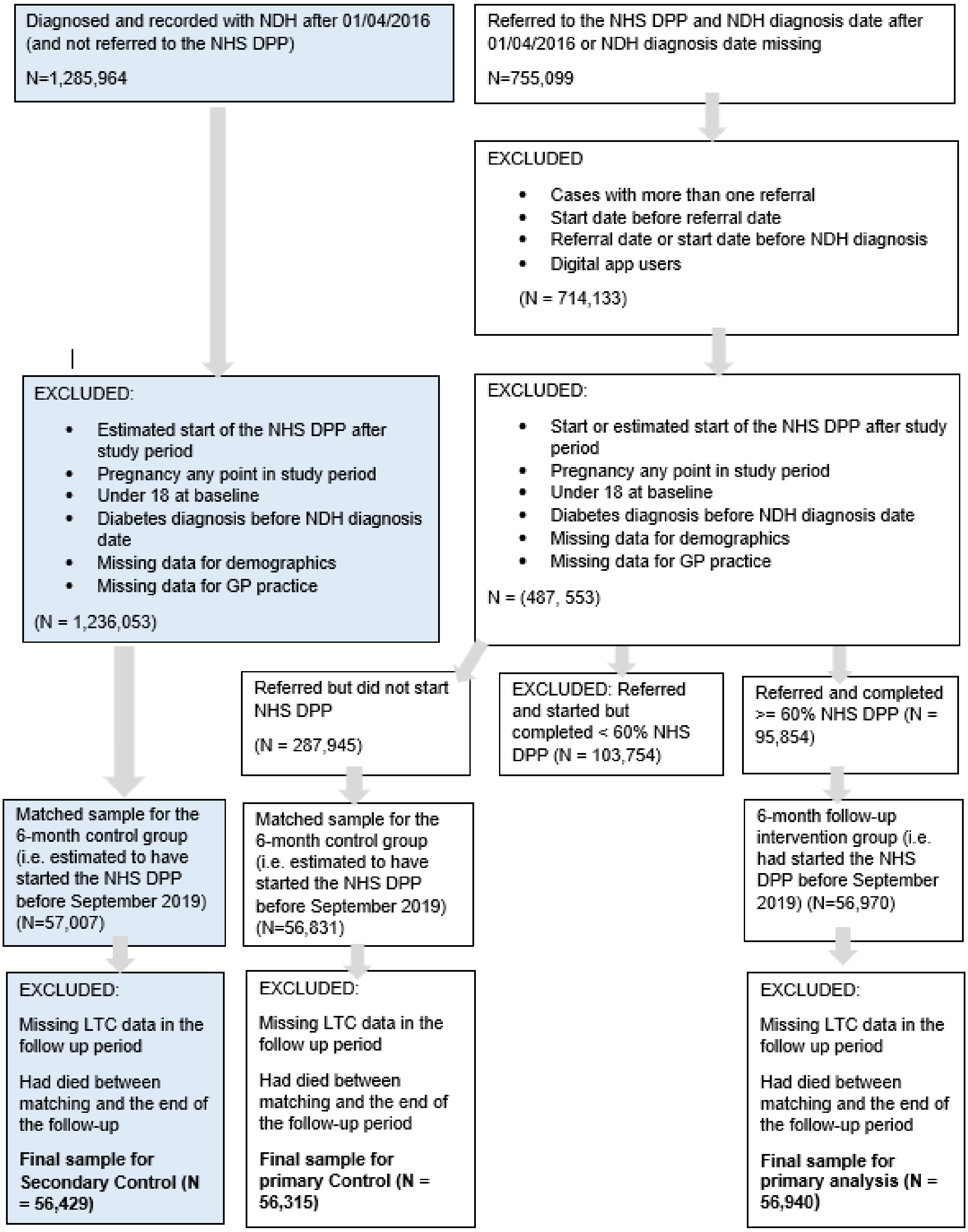

Supplementary figure S2: Standardised Mean Differences

6 months

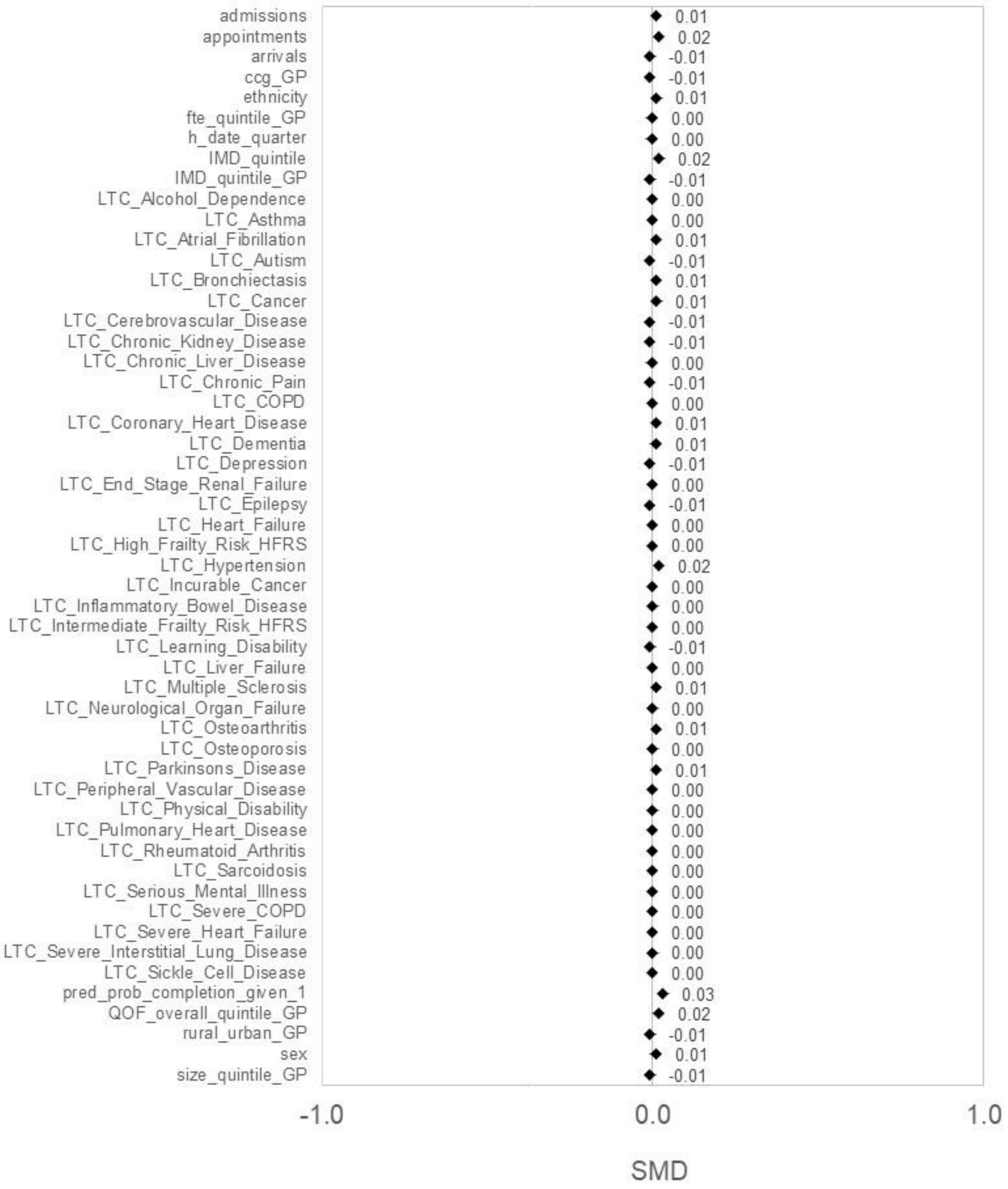

12 months

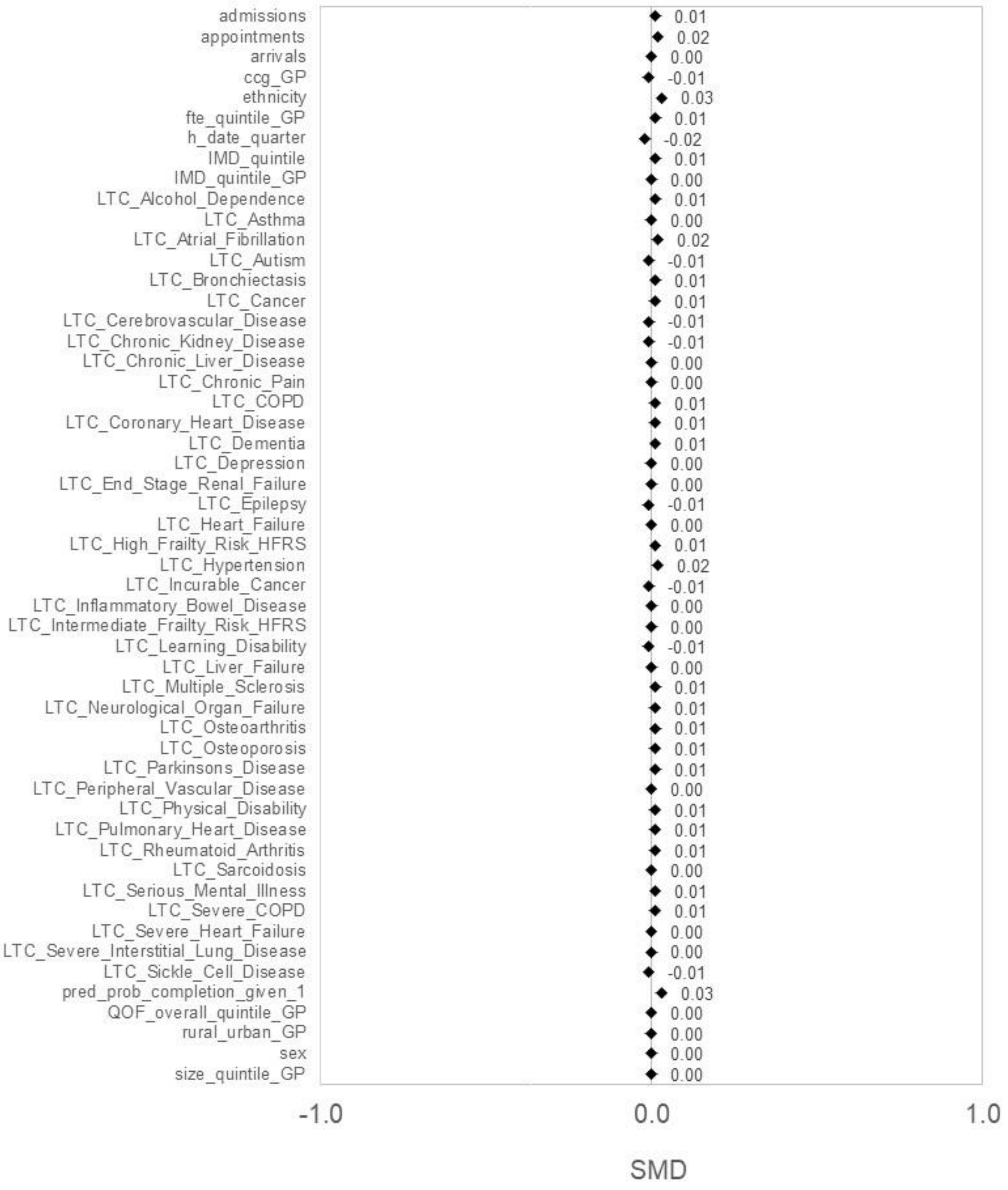
admissions 0.01
appointments 0.02
arrivals 0.00
ccg_GP -0.01
ethnicity 0.03
fte_quintile_GP 0.01
h_date_quarter -0.02
IMD_quintile 0.01
IMD_quintile_GP 0.00
LTC_Alcohol_Dependence 0.01
LTC_Asthma 0.00
LTC_Atrial_Fibrillation 0.02
LTC_Autism -0.01
LTC_Bronchiectasis 0.01
LTC_Cancer 0.01
LTC_Cerebrovascular_Disease -0.01
LTC_Chronic_Kidney_Disease -0.01
LTC_Chronic_Liver_Disease 0.00
LTC_Chronic_Pain 0.00
LTC_COPD 0.01
LTC_Coronary_Heart_Disease 0.01
LTC_Dementia 0.01
LTC_Depression 0.00
LTC_End_Stage_Renal_Failure 0.00
LTC_Epilepsy -0.01
LTC_Heart_Failure 0.00
LTC_High_Frailty_Risk_HFRS 0.01
LTC_Hypertension 0.02
LTC_Incurable_Cancer -0.01
LTC_Inflammatory_Bowel_Disease 0.00
LTC_Intermediate_Frailty_Risk_HFRS 0.00
LTC_Learning_Disability -0.01
LTC_Liver_Failure 0.00
LTC_Multiple_Sclerosis 0.01
LTC_Neurological_Organ_Failure 0.01
LTC_Osteoarthritis 0.01
LTC_Osteoporosis 0.01
LTC_Parkinsons_Disease 0.01
LTC_Peripheral_Vascular_Disease 0.00
LTC_Physical_Disability 0.01
LTC_Pulmonary_Heart_Disease 0.01
LTC_Rheumatoid_Arthritis 0.01
LTC_Sarcoidosis 0.00
LTC_Serious_Mental_Illness 0.01
LTC_Severe_COPD 0.01
LTC_Severe_Heart_Failure 0.00
LTC_Severe_Interstitial_Lung_Disease 0.00
LTC_Sickle_Cell_Disease -0.01
pred_prob_completion_given_1 0.03
QOF_overall_quintile_GP 0.00
rural_urban_GP 0.00
sex 0.00
size_quintile_GP 0.00
-1.0
0.0
1.0
SMD

18 months

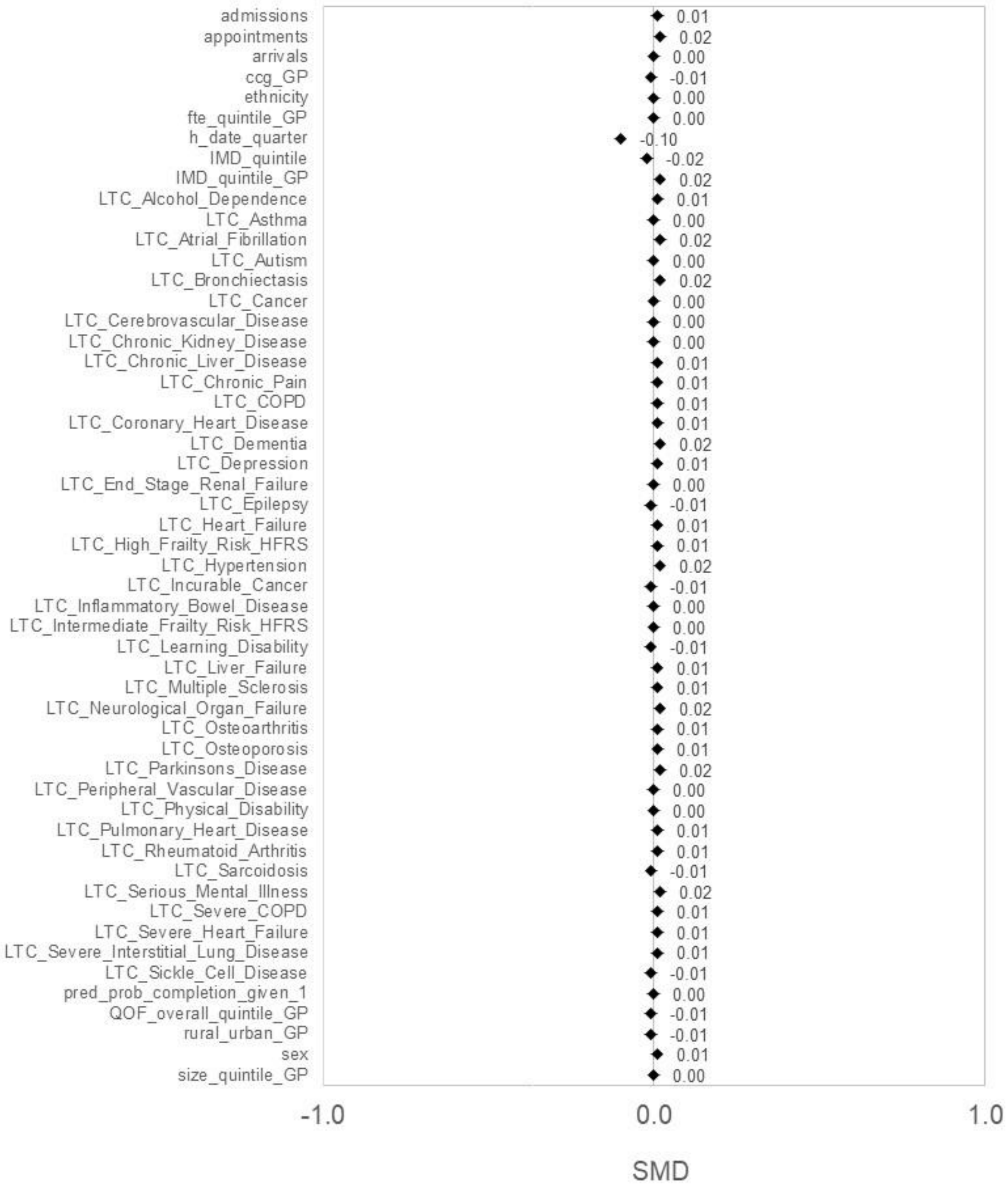
admissions
appointments
arrivals
ccg_GP
ethnicity
fte_quintile_GP
h_date_quarter
IMD_quintile
IMD_quintile_GP
LTC_Alcohol_Dependence
LTC_Asthma
LTC_Atrial_Fibrillation
LTC_Autism
LTC_Bronchiectasis
LTC_Cancer
LTC_Cerebrovascular_Disease
LTC_Chronic_Kidney_Disease
LTC_Chronic_Liver_Disease
LTC_Chronic_Pain
LTC_COPD
LTC_Coronary_Heart_Disease
LTC_Dementia
LTC_Depression
LTC_End_Stage_Renal_Failure
LTC_Epilepsy
LTC_Heart_Failure
LTC_High_Frailty_Risk_HFRS
LTC_Hypertension
LTC_Incurable_Cancer
LTC_Inflammatory_Bowel_Disease
LTC_Intermediate_Frailty_Risk_HFRS
LTC_Learning_Disability
LTC_Liver_Failure
LTC_Multiple_Sclerosis
LTC_Neurological_Organ_Failure
LTC_Osteoarthritis
LTC_Osteoporosis
LTC_Parkinsons_Disease
LTC_Peripheral_Vascular_Disease
LTC_Physical_Disability
LTC_Pulmonary_Heart_Disease
LTC_Rheumatoid_Arthritis
LTC_Sarcoidosis
LTC_Serious_Mental_Illness
LTC_Severe_COPD
LTC_Severe_Heart_Failure
LTC_Severe_Interstitial_Lung_Disease
LTC_Sickle_Cell_Disease
pred_prob_completion_given_1
QOF_overall_quintile_GP
rural_urban_GP
sex
size_quintile_GP
0.01
0.02
0.00
-0.01
0.00
0.00
-0.10
-0.02
0.02
0.01
0.00
0.02
0.00
0.02
0.00
0.00
0.00
0.01
0.01
0.01
0.01
0.02
0.01
0.00
-0.01
0.01
0.01
0.02
-0.01
0.00
0.00
-0.01
0.01
0.01
0.02
0.01
0.01
0.02
0.00
0.00
0.01
0.01
-0.01
0.02
0.01
0.01
0.01
-0.01
0.00
-0.01
-0.01
0.01
0.00
-1.0
0.0
1.0
SMD

24 months

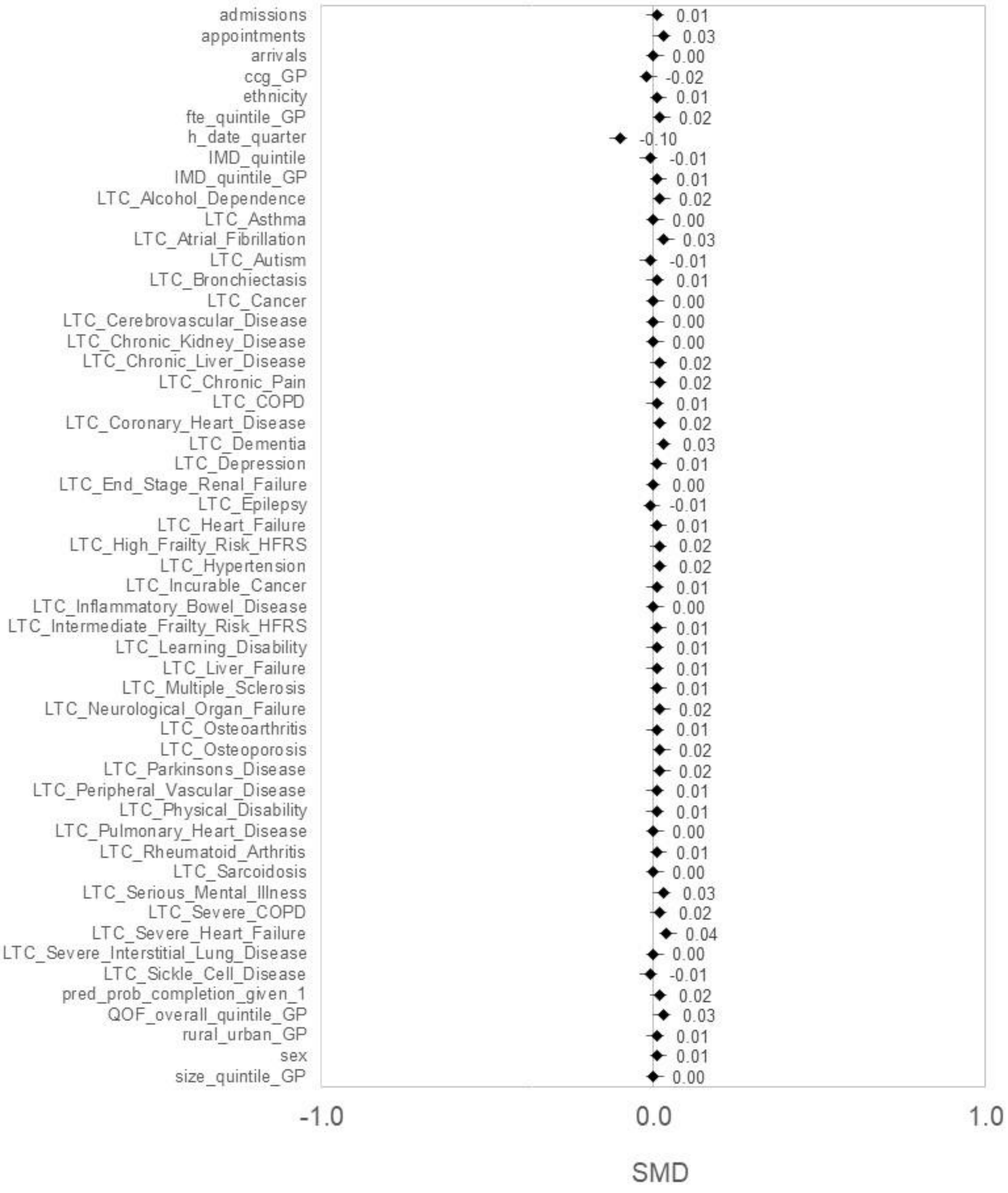
admissions 0.01
appointments 0.03
arrivals 0.00
ccg_GP -0.02
ethnicity 0.01
fte_quintile_GP 0.02
h_date_quarter -0.10
IMD_quintile -0.01
IMD_quintile_GP 0.01
LTC_Alcohol_Dependence 0.02
LTC_Asthma 0.00
LTC_Atrial_Fibrillation 0.03
LTC_Autism -0.01
LTC_Bronchiectasis 0.01
LTC_Cancer 0.00
LTC_Cerebrovascular_Disease 0.00
LTC_Chronic_Kidney_Disease 0.00
LTC_Chronic_Liver_Disease 0.02
LTC_Chronic_Pain 0.02
LTC_COPD 0.01
LTC_Coronary_Heart_Disease 0.02
LTC_Dementia 0.03
LTC_Depression 0.01
LTC_End_Stage_Renal_Failure 0.00
LTC_Epilepsy -0.01
LTC_Heart_Failure 0.01
LTC_High_Frailty_Risk_HFRS 0.02
LTC_Hypertension 0.02
LTC_Incurable_Cancer 0.01
LTC_Inflammatory_Bowel_Disease 0.00
LTC_Intermediate_Frailty_Risk_HFRS 0.01
LTC_Learning_Disability 0.01
LTC_Liver_Failure 0.01
LTC_Multiple_Sclerosis 0.01
LTC_Neurological_Organ_Failure 0.02
LTC_Osteoarthritis 0.01
LTC_Osteoporosis 0.02
LTC_Parkinsons_Disease 0.02
LTC_Peripheral_Vascular_Disease 0.01
LTC_Physical_Disability 0.01
LTC_Pulmonary_Heart_Disease 0.00
LTC_Rheumatoid_Arthritis 0.01
LTC_Sarcoidosis 0.00
LTC_Serious_Mental_Illness 0.03
LTC_Severe_COPD 0.02
LTC_Severe_Heart_Failure 0.04
LTC_Severe_Interstitial_Lung_Disease 0.00
LTC_Sickle_Cell_Disease -0.01
pred_prob_completion_given_1 0.02
QOF_overall_quintile_GP 0.03
rural_urban_GP 0.01
sex 0.01
size_quintile_GP 0.00
-1.0
0.0
1.0
SMD

Men

6 months

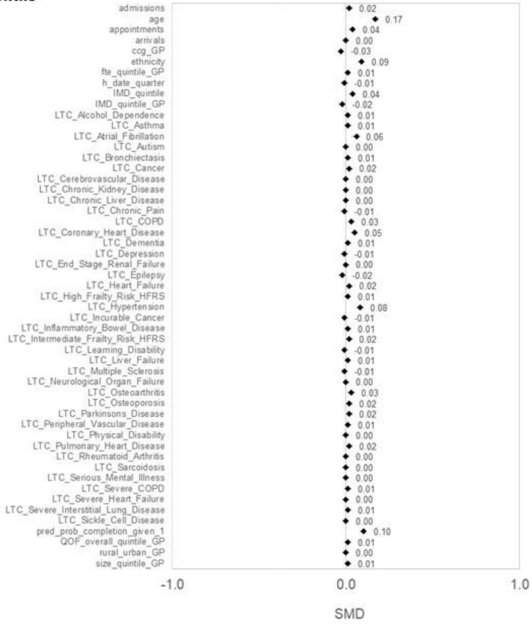

12 months

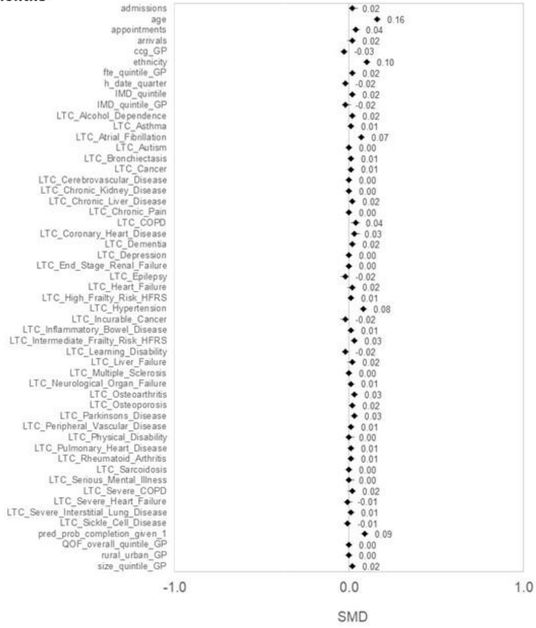

18 months

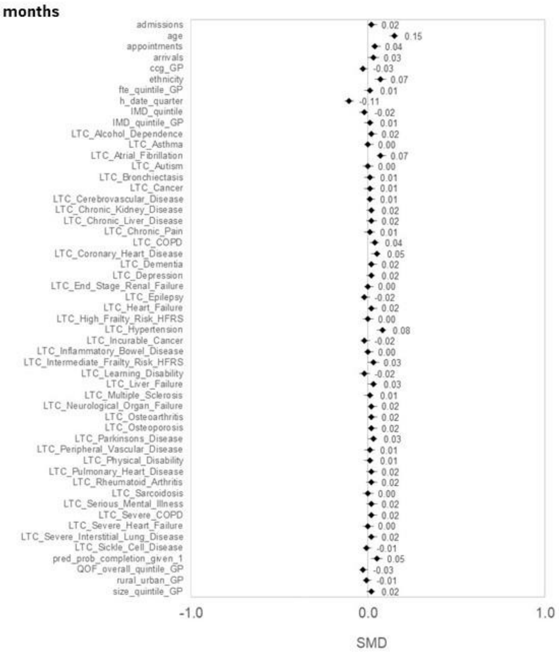

24 months

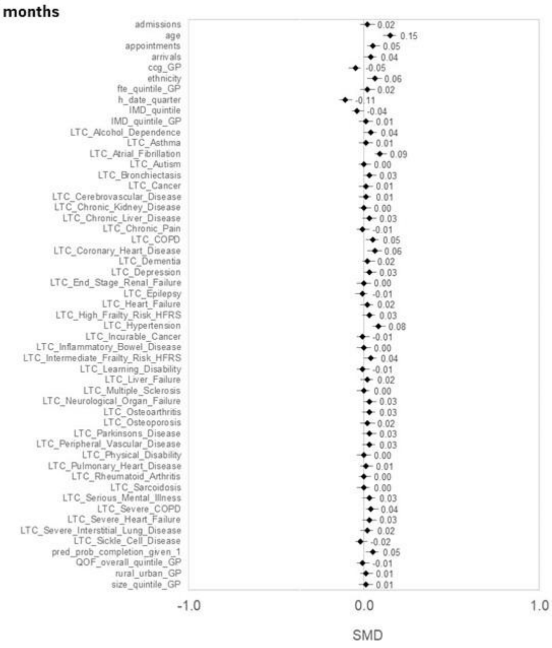

Women

6 months

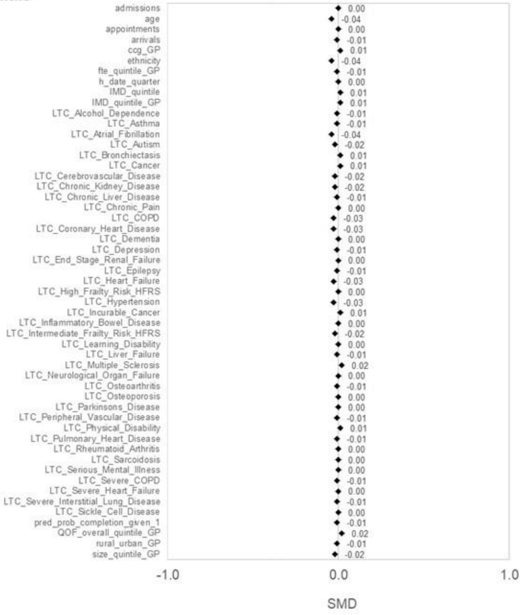

12 months

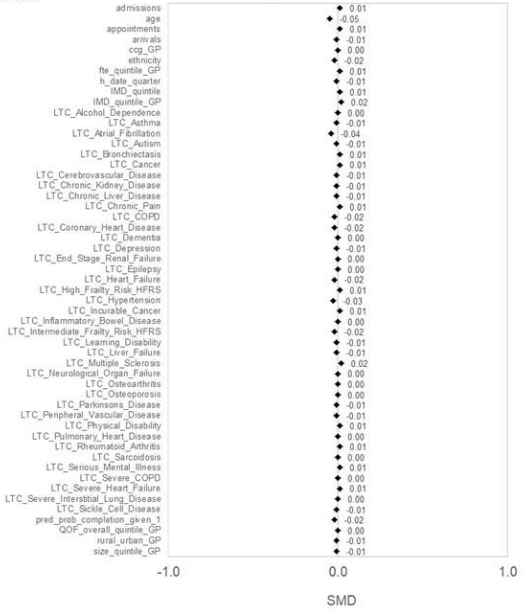

18 months

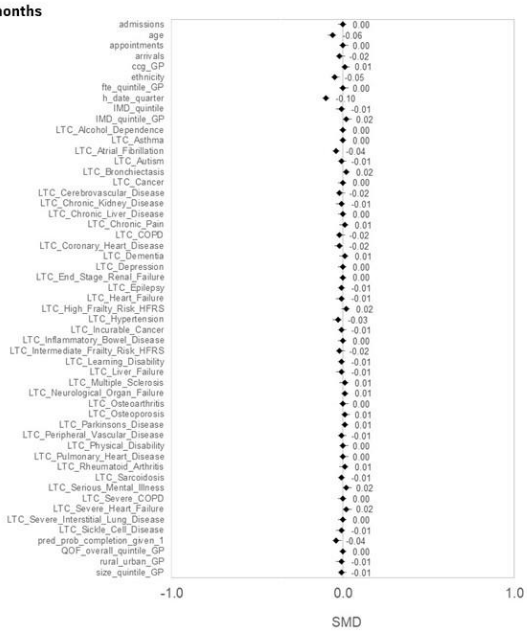

24 months

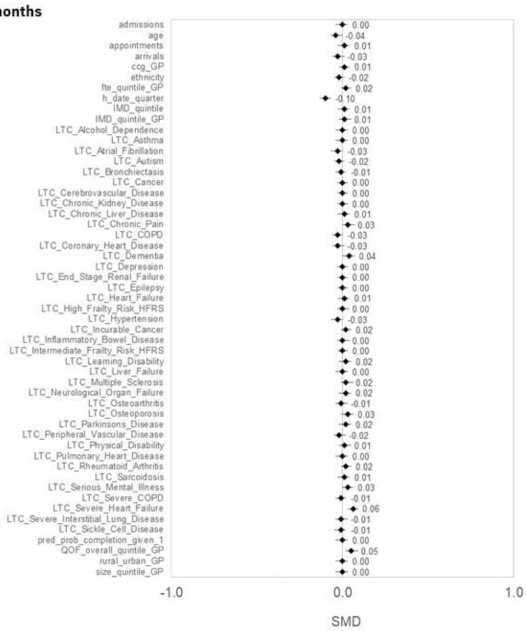

Age under 70 years

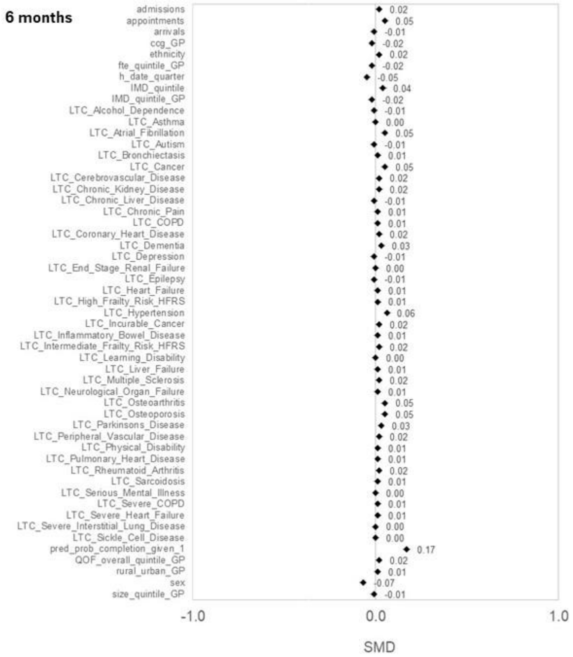

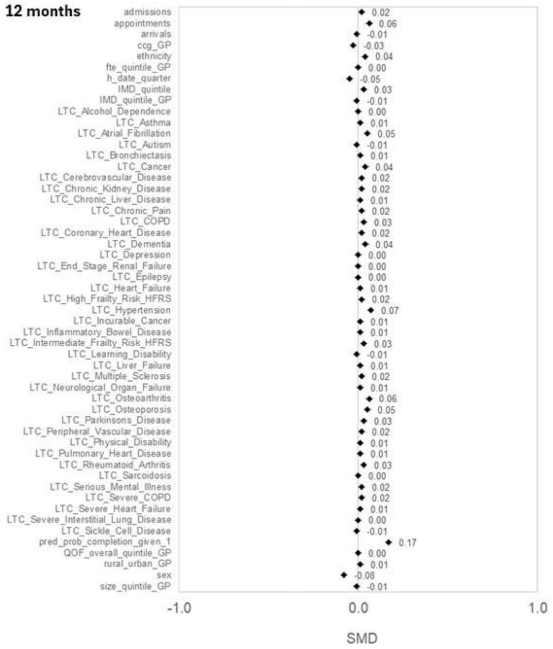

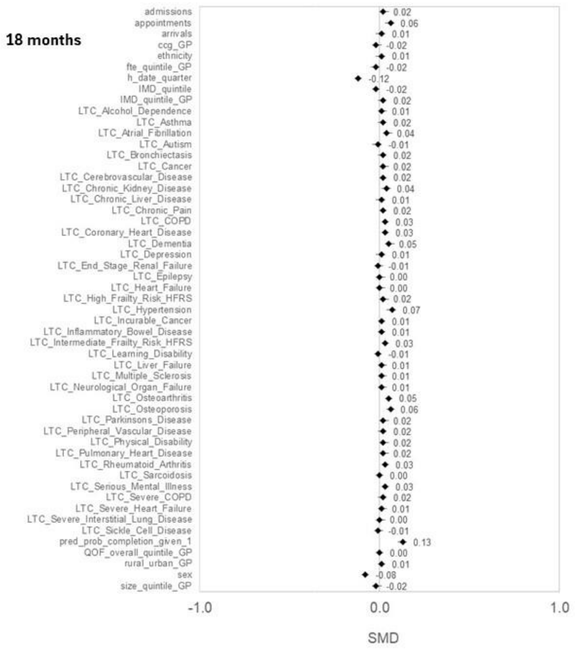

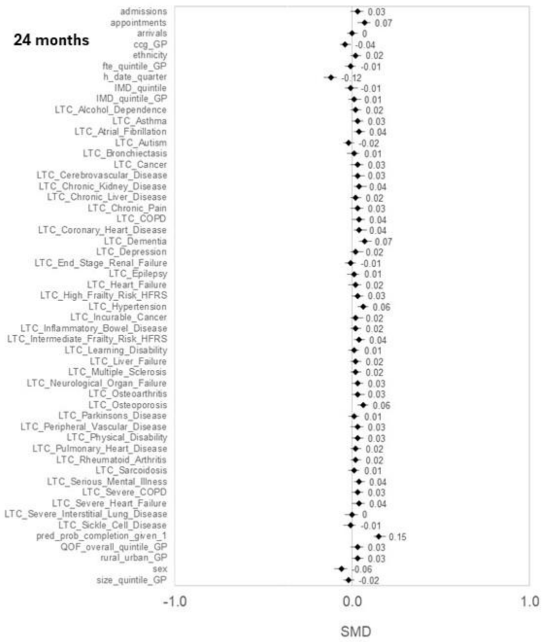

Age 70 years and over

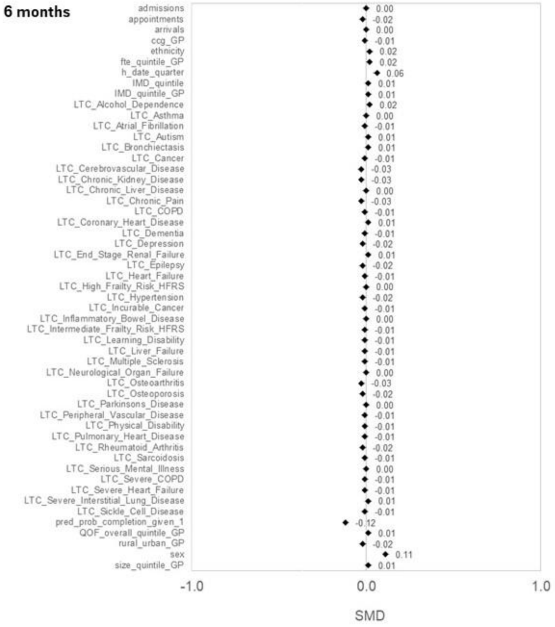

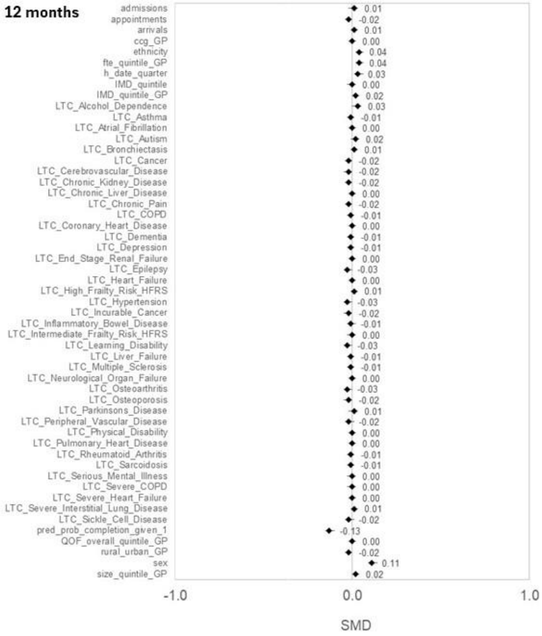

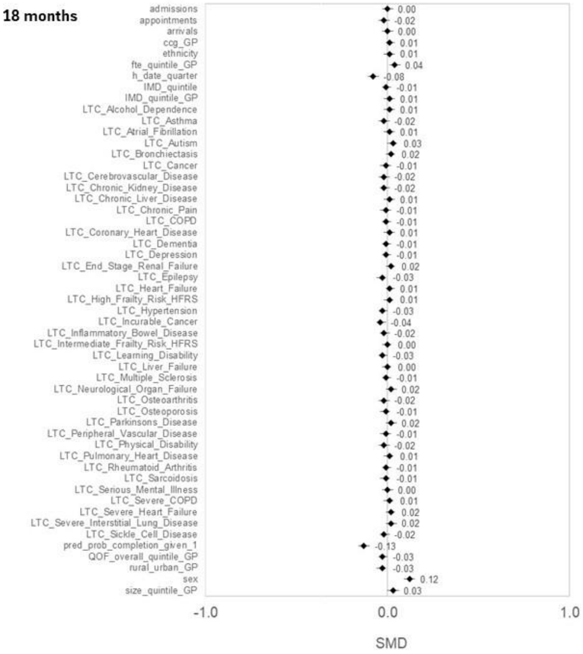

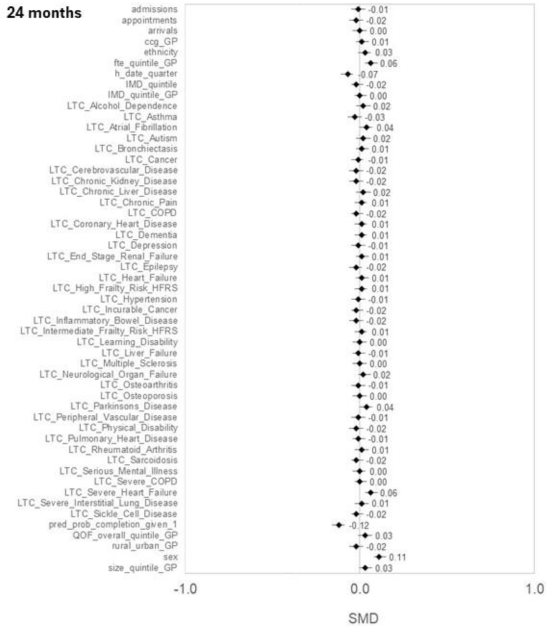

Supplementary Figure 3: Adjusted estimates of incidence of type 2 diabetes and LTC-L and LTC-PL at 6, 12, 18 and 24-month follow-up periods by sex

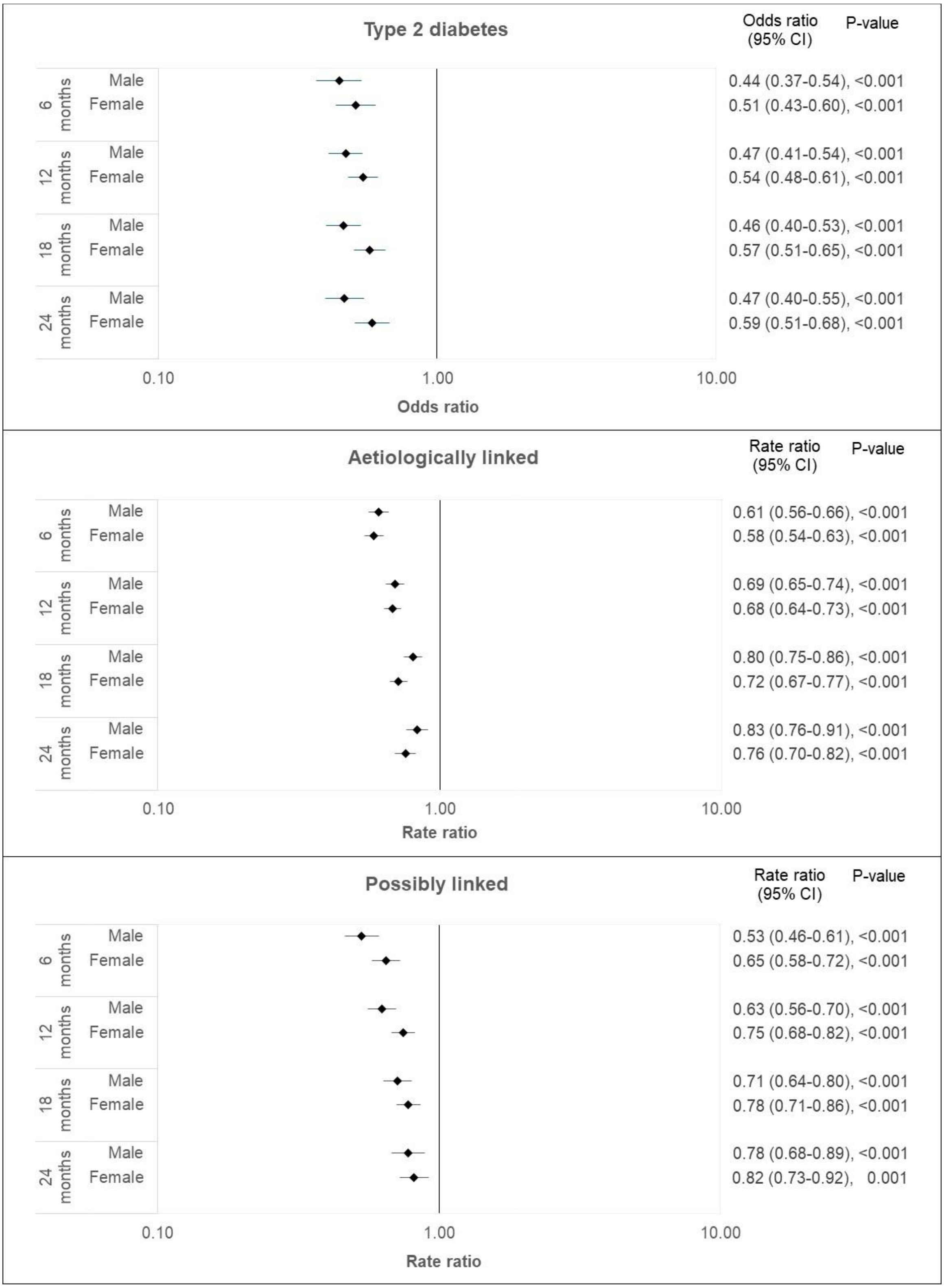

Data are presented as odds ratios (diabetes) and rate ratios (LTC-L and LTC-PL) with 95% confidence intervals. P values are two-sided and are determined by logistic regression (diabetes) and negative binomial regression (LTC-L and LTC-PL), with no adjustments for multiple tests.

Supplementary Figure 4: Adjusted estimates of incidence of type 2 diabetes and LTC-L and LTC-NL at 6, 12, 18 and 24-month follow-up periods by age group

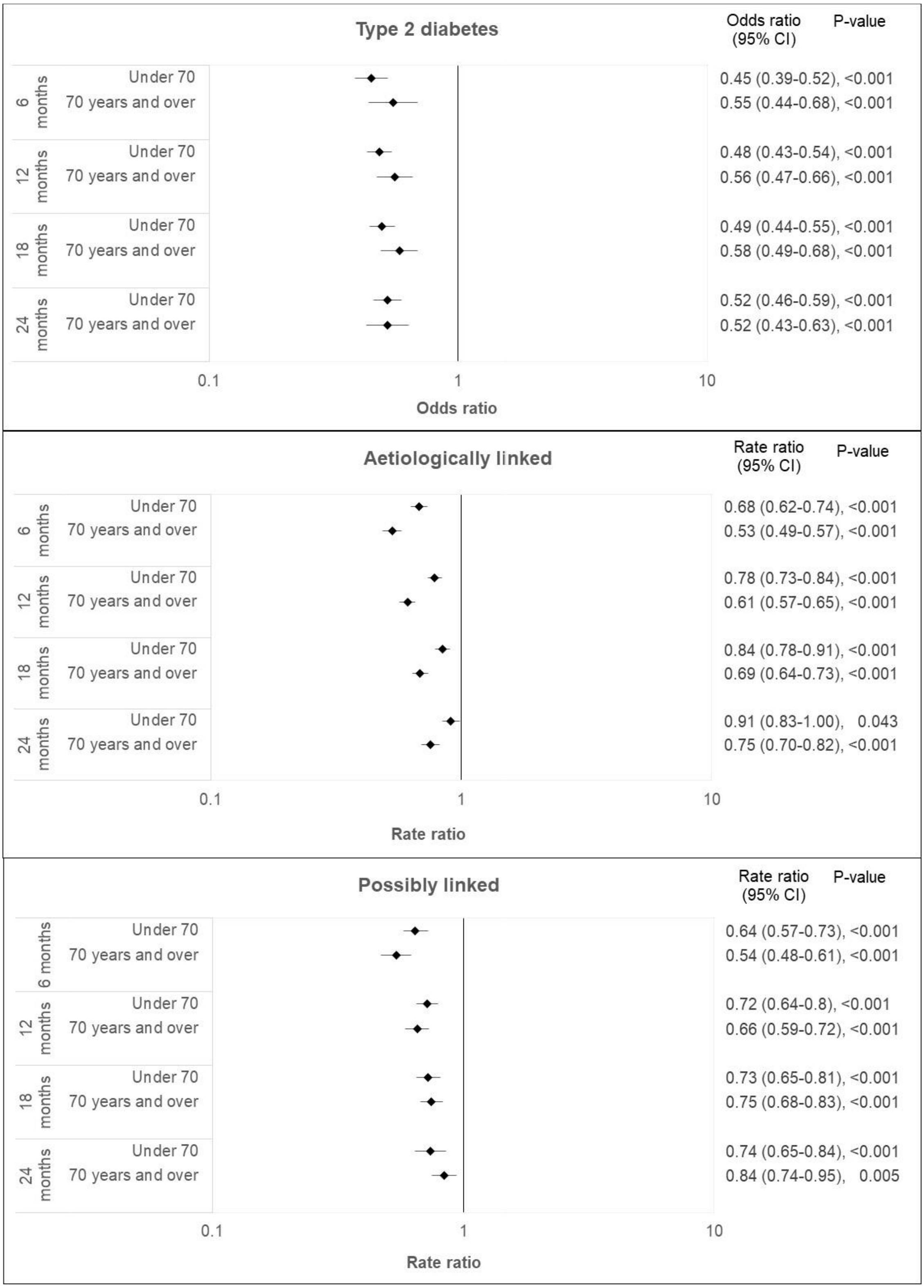

Data are presented as odds ratios (diabetes) and rate ratios (LTC-L and LTC-PL) with 95% confidence intervals. P values are two-sided and are determined by logistic regression (diabetes) and negative binomial regression (LTC-L and LTC-PL), with no adjustments for multiple tests.

Supplementary Figure 5:Odds of participants acquiring new LTCs at 6, 12, 18 and 24-month follow-up periods split by sex

Women

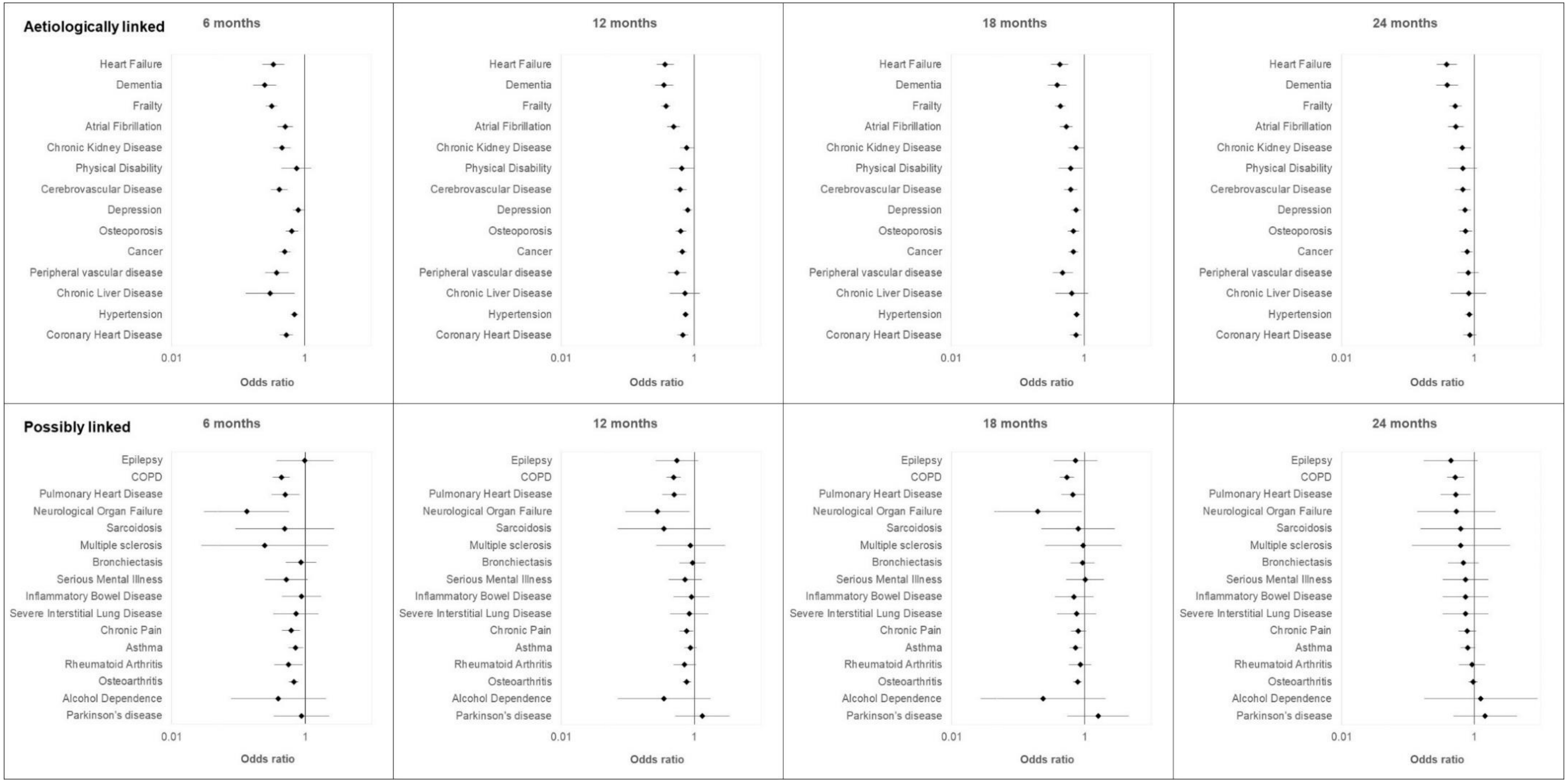

Data are presented as odds ratios with 95% confidence intervals. N (intervention and control) = 31,143 and 30,944 at 6 months, 20,952 and 21,928 at 12 months, 12,332 and 14,663 at 18 months and 6,504 and 8,116 at 24 months

Men

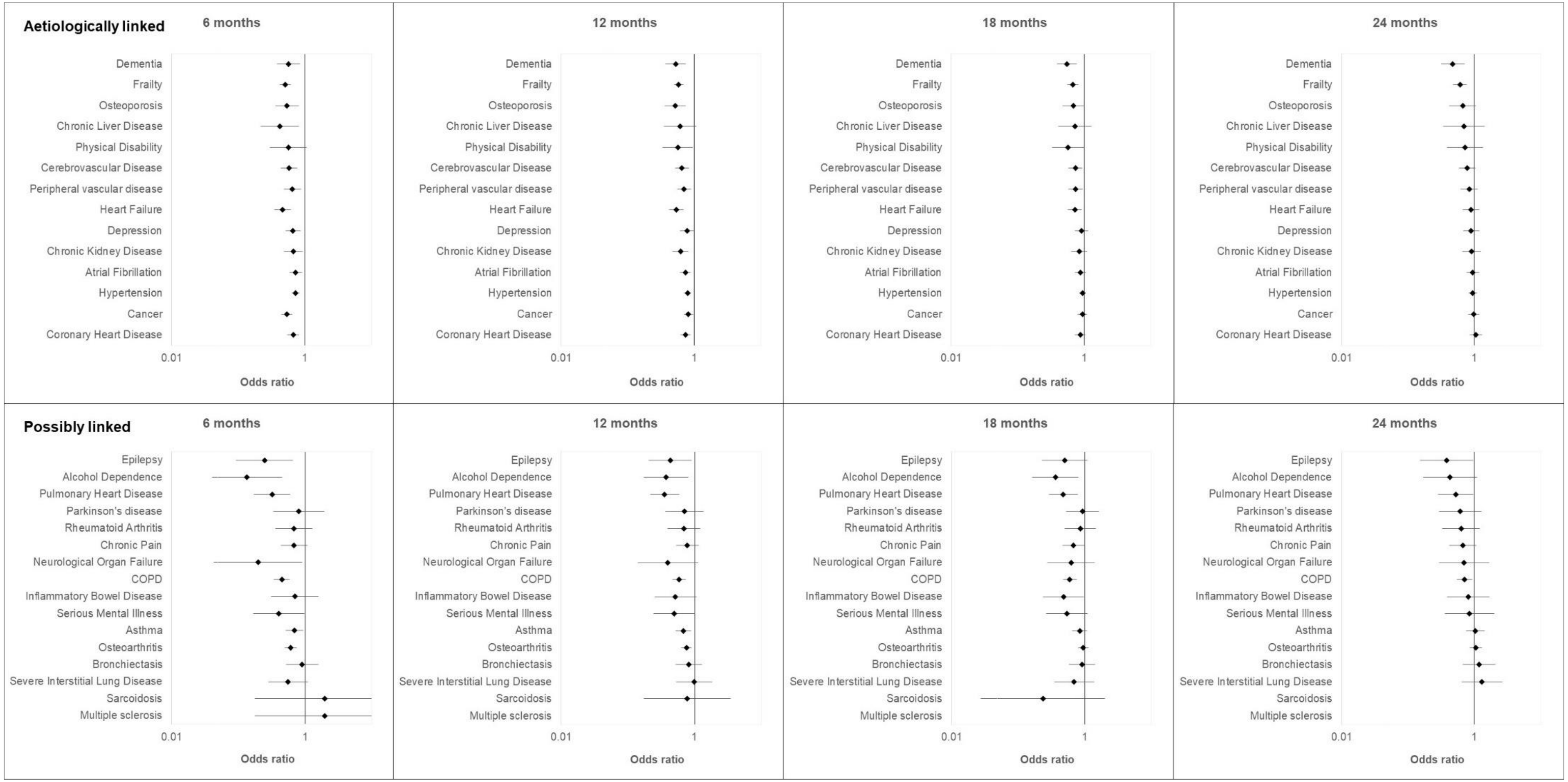

Data are presented as odds ratios with 95% confidence intervals. N (intervention and control) = 25,797 and 25,371 at 6 months, 17,188 and 17,845 at 12 months, 10,165 and 11,937 at 18 months and 5,317 and 6,478 at 24 months

Supplementary Figure S6:Odds of participants acquiring new LTCs at 6, 12, 18 and 24-month follow-up periods split by age group

Under 70 years

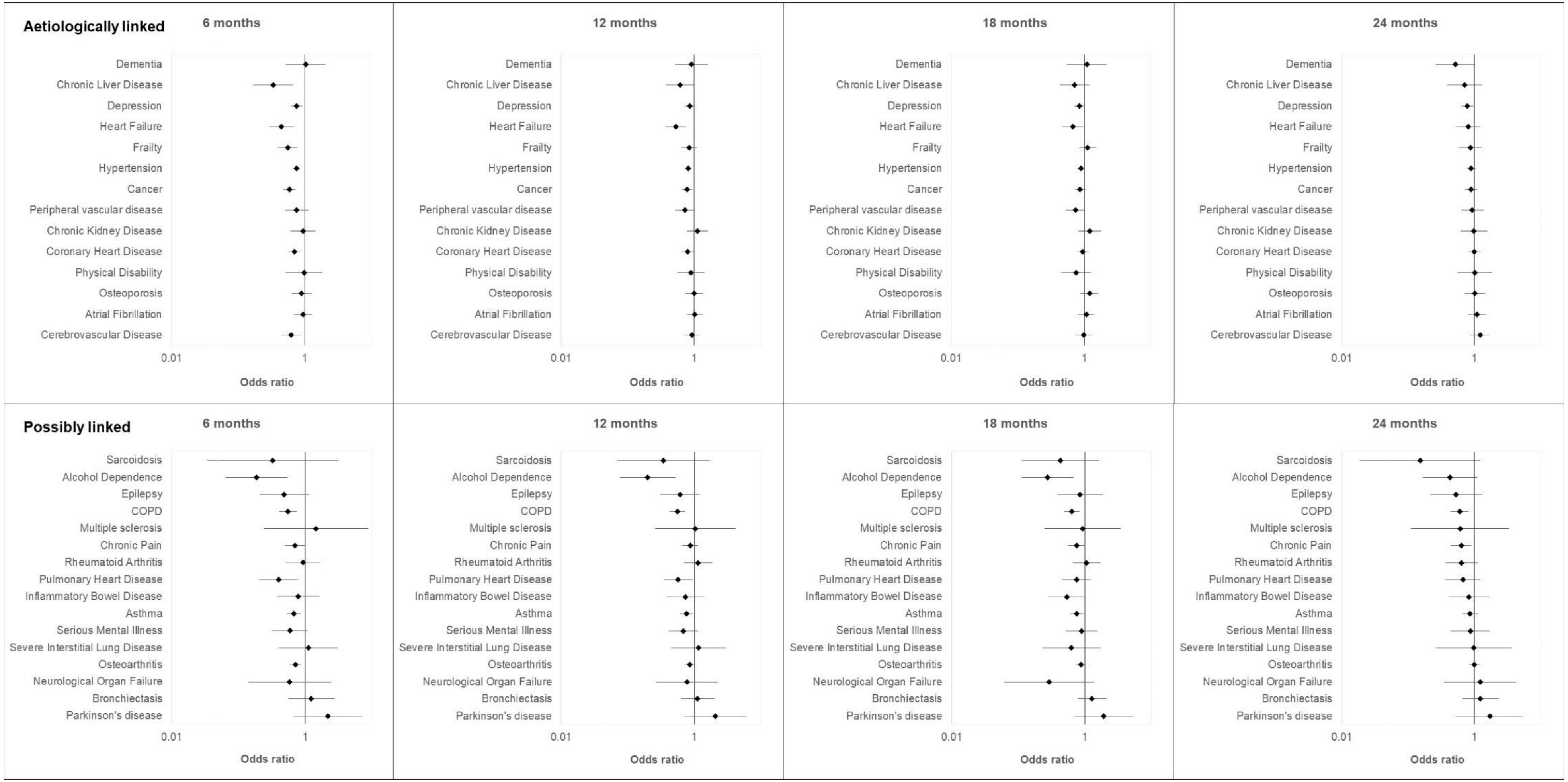

Data are presented as odds ratios with 95% confidence intervals. N (intervention and control) = 33,453 and 32,679 at 6 months, 22,070 and 22,662 at 12 months, 12,951 and 14,996 at 18 months and 6,799 and 8,235 at 24 months

70 years and over

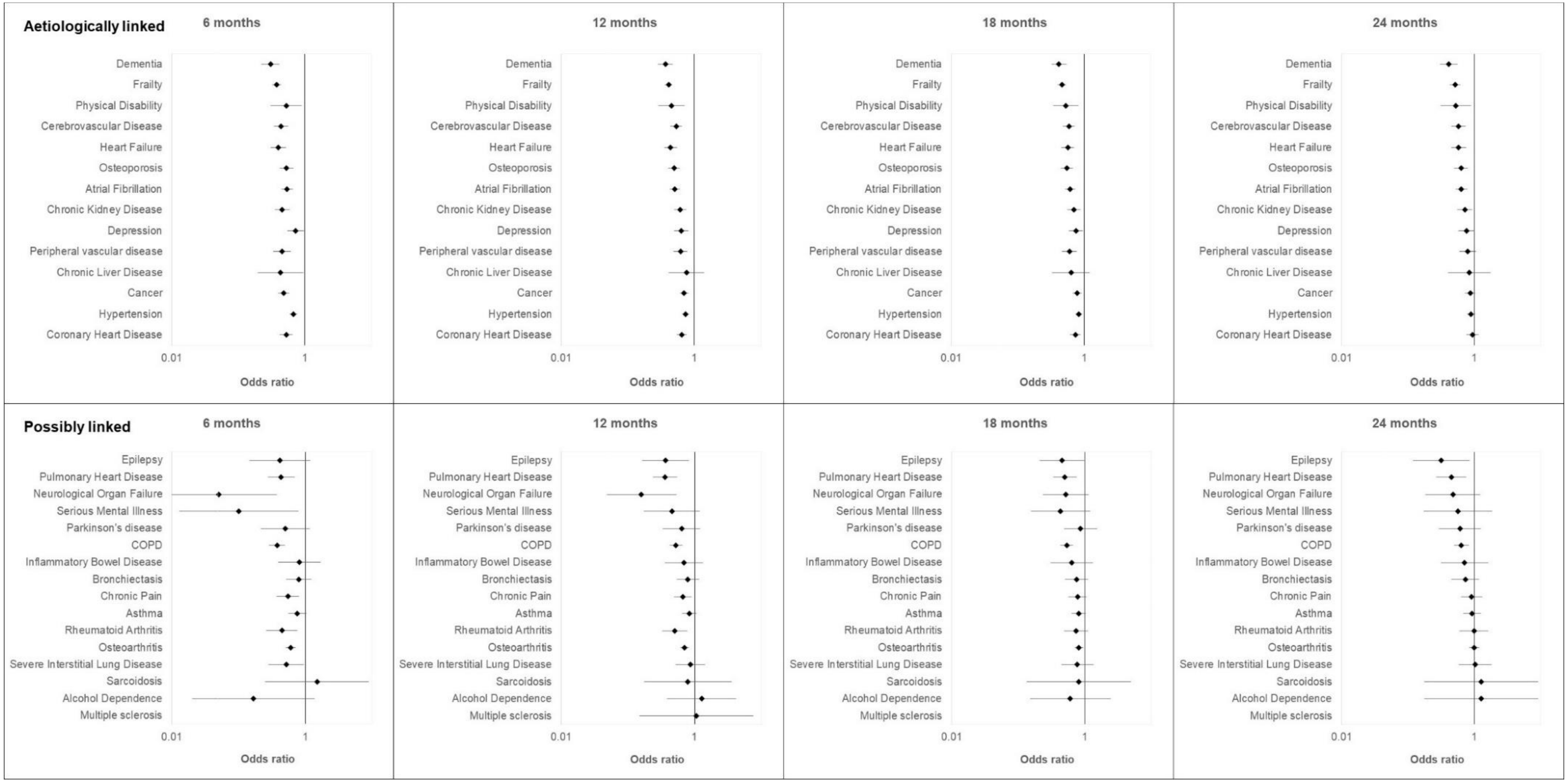

Data are presented as odds ratios with 95% confidence intervals. N (intervention and control) = 23,487 and 23,636 at 6 months, 16,070 and 17,111 at 12 months, 9,546 and 11,604 at 18 months and 5,022 and 6,359 at 24 months
